# Large-scale genomic surveillance reveals immunosuppression drives mutation dynamics in persistent SARS-CoV-2 infections

**DOI:** 10.1038/s41467-026-74445-7

**Published:** 2026-06-19

**Authors:** Mark P. Khurana, Alexandros Katsiferis, Neil Scheidwasser, Mathilde Marie Brünnich Sloth, Jacob Curran-Sebastian, Christian Morgenstern, Man-Hung Eric Tang, Jannik Fonager, Morten Rasmussen, Marc Stegger, Sune Lehmann, Charles Whittaker, Laust H. Mortensen, Pikka Jokelainen, Moritz U. G. Kraemer, Neil M. Ferguson, Mahan Ghafari, Tyra G. Krause, David A. Duchêne, Samir Bhatt

**Affiliations:** 1 Section for Health Data Science and AI, Department of Public Health, University of Copenhagen, Copenhagen, Denmark; 2 Department of Infectious Disease Epidemiology, School of Public Health, Imperial College London, London, UK; 3 Data Science Section, IT University of Copenhagen, Copenhagen, Denmark; 4 Department of Sequencing and Bioinformatics, Statens Serum Institut, Copenhagen, Denmark; 5 Department of Virology and Microbiological Preparedness, Statens Serum Institut, Copenhagen, Denmark; 6 Antimicrobial Resistance and Infectious Diseases Laboratory, Harry Butler Institute, Murdoch University, Murdoch, Australia; 7 Department of Applied Mathematics and Computer Science, Technical University of Denmark, Kongens Lyngby, Denmark; 8 Division of Infectious Diseases and Vaccinology, School of Public Health, University of California, Berkeley, CA USA; 9 Center for Computational Biology, College of Computing, Data Science, and Society, University of California, Berkeley, CA USA; 10 Statistics Denmark, Copenhagen, Denmark; 11 ROCKWOOL Foundation, Copenhagen, Denmark; 12 Section of Epidemiology, Department of Public Health, University of Copenhagen, Copenhagen, Denmark; 13 Infectious Disease Preparedness and One Health, Statens Serum Institut, Copenhagen, Denmark; 14 Department of Biology, University of Oxford, Oxford, United Kingdom; 15 Pandemic Sciences Institute, University of Oxford, Oxford, United Kingdom; 16 Infectious Disease Epidemiology and Prevention, Statens Serum Institut, Copenhagen, Denmark

**Keywords:** Risk factors, Viral infection, Next-generation sequencing

## Abstract

Persistent SARS-CoV-2 infections have been hypothesized to play a key role in the emergence of variants of concern. However, the factors determining which individuals are at risk and their viral molecular signatures during infection remain poorly understood. Using Denmark’s extensive COVID-19 surveillance, comprising over 700,000 genomes, we identify 303 persistent infections and, critically, link them to health and sociodemographic data. Our analysis confirms the hypothesis that immunocompromised individuals are at the highest risk of experiencing persistent infections. Other disease groups associated with mortality, such as diabetes, show no such associations. Among these persistent infections, the viral sequences exhibit signs of positive selection, with recurrent mutations linked to treatment resistance. Our findings suggest that immunosuppression plays a key role in the emergence of novelty in persistent infections.

## Abstract (web summary)

Using Denmark’s genomic surveillance linked to health registries, the authors identify immunosuppression as a key risk factor for persistent SARS-CoV-2 infections and show these infections accumulate mutations associated with treatment resistance.

## Introduction

Novel variants of concern are increasingly thought to arise in individuals with persistent infections, particularly those with compromised immune systems who are unable to clear an infectious episode^1–7^. In a persistent SARS-CoV-2 infection, defined as lasting 26 days or more, a threshold reflecting that most acute infections clear within 20-30 days^8^, the virus is thought to maintain greater within-host diversity than in acute infections^6,8,9^. Persistently infected individuals are more likely to receive treatment during their infections, potentially intensifying selective pressure on the virus^6,10,11^. Persistent infections are also frequently found in individuals with compromised immune systems^2,4,6,7,12^, and are linked with selection for amino-acid changes that can define new virus lineages^8^ and which occur at target sites for monoclonal antibodies^5,8,13–19^. Cryptic lineages found in wastewater samples are also likely shed by individuals with persistent infections, because the prolonged infections allow accumulation of unique mutational signatures not typically observed in acute cases^20–22^. Nonetheless, the factors that predispose individuals to persistent SARS-CoV-2 infections remain incompletely understood, including host characteristics, immune status, and comorbidities^23–26^. Moreover, persistent infections may provide an environment that facilitates the accumulation of mutations and the emergence of novel viral variants, although the underlying mechanisms and contributions of specific clinical conditions remain unclear.

Genomic surveillance is shifting how we detect and study pathogen spread and evolution^27,28^. Research on within-host viral evolution is usually based on small sample sizes (e.g., case studies)^2,3,5^, or on large cohort studies that lack individual-level data beyond sociodemographic characteristics^8,19^, such as patient history and health condition. When aiming to prevent the emergence of new variants via mitigation strategies at their source, it is critical to describe the scenarios of emergence and corresponding patient risk factors^8^. Extensive genomic surveillance coupled with detailed centralized registry data can provide unique insights into the settings linked with the emergence of variants of concern^29^.

Here, we leverage Denmark’s extensive genomic surveillance database, coupling it with national registry data to identify persistent SARS-CoV-2 infections and individual-level sociodemographic data and medical diagnoses. Using consensus-level sequence data, we inferred individuals with SARS-CoV-2 infection episodes spanning 26 days or more, based on repeated sampling of the same variant and shared rare single-nucleotide polymorphisms (SNPs). We identify risk factors associated with persistent infections using a case-control study design. Further, we describe the mutational patterns associated with these infections and identify characteristics and health conditions linked with accelerated selective genetic changes in the virus. By linking diagnostic health information directly with pathogen evolution across population-level data, our analyses assert new value to centralized health registries and epidemiological genomic surveillance efforts.

## Results

### Identifying persistent infections

To identify persistent SARS-CoV-2 infections, we analyzed all high-quality viral sequences collected by the Danish COVID-19 Genome Consortium (DCGC) from 2020 to 2024 (*N* = 738944) (Supplementary Fig. 1). This dataset represents one of the largest and most representative sampling efforts conducted globally to date^29^. Only consensus sequences from the Alpha, Delta, and Omicron variants (BA.1, BA.2, and BA.5) were included due to a relative paucity of samples for the “wild-type" variant. For each individual, sequence pairs within the same variant sampled at least 26 days apart were considered indicative of potentially persistent infections. Infection episodes were defined based on consensus sequences preceding and following each pair (see “Methods”). Consensus sequences were derived from reverse transcription (RT-PCR)-positive samples, using low cycle threshold (Ct) values as indicators of viral RNA load. Sequences with Ct ≤ 38 were retained, following DCGC guidelines for high-quality sequences included in the national sequencing database. This process yielded 455 potential persistent infections.

**Fig. 1:**
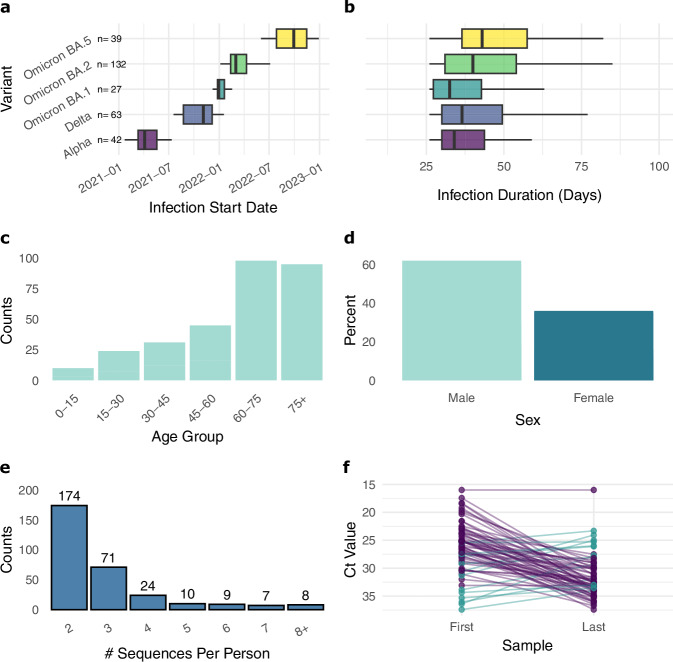
Characteristics of the study population (*N* = 303). **a** Date of first sequence for each infection stratified by variant. **b** Time since first and last sequence for each infected individual. In both panels, box plots show the median (center line), interquartile range (IQR; box bounds, 25th–75th percentiles), and whiskers extending to the most extreme data points within 1.5  × IQR of the box. Outliers beyond the whiskers are not displayed. **c** Number of individuals in each age group. **d** Number of individuals of each sex. **e** Number of available sequences per infection. **f** Cycle threshold (Ct) values at first and last sample for each individual. Purple lines indicate no change or an increase in Ct values (i.e., decrease in viral load), while green lines indicate a decrease in Ct values (i.e., increase in viral load). An increase in viral load was observed in 18.6% of individuals. Source data underlying this figure can be generated using the custom code available at the repository described in the Code Availability statement.

We then filtered the resulting subset of persistent infections to retain only those where consensus sequences shared the same rare SNPs at one or more sites, relative to the population-level consensus of the major lineage, as previously described by Ghafari et al.^8^. Using our rare SNP cut-off, the false positive rate (i.e., the proportion of incorrectly identified persistent infections among randomly paired sequences) was estimated to be between 2.1% and 3.3%, resulting in 303 persistent infections (42 Alpha, 63 Delta, 27 BA.1, 132 BA.2, 39 BA.5) (Fig. 1a and Supplementary Table 1). Thus, 152 cases were excluded for not meeting the rare SNP-based filtering criteria. 20 infections lasted  > 100 days, and five  > 150 days (Fig. 1b), consistent with previously identified upper bounds for some persistent infections^6,8^. There was a preponderance of older, male participants among the included cases (Fig. 1c, d). Ct values, inversely proportional to viral RNA load, were higher at the final sequenced time point compared to the initial one (mean change in Ct = − 5.28 [95% credible interval (CrI):  − 6.64, − 3.94]) (Fig. 1f).

### Risk factors for persistent infections

To identify risk factors for persistent SARS-CoV-2 infections, we conducted a matched case-control study. Each case of persistent infection was matched with controls who met the following criteria: a positive PCR test within  ± 1 day of the case’s infection start date and the same major variant, a negative test within 26 days of that positive result, and no additional positive tests within the subsequent 120 days. We identified a total of 146, 904 controls across all strata, corresponding to 62, 648 unique individuals. This corresponds to a median of 401 (interquartile range, IQR 162–522) controls per case. However, for 9 cases, we found fewer than 10 matches, resulting in a range of 0 to 8 matches for these cases. Using Bayesian uni- and multi-variable conditional logistic regression models^30^, we assessed associations between persistent infection and key factors, including age, sex, vaccination status, Charlson Comorbidity Index (CCI)^31^, and specific medical conditions (using ICD-10 codes): immunosuppression, autoimmune disorders, immune-mediated inflammatory diseases (IMID), and diabetes. To avoid confounding from vaccination rollout and pandemic-related changes in diagnostic patterns, only pre-2020 diagnoses were included, with contemporary diagnoses explored in supplementary analyses.

Compared to individuals aged 15–30, those in the 45–60, 60–75, and 75 + age groups had significantly higher odds of experiencing persistent SARS-CoV-2 infections (adjusted odds ratio, aOR 1.96 [CrI 1.22, 3.09], 6.59 [CrI 4.37, 10.09], and 10.13 [CrI 6.62, 15.70], respectively), while no significant differences were observed for the 0–15 and 30–45 age groups. In addition, females were significantly less likely to experience persistent infection compared to males (aOR 0.53 [CrI 0.42, 0.67]) (Table 1). Compared to unvaccinated individuals, those with 2 or 3 vaccinations less than 18 weeks post-vaccination had a reduced odds of persistent infection by 46% (aOR 0.54 [CrI 0.35, 0.84]). However, individuals with one vaccination or 2-3 vaccinations ≥ 18 weeks post-vaccination showed no protection. Using a 10-year CCI lookback period resulted in similar estimates (Supplementary Table 2), as well as using contemporary 5-year CCI scores (Supplementary Table 3).

**Table 1:** Risk factors for persistent infection

Variable		Cases (Median [IQR])	Controls (Median [IQR])	Unadjusted OR (95% CrI)	Adjusted* OR (95% CrI)
**Age Group**	0–15	24	32667	0.61 (0.31–1.16)	0.58 (0.27–1.14)
	15–30	10	24666	Ref.	Ref.
	30–45	31	31606	1.22 (0.74–1.96)	1.28 (0.78–2.06)
	45–60	46	29477	1.96 (1.27–3.08)	1.96 (1.22–3.09)
	60–75	98	17615	6.88 (4.65–10.59)	6.59 (4.37–10.09)
	75 +	94	10873	10.47 (6.92–16.47)	10.13 (6.62–15.70)
**Sex**	Male	194	68774	Ref.	Ref.
	Female	109	78130	0.48 (0.38–0.62)	0.53 (0.42–0.67)
**Vaccination status**	No vaccination	76	56497	Ref.	Ref.
	1 Vaccination	8	6520	0.94 (0.43–1.83)	0.65 (0.30–1.27)
	2 or 3 Vaccinations, < 18 weeks	90	52046	1.27 (0.86–1.91)	0.54 (0.35–0.84)
	2 or 3 Vaccinations, ≥ 18 weeks	129	31841	2.17 (1.45–3.29)	0.80 (0.53–1.24)
**Charlson Comorbidity Index†**	Per 1 Increase	0 [0–2]	0 [0-0]	1.55 (1.47–1.64)	1.35 (1.26–1.43)

Values are presented as median [IQR] for continuous variables and *N* for categorical variables. Unadjusted and adjusted odds ratios (OR) with 95% credible intervals (CrI) are presented from the conditional logistic regression output, with adjustments for all other variables. CrI = credible interval; IQR = interquartile range. The Charlson Comorbidity Index was calculated with a 5-year lookback from January 1, 2020. Vaccination status combines the number of vaccinations at infection onset and the time since the last vaccination (< 18 weeks or ≥ 18 weeks) for those with 2 or 3 vaccinations. The total number of controls is *N *= 146, 904, corresponding to *N *= 62, 648 unique individuals. *Models for age and sex were mutually adjusted; the Charlson Comorbidity Index model was adjusted for age and sex; the vaccination model was adjusted for all other variables; † 5-year lookback from 2020-01-01.

Among individuals with specific medical conditions, immunosuppressed individuals had significantly higher odds of persistent infection (aOR 5.84 [CrI 4.73, 7.18]), consistent with findings from prior individual-level studies on persistent infections^1–6^. Those with autoimmune disorders had a marginally elevated risk of persistent infection (aOR 1.57 [CrI 1.01, 2.29]) (Table 2), although we found no evidence of increased risk in a multivariate model when adjusting for all other diagnoses (Supplementary Table 4). No increased risk was observed among individuals with diabetes or immune-mediated inflammatory diseases (aOR 1.10 [CrI 0.81, 1.45] and 1.13 [CrI 0.79, 1.58], respectively) (Table 2). We found no significant interactions between diagnoses and age or sex. We repeated the analysis using contemporary diagnoses (i.e., those registered up to 2021-12-31, for which diagnostic registry data is available), yielding similar results (Supplementary Table 5).

**Table 2:** Diagnoses associated with persistent infection

Diagnosis Group†		Cases	Controls	Unadjusted OR (95% CrI)	Adjusted* OR (95% CrI)
**Model 1: Immunosuppression**	Yes	76	1929	7.91 (6.47–9.73)	5.84 (4.73–7.18)
	No	227	144975	Ref.	Ref.
**Model 2: Autoimmune**	Yes	13	1611	2.16 (1.38–3.24)	1.57 (1.01–2.29)
	No	290	145293	Ref.	Ref.
**Model 3: Diabetes**	Yes	28	4717	1.81 (1.35–2.37)	1.10 (0.81–1.45)
	No	275	142187	Ref.	Ref.
**Model 4: IMID**	Yes	18	4775	1.38 (0.98–1.89)	1.13 (0.79–1.58)
	No	285	142129	Ref.	Ref.

Values are presented as *N* for categorical variables. Unadjusted and adjusted odds ratios (OR) with 95% credible intervals (CrI) are presented from the conditional logistic regression output. IMID = immune-mediated inflammatory diseases. Diagnoses are based on pre-pandemic conditions (i.e., those prior to January 1, 2020). The total number of controls is *N *= 146, 904, corresponding to *N *= 62, 648 unique individuals. *Each model is also adjusted for sex and age. † Using pre-pandemic diagnoses (i.e., those prior to 2020-01-01).

### Mutational landscape and frequent amino-acid changes

**Fig. 2:**
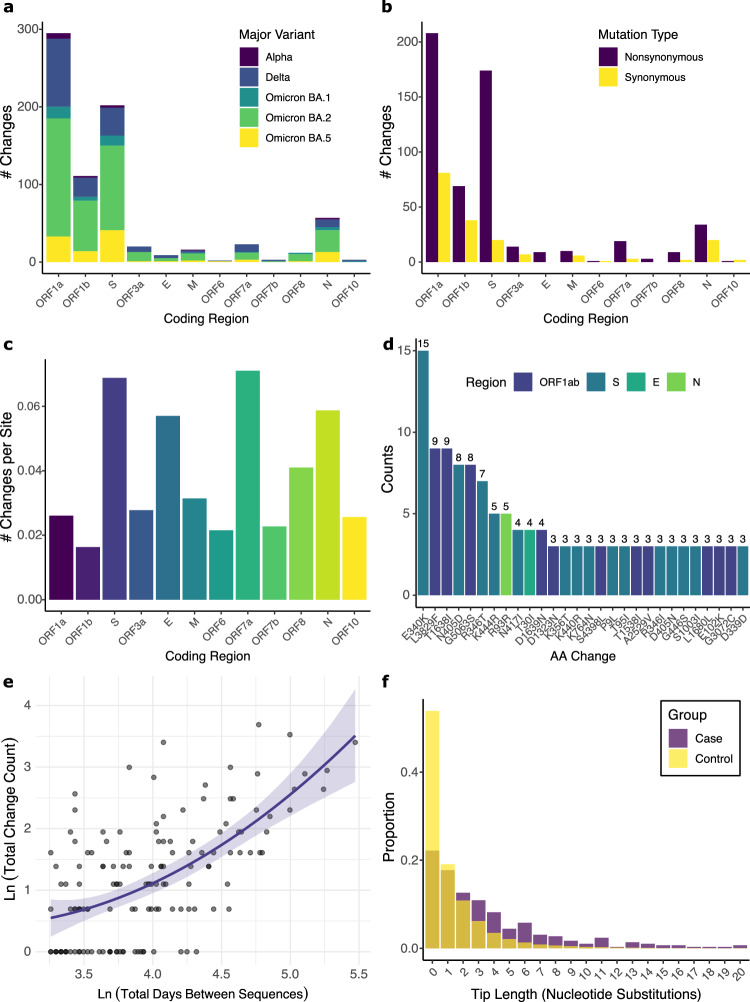
Comparison of coding region substitutions and evolutionary dynamics in persistent infections. **a** Location of substitutions in each coding region. **b** Location of (non)synonymous substitutions in each coding region. **c** Number of nucleotide substitutions in each coding region normalized per site. **d** Location of recurrent amino acid substitutions. **e** Scatter plot illustrating the relationship between log-transformed total days between the first and last sequences and log-transformed total change count (including non-coding regions), excluding points without changes (*N* = 143). The Pearson correlation coefficient is 0.612. Two Bayesian models were fitted using the *brms*^86^ package: a linear-log model and a quadratic-log model, with the line representing the posterior mean prediction from the quadratic model. The shaded area denotes the 95% Bayesian credible interval for the model prediction. Model comparison via Pareto Smoothed Importance Sampling Leave-One-Out-cross-validation (PSIS-LOOCV) showed that the quadratic model was a better fit to the data than the linear model (expected log pointwise predictive density (ELPD) difference:  − 0.8). **f** Histogram comparing tip lengths of persistent infections to acute controls. Independent phylogenetic trees were constructed for each case-control set (from conditional logistic regression) with at least 10 controls (*N* = 293). Source data underlying this figure can be generated using the custom code available at the repository described in the Code Availability statement.

The majority of the within-host mutations observed between consecutive consensus sequences in each persistent infection occurred in the ORF1ab (open reading frame 1ab) and S (spike) coding regions of the SARS-CoV-2 genome (Fig. 2a, b). The highest mutation counts per site were found in the ORF7a, S, N, and E coding regions (Fig. 2c). A total of 148 infections (48.8%) showed no consensus-level mutations between any pairs of consecutive sequences in coding regions during their infections. In addition, 175 infections (57.8%) showed no change between their first and last sequences in coding regions, and 143 infections (47.2%) showed no change when including non-coding regions. For those with at least one consensus-level change, the mean genome-wide rate was 1.03 × 10^−3^ substitutions per site per year (95% Confidence Interval, CI 8.64 × 10^−4^ to 1.19 × 10^−3^), similar to previously reported rates^32^. Across all infections and pairs of consecutive sequences, we observed 679 non-synonymous (amino acid-changing) and 209 synonymous (silent) mutations in coding regions (Supplementary Fig. 2); comparing only the first and last sequences in each infection revealed 551 non-synonymous and 180 synonymous mutations (Fig. 2b).

We also found that tip lengths from persistently infected individuals were significantly longer than those of their matched controls, indicating that these infections continue to accumulate mutations over time (Fig. 2e) and contribute measurable divergence within the population phylogeny ($\widehat{\beta }=-2.24$ nucleotide substitutions for acute versus persistently infected individuals, [CrI  − 2.69, − 1.79]) (Fig. 2f). We then compared the final sequence from each persistent infection, focusing on those with at least one nonsynonymous change relative to the initial sequence, to sequences from acute cases in the COVID-19 database sampled within 11 days (the upper limit of the virus’ generation time)^33^. Among the persistent cases, 10 matched exactly (0 differences) with 33 sequences in the database, with many matches concentrated in the same Danish regions as the persistent infections, consistent with potential local onward transmission (Supplementary Table 6). 17 cases matched with one difference (248 sequences), and 27 matched with two differences (5239 sequences). While these similarities do not confirm direct epidemiological transmission links, they indicate that persistent infections remain genetically consistent with circulating variants, supporting the view that onward transmission remains plausible even in the presence of nonsynonymous changes.

**Fig. 3:**
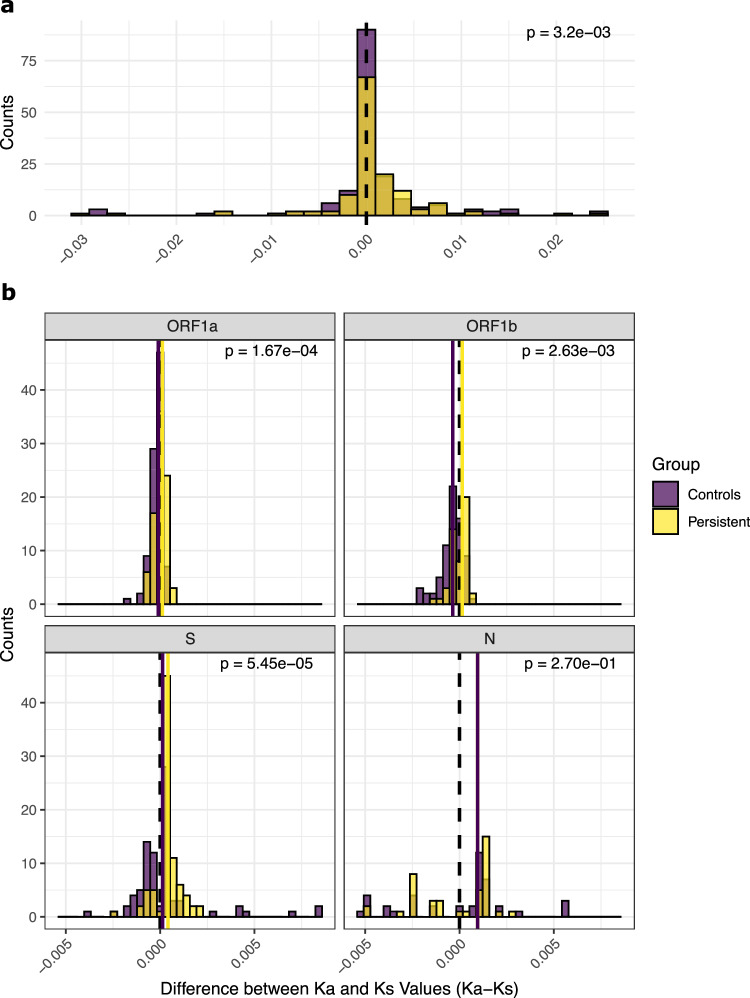
Differences in Ka and Ks values (Ka - Ks) across the genome and by coding region. **a** Difference in Ka and Ks values among individuals with persistent infections (*N* = 135) and among individuals without persistent infections (controls, *N* = 167) with two or more available sequences. Ka and Ks represent the substitution rates per non-synonymous and synonymous site, respectively. The dotted line indicates a value of 0, and the solid lines represent median values. The p-value denotes the p-value from a two-sided two-sample Kolmogorov-Smirnov (KS) test comparing the distributions between the two groups. No adjustments were made for multiple comparisons. **b** Difference in Ka and Ks values in each coding region among individuals with and without persistent infections, specifically those with at least one mutation in the region. P-values are from KS tests as above. Source data underlying this figure can be generated using the custom code available at the repository described in the Code Availability statement.

While we did not observe evidence of genome-wide positive selection in persistent infections (Ka and Ks difference, *β* = − 3.23 × 10^−3^, CrI:  − 1.99 × 10^−2^ to 1.47 × 10^−2^) (Fig. 3a), we found strong evidence of selection in the S coding region (*β* = 4.15 × 10^−4^, CrI: 2.70 × 10^−4^ to 5.64 × 10^−4^) (Fig. 3b). We also found evidence of positive selection in the ORF7a (*β* = 3.59 × 10^−3^, CrI: 3.98 × 10^−4^ to 6.65 × 10^−3^) and E (*β* = 6.59 × 10^−3^, CrI: 5.94 × 10^−3^ to 7.25 × 10^−3^) coding regions (Supplementary Fig. 3), although few observations were available. We found no evidence of positive or negative selection in other coding regions (Supplementary Table 7). In controls with multiple available sequences, we observed evidence of negative (purifying) selection in ORF1a (*β* = − 1.59 × 10^−4^, CrI:  − 2.32 × 10^−4^ to  − 8.75 × 10^−5^) and ORF1b (*β* = − 4.32 × 10^−4^, CrI:  − 5.64 × 10^−4^ to  − 3.03 × 10^−4^), with no evidence of genome-wide selection (Fig. 3a; Supplementary Table 8).

**Fig. 4:**
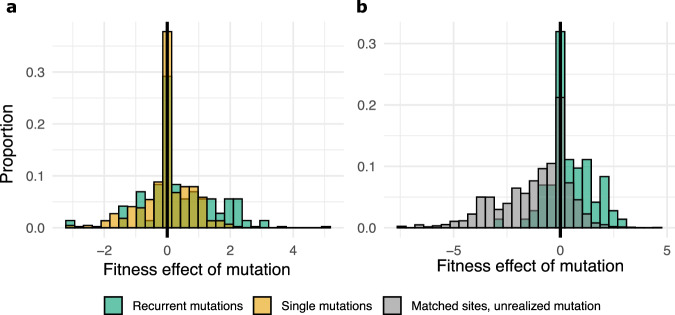
Distribution of fitness effects for recurrent and non-recurrent, single mutations. **a** Comparison of recurrent mutations (*N* = 72) versus single-appearing mutations (*N* = 446) in persistently infected individuals. **b** Comparison of recurrent mutations with other amino acids found at the same sites. Fitness values represent the potential for these mutations to spread between hosts, as determined from a globally representative phylogeny^34^. Source data underlying this figure can be generated using the custom code available at the repository described in the Code Availability statement.

Recurrent mutations in persistent infections exhibited substantially higher transmission fitness values than single-appearing mutations (Fig. 4). These independently-derived fitness values measure the potential of mutations to spread between hosts (i.e., transmission-associated fitness) based on the ratio of observed to expected occurrences in a global SARS-CoV-2 phylogeny by Bloom et al.^34^. The approach assumes that non-deleterious mutations arise independently across the phylogenetic tree, with deviations from expectations reflecting their effects on transmission; higher values indicate mutations more likely to spread. We found that single-appearing mutations were associated with a mean fitness decrease of  − 0.40 (CrI  − 0.62, − 0.18) relative to recurrent mutations, while other amino acids at matched sites showed a mean fitness decrease of  − 1.50 (CrI  − 1.86, − 1.12) relative to recurrent mutations.

The most common recurrent mutations observed between the first and last sequences in persistent infections were: S:E340K (nucleotide substitution G22580A; *N* = 15), ORF1a:T1638I (C5178T; *N* = 9), ORF1ab:L3829F (C11750T; *N* = 9), S:N405D (A22775G; *N* = 8), ORF1ab:G5063S (G15451A; *N* = 8), and S:R346T (G22599C, *N* = 7) (Fig. 2d) (Supplementary Table 9). These broadly correspond to those observed when comparing all pairs of consecutive sequences (Supplementary Table 10). Notably, S:N405D was also the most frequent recurrent mutation in another large persistent infection study^19^, and forms part of the BA.2 spike protein. S:E340K has been linked with sotrovimab resistance, particularly in immunocompromised patients^5,16^.

We also noted several recurring amino acid changes that have previously been linked with monoclonal antibody resistance, including S:R346T, S:K444R (A22893G, *N* = 5), S:R346I (G22599T, *N* = 3), S:K356T (A22629C, *N* = 3) and S:S494P (T23042C, *N* = 2)^8,11,13–15,17,35–37^. ORF1ab:L3829F (C11750T) has also been associated with nirmatrelvir/ritonavir treatment^38^, and ORF1ab:V5184I (G15814A, *N* = 4 when comparing consecutive sequences [Supplementary Table 10]) with resistance to remdesivir ^39–41^. Similarly, we observed several amino acid changes associated with molnupiravir resistance, including S:K440R (A22881G, *N* = 3) and S:P9L (C21588T, *N* = 3)^42,43^. The timings of observed mutations also align with the corresponding use and introduction of these medications in Denmark, increasing the plausibility of these being directly linked to treatment initiation (Supplementary Table 11). ORF1ab:G5063S (G15451A) has also been associated with higher mortality in Delta patients^44^. Lastly, we also observed several other mutations previously identified in immunocompromised patients, including ORF1a:T1638I (C5178T), E:T30I (C26333T, *N* = 4), S:N417I (A22812T, *N* = 4) and S:T95I (C21846T, *N* = 3)^2,6,8,12^.

### Risk factors associated with within-host substitutions

To investigate which host factors might influence viral evolution during persistence, we compared the first and last sequences for each persistent infection. A baseline model was developed using zero-inflated negative binomial regression, with stepwise AIC (Akaike information criterion) employed to identify relevant characteristics. The model regressed these characteristics against the number of synonymous and nonsynonymous changes, adjusting for the time between samples as a covariate (Supplementary Table 12). For every additional day between samples, the number of absolute nonsynonymous changes across all coding region amino acid sites increased by 0.023 (Standard error (SE) = 0.002, *p* < 2 × 10^−16^); similarly, the number of synonymous changes increased by 0.019 (*SE* = 0.003, *p* = 1.27 × 10^−13^). Compared to the Alpha variant, genomes in individuals infected with Delta, Omicron BA.1, BA.2, and BA.5 showed significantly more nonsynonymous changes: Delta ($\widehat{\beta }=1.751$, *SE* = 0.381, *p* = 4.25 × 10^−6^), BA.1 ($\widehat{\beta }=1.446$, *SE* = 0.437, *p* = 9.39 × 10^−4^), BA.2 ($\widehat{\beta }=1.620$, *SE* = 0.381, *p* = 2.15 × 10^−5^), and BA.5 ($\widehat{\beta }=2.100$, *SE* = 0.400, *p* = 1.59 × 10^−7^). In contrast, rates of synonymous changes did not differ significantly between these variants. This difference likely reflects varying selection pressures as novel treatments, including monoclonal antibody and antiviral therapies, became more prevalent with later variants^45,46^. While increasing vaccination rates could also increase selection pressure, as vaccination alters the host immune landscape and may favor variants with enhanced immune evasion, we found no evidence that the number of vaccinations was associated with the amount of (non)synonymous change (Supplementary Table 13) or indeed the genome-wide substitution rate (Supplementary Table 14), resulting in their omission for the baseline model.

We next examined how demographic factors and health status may influence within-host evolution. Notably, older age groups exhibited a smaller number of both nonsynonymous and synonymous changes in the viral genome compared to those in the 15-30 age group (Supplementary Table 12). Specifically, virus genomes in individuals aged 45–60 (nonsynonymous $\widehat{\beta }=-0.842$, *SE* = 0.301, *p* = 5 × 10^−3^; synonymous $\widehat{\beta }=-1.063$, *SE* = 0.388, *p* = 0.006 × 10^−3^), 60–75 (nonsynonymous $\widehat{\beta }=-1.237$, *SE* = 0.257, *p* = 2.15 × 10^−5^; synonymous $\widehat{\beta }=-1.273$, *SE* = 0.315, *p* = 5.26 × 10^−5^), and 75 + (nonsynonymous $\widehat{\beta }=-1.127$, *SE* = 0.273, *p* = 1.59 × 10^−5^; synonymous $\widehat{\beta }=-1.451$, *SE* = 0.342, *p* = 2.18 × 10^−5^) showed lower numbers of nonsynonymous and synonymous changes than in the 15–30 years group. Lastly, no association was found between CCI and the number of synonymous changes. However, there was evidence suggesting that higher CCI scores were linked to an increase in nonsynonymous changes ($\widehat{\beta }=0.145$, *SE* = 0.046, *p* = 2 × 10^−3^). Similar results were found when only using pre-pandemic diagnoses (Supplementary Table 15).

We also examined the impact of various diagnoses on the number of nonsynonymous and synonymous changes, adjusting for time between samples, age and major variant. Genomes in individuals with an immunosuppression diagnosis had significantly more nonsynonymous changes than those without ($\widehat{\beta }=0.564$, *SE* = 0.099, *p* = 1.30 × 10^−8^), while showing similar numbers of synonymous changes ($\widehat{\beta }=0.027$, *SE* = 0.152, *p* = 0.859) (Table 3). Other diagnoses, including autoimmune disorders (nonsynonymous $\widehat{\beta }=-0.010$, *SE* = 0.201, *p* = 0.960; synonymous $\widehat{\beta }=-0.198$, *SE* = 0.318, *p* = 0.533), diabetes (nonsynonymous $\widehat{\beta }=-0.170$, *SE* = 0.178, *p* = 0.338; synonymous $\widehat{\beta }=-0.435$, *SE* = 0.324, *p* = 0.180), and immune-mediated inflammatory diseases (IMID) (nonsynonymous $\widehat{\beta }=-0.339$, *SE* = 0.238, *p* = 0.155; synonymous $\widehat{\beta }$ =  − 0.006, SE = 0.321, *p* = 0.986), were not associated with increased numbers of either nonsynonymous or synonymous changes. Similar results were obtained when analyses were restricted to pre-pandemic diagnoses (Supplementary Table 16).

**Table 3:** Regression results for nonsynonymous and synonymous changes by diagnostic group

Diagnosis Group†	Nonsynonymous Changes			Synonymous Changes		
	$\widehat{\beta }$	SE	p-value	$\widehat{\beta }$	SE	*p*-value
**Model 1: Immunosuppression**	0.564	0.099	1.30 × 10^−8^	0.027	0.152	0.859
**Model 2: Autoimmune**	− 0.010	0.201	0.960	− 0.198	0.318	0.533
**Model 3: Diabetes**	− 0.170	0.178	0.338	− 0.435	0.324	0.180
**Model 4: IMID**	− 0.339	0.238	0.155	− 0.006	0.321	0.986

Coefficients ($\widehat{\beta }$), standard errors (SE), and *p*-values are from a zero-inflated negative binomial regression model, with each model adjusted for time between samples, major variant, and age group. Diagnoses are based on contemporary data (up to December 31, 2021). Estimates denote the absolute number of (non)synonymous changes across all coding regions relative to the reference group. Estimates were adjusted for time between samples, major variant and age group. † Using contemporary diagnoses, i.e., those registered up to 2021-12-31, for which diagnostic data is available. IMID = Immune-mediated inflammatory diseases.

We further explored whether treatment resistance-linked mutations occurred preferentially among immunosuppressed individuals. Among individuals with amino acid substitutions in the viral genome associated with monoclonal antibody resistance, the majority also had an immunosuppression diagnosis (Supplementary Table 17). Similarly, for S:K440R (3/3, 100%) and S:P9L (3/3, 100%), both associated with molnupiravir resistance, all individuals had an immunosuppression diagnosis. For substitutions associated with nirmatrelvir/ritonavir resistance, such as ORF1ab:L3829F, 4/7 (57.1%) of individuals had an immunosuppression diagnosis (Supplementary Table 17). Among individuals with monoclonal antibody resistance-linked substitutions whose infections ended prior to the mid-2022 cutoff for hospitalization data, 58.3% (7 of 12) were hospitalized during their infection.

Finally, we examined whether immunosuppression influenced the likelihood of viral rebound, defined as a negative RT-PCR test followed by a subsequent positive test during infection. The overall rebound rate was 18.2% (55/303) across all persistent infections. Among non-immunosuppressed individuals, 20.4% (38/186) experienced rebound, compared to 14.5% (17/117) of immunosuppressed individuals. Using a Bayesian logistic regression model, we found no evidence of a difference in the likelihood of rebounding between immunosuppressed and non-immunosuppressed individuals (*β* = − 0.28, CrI:  − 0.72 to 0.15) (Supplementary Table 18), even after adjusting for infection duration (*β* = − 0.29, CrI:  − 0.75 to 0.15) (Supplementary Table 19). In addition, rebounding was not associated with the substitution rate (Supplementary Table 20).

### Discussion

In this study, we identified risk factors associated with persistent SARS-CoV-2 infections by analyzing a comprehensive dataset from Denmark, including detailed associated metadata. Our results indicate that older age groups, particularly individuals aged 45 years and above, are markedly more likely to develop persistent infections compared to younger individuals. In addition, we find that individuals with an immunosuppression diagnosis faced over five times the risk of experiencing persistent infections, underscoring the heightened vulnerability of this population. Our analysis also revealed several mutations associated with antibody and antiviral resistance, and significant selective pressures in several coding regions not observed in acute infections, suggesting ongoing viral adaptation. Notably, immunosuppression appeared to contribute to an increased accumulation of nonsynonymous mutations, whereas these dynamics were not associated with other disease groups.

Our findings on sociodemographic characteristics are consistent with those of other studies, which also report that older age groups and males are at a higher risk of developing persistent infections upon testing positive^19^. Importantly, our findings also provide evidence that full vaccination, prior to immune waning, reduces the likelihood of an infection becoming persistent. This builds on previous findings suggesting that vaccination reduces viral diversity during infection, limiting the exploration of protein sequence variations^47–49^. This could lower the likelihood of novel variants and immune escape, thereby helping to prevent infections from becoming persistent. While older individuals had higher odds of persistent infection, they paradoxically exhibited fewer within-host substitutions. This may reflect immune senescence, whereby aging impairs innate and adaptive immune responses, which can prolong infections while exerting weaker selective pressure to drive viral diversification^50^.

We found that immunosuppression and autoimmune disorders were risk factors for persistent infections, whereas no association was observed between diabetes, IMID, and persistent infections. This aligns with other research suggesting that adaptive SARS-CoV-2 evolution frequently occurs in individuals with immunosuppression, particularly those with HIV or cancer^1,51–56^. In the case of HIV, these findings underscore the critical importance of initiating antiretroviral therapy to restore CD4+ T cell responses, which are essential for neutralizing antibody activity and the clearance of SARS-CoV-2 infection^51,52^. While the increased risk associated with autoimmune disorders attenuated when adjusting for all other diagnostic groups, we cannot rule out an association, which could plausibly be due to the use of immunosuppressant medications in these individuals, inhibiting their ability to clear the virus.

Notably, viral genomes from immunosuppressed individuals accumulated significantly more nonsynonymous changes during the course of infection, an association that was not observed for other clinical diagnoses. Many of these recurrent nonsynonymous changes have previously been linked to resistance against monoclonal antibody^8,11,13–15,17,35–37^ and antiviral treatments, including nirmatrelvir/ritonavir^38^, remdesivir ^39–41^ and molnupiravir^42,43^. We further found that recurrent mutations were associated with higher between-host fitness than both single-occurrence mutations and alternative amino acid substitutions at the same sites, based on independently derived global phylogenetic estimates. This suggests that recurrent mutations not only arise repeatedly within immunosuppressed hosts but also have a greater potential to spread between individuals, highlighting their importance in ongoing viral evolution and transmission dynamics. Taken together, this highlights the need to vaccinate individuals with immunosuppression and take measures to limit onward transmission until viral shedding has been conclusively resolved^53^.

However, due to limited data on medication administration, we cannot definitively determine whether this adaptive evolution is driven by compromised immune systems that allow for tolerance of novel viral trajectories or increased selection pressure from more aggressive treatment strategies. Supporting a role for treatment-induced selection, most individuals with monoclonal antibody resistance-linked substitutions had an immunosuppression diagnosis, further reinforced by the similar numbers of synonymous changes. Moreover, local clinical guidelines recommended antiviral treatment for high-risk patients, including those with immunosuppression^57^. Nevertheless, among those with infections ending prior to mid-2022, less than sixty percent of these cases involved hospitalized individuals, suggesting that observed viral evolution is not solely attributable to in-hospital treatment. Furthermore, we found no evidence of differing rebound rates between individuals with immunosuppression and without immunosuppression, suggesting that immunosuppression alone is unlikely to drive the emergence of adaptive drug-resistant mutations that could facilitate infection rebound. Further evidence is therefore required to disentangle the extent to which these dynamics in individuals with immunosuppression are due to specific treatment regimes or tolerance due to compromised immune systems.

Our analysis also revealed notable patterns of selection pressure across the SARS-CoV-2 genome. While we found no evidence of genome-wide positive selection, we identified significant selective forces acting on specific coding regions, most notably the spike (S) gene. Signals in ORF7a and E were also observed, but were based on limited data and should be interpreted with caution. ORF7a is involved in immune modulation and antigen presentation interference^58–60^, while the E protein plays roles in virus assembly and host cell interaction that can affect viral replication and pathogenesis^61–63^, which may explain these signals. These selective forces were not evident in acute infections, likely due to their shorter duration and the limited opportunity for within-host viral evolution. However, we detected purifying selection in acute infections, particularly in the ORF1ab region. These findings indicate that adaptive evolution in certain genomic regions may be shaped and constrained by selective pressures imposed by the host immune response and antiviral treatments. While selection pressures are expected in the spike gene, given its central role in viral entry and antibody targeting, signals in other regions may reflect localized adaptation or stochastic variation, given the sample size, and require further validation in larger datasets.

Importantly, the low genomic distances between sequences from persistent infections and those circulating at the same time suggest that onward transmission from persistent SARS-CoV-2 infections remains plausible, though we acknowledge that genetic similarity in a high-prevalence setting is not definitive evidence of direct transmission. Furthermore, the longer phylogenetic tip lengths observed in persistently infected individuals suggest greater within-host molecular change, which may affect the likelihood of onward spread either by enhancing transmission fitness or, alternatively, by reducing transmission fitness through adaptation to the host environment. Comparisons of exact matches^64,65^, which leverage clusters of identical sequences to infer transmission patterns, further highlight the role of persistent infections in both generating new variants and potentially seeding their spread.

Although irregular testing intervals prevented us from estimating population-wide rates of persistent infections, their prevalence is likely higher than previously thought. Conservative estimates from the United Kingdom suggest that 0.1–0.5% of infections may become persistent^8^, underscoring their potential as sources of new variants. Given the strict criteria used to define persistent infections in this study and the non-random testing strategy, the number of cases identified likely represents a significant underestimate of the true burden in the Danish population. Nearly one in five individuals in our cohort experienced viral rebound during their persistent infection. Some persistent infections included intervening negative RT-PCR tests between positive results, which could, in principle, reflect reinfection rather than true persistence. However, our requirement that consecutive sequences share rare SNPs substantially reduces this risk, as independently acquired infections would not be expected to carry the same rare polymorphisms. Cases lacking shared rare SNPs across samples were excluded, thereby filtering out likely reinfections. These dynamic patterns in viral load, as others have noted, further suggest that population-level reinfection rates may be overestimated when defined solely by the presence of a negative test^8^; this highlights the challenges of tracking potentially novel mutations arising from these infections, particularly given their frequency and duration.

This study has several limitations. First, unmeasured confounding factors could obscure the relationship between sociodemographic factors and the risk of persistent infection. In addition, the absence of medication data prevents us from establishing causal links between treatment and the emergence of specific nonsynonymous changes. Second, our analyses were also based on consensus-level sequences, which do not capture sub-consensus variation; as a result, we may have missed important within-host viral diversity^19,66^, particularly at low frequencies. In addition, the inclusion of samples with Ct values up to 38 reflects national sequencing practices in Denmark during the pandemic, though this may include samples with lower viral RNA concentrations than would be retained under more restrictive thresholds. While Ct thresholds for sequencing varied over time in response to national capacity, most variants co-circulated during the period of highest sequencing intensity, making systematic bias between variants unlikely. Moreover, our inference of persistent infection is based on genetic continuity within a single viral lineage rather than viral load alone. Although cases failing sequencing quality control may reduce sensitivity for detecting persistence, our reliance on shared rare SNPs mitigates the risk of spurious inference.

Third, our findings may primarily reflect individuals who undergo frequent testing, potentially due to specific occupations, socioeconomic factors, or hospitalization, which leads to more frequent testing. In particular, older adults could be more likely to undergo repeated testing when persistent infection is clinically expected (e.g., due to frailty, symptoms, or hospitalization), whereas younger adults may be tested primarily for logistical reasons, such as returning to work or normal activities. This could exaggerate the apparent relationship between age and persistence risk, even in the absence of a true biological effect. This is particularly relevant for infections occurring from 2022 onwards, when systematic community testing was scaled back. As a result, the study population may not accurately represent the true distribution of persistent infections in the broader population. The higher prevalence in older age groups may also reflect prolonged PCR positivity due to delayed clearance of viral RNA rather than ongoing active viral replication. Moreover, conditioning on survivors could induce collider-stratification bias for any diagnosis or factor that affects mortality and shares unmeasured causes with persistent infection. Finally, while the case-control design was well-suited for studying the rare outcome of persistent infections, it may still be subject to selection biases that could affect the robustness of the results. To mitigate this, we employed a matching procedure that ensured comparability between cases and controls in terms of both testing behaviors and variants.

Overall, our study highlights the role of age and health conditions, particularly immunosuppression, as risk factors for persistent SARS-CoV-2 infections and contributors to viral mutation during such infections. These results emphasize the importance of closely monitoring the evolutionary trajectories of persistently infected individuals, especially those who are immunosuppressed and receiving ongoing treatment, to track the emergence of potentially novel variants.

## Methods

### Testing, genomic data and sequencing in Denmark

This study was conducted using data from national registers and previously generated SARS-CoV-2 consensus genome sequences only. According to Danish law, ethics approval is not needed for this type of research, and the requirement for individual informed consent was waived accordingly. All data management and analyses were carried out on Statistics Denmark’s secure research servers. Large-scale sequencing of SARS-CoV-2 was a key strategy in response to COVID-19 in Denmark. The Danish COVID-19 Genome Consortium (DCGC) - formed in March 2020 - undertook sequencing efforts to monitor the evolution of SARS-CoV-2. The DCGC included all genomic sequences that met the inclusion criteria based on a cycle threshold (Ct) value, which ranged from 30 to 38 during the study period. Whole genome sequencing (WGS) was conducted by multiple institutions, including Aalborg University (AAU), *Statens Serum Institut* (SSI), and regional clinical microbiology laboratories. Sequencing workflows commonly employed whole genome amplification using a modified version of the ARTIC tiled PCR approach^67^ (https://artic.network), generating overlapping amplicons ranging from approximately 1000 to 1500 base pairs in length. A custom 2-step PCR method facilitated the barcoding of amplicon libraries, which were then normalized, pooled, and prepared for sequencing using Oxford Nanopore’s SQK-LSK109 ligation kit. Sequencing was primarily performed on the MinION platform with Oxford Nanopore R.9.4.1 flow cells^68^. Raw data was base-called using Guppy (https://nanoporetech.com) and demultiplexed via a custom cutadapt v.2.10 wrapper^69^. Consensus sequences were generated with the artic minion function, following ARTIC network protocol defaults (v.1.1.0) and incorporating medaka^70^ for consensus calling.

Additional sequencing workflows utilized the ARTIC v3 amplicon sequencing panel^71^, which consisted of 98 overlapping amplicons of approximately 300 nucleotides, sometimes including custom spike-ins to maintain consistent coverage^72^. This sequencing approach was executed on Illumina platforms, such as the NextSeq or NovaSeq, with paired read lengths ranging from 51 to 150 nucleotides (most often 74 nucleotides). Sequencing reads underwent trimming with trim-galore v.0.6.10 (https://github.com/FelixKrueger/TrimGalore), with consensus sequences generated using iVar^73^ (v.1.4.3) or a combination of iVar and custom BCFtools v.1.18 commands. A PHRED score threshold of 20 was applied during read and primer trimming. Sequences from regional labs employing either Nanopore or Illumina sequencing were also integrated into national sequence databases, provided they met quality criteria: fewer than 3000 ambiguous bases (N’s), ≤ 5 ambiguous base calls, high yield relative to controls, and passing QC standards that detected contamination. Sequence integrity was verified against SARS-CoV-2 reference models, with tools such as VADR^74^, prior to being uploaded to sequence data repositories. Metadata related to sequencing included information on sampling and sequencing dates. The sequences were aligned to the Wuhan WIV04 (MN996528.1) reference genome^75^ using MAFFT (v.7.520)^76^. Following the alignment, problematic regions identified by De Maio et al.^77^ were masked using the *augur mask* tool from the *Nextstrain* pipeline^78^ (Augur version v.22.3.0). This masking process included regions at both the 5’ end (positions 1–55) and the 3’ end (from site 28804 onwards).

### Defining and identifying persistent infections

To identify possible persistent infections, we adopted the method used by Ghafari et al.^8^ In short, possible persistent infections were defined as infections spanning 26 days or more (i.e., two or more positive tests within the same major variant, ≥ 26 days and  < 120 days apart, identified using Pangolin v4.3.1). The original justification for the 26 day cut-off by Ghafari et al.^8^ was based on the observation that most individuals with acute infection shed the virus for less than 20 days and no longer than 30 days in the respiratory tract^79,80^. Infections were defined as all positive tests within this period, from the first to the last positive test for a given variant. Only infections that began before January 1, 2023, were included to ensure that vaccination data were available for all individuals.

In addition, the primary lineages (and respective sub-lineages) we analyzed included Alpha (B.1.1.7), Delta, Omicron BA.1, Omicron BA.2, and Omicron BA.5. We assessed whether all sequences from each individual-level infection were from the same infection by checking for shared rare single-nucleotide polymorphisms (SNPs) at consecutive time points relative to the population for the given major variant. SNPs were classified as rare if their frequency in the population-level dataset fell below a specified threshold. To establish a threshold that maximized the identification of persistent infections while minimizing false positives, we generated a dataset of 1000 randomly paired sequences from different individuals with the same major variant in the population dataset, ensuring they were sampled 26 days apart. We then assessed the proportion of incorrectly identified persistent infections at different thresholds for defining rare SNPs. Although raising the threshold resulted in a higher number of identified persistent infections, it also increased the false-positive rate among the randomly paired sequences at excessively high thresholds. Given the strict definitions for persistent infections, we expect the number of identified persistent infections to be an under-estimate of the true number. We generated 100 different thresholds using logarithmic spacing (Supplementary Fig. 4). Among these, the threshold of 10.9% (i.e., the frequency of the given SNP is below the threshold among all other population-level sequences within the same major variant) was chosen to maximize the detection of persistent candidates while constraining the false positive rate (see dotted vertical lines in Supplementary Fig. 4). This resulted in false-positive rates of 3.1% (Alpha), 3.0% (Delta), 3.3% (Omicron BA.1), 2.1% (Omicron BA.2), and 2.2% (Omicron BA.5).

### Registry linkage

To associate sequences with Danish registry data, pseudo-anonymized Danish civil registration numbers (CPR numbers) were employed to connect sequences to the relevant registry information maintained by Statistics Denmark. This registry data included sex, age, test date, and vaccination status. The following vaccinations were administered as part of the Danish vaccination program: Comirnaty (Pfizer/BioNTech), Spikevax (Moderna), Vaxzevria (AstraZeneca), and COVID-19 Vaccine Janssen (Johnson & Johnson)^29,81^. Individuals were considered vaccinated at the time of infection onset if they had received one, two, or three doses, with at least seven days having passed since their most recent dose.

For diagnostic codes to identify health conditions, we linked CPR numbers to the National Patient Register^82^ to obtain relevant in- and out-patient ICD-10 codes for immunosuppression, autoimmune disorders, Immune-Mediated Inflammatory Diseases (IMID) and diabetes using previously defined codelists. A full list of ICD-10 codes by health condition can be found in Supplementary Tables 21–24. The National Patient Register includes data on individuals who have been admitted to somatic hospital departments since 1977 and ambulatory care from 1995. Individuals were considered part of a disease group (i.e., binary) if they had an ICD-10 code in the relevant disease group. Charlson Comorbidity Index (CCI)^31^ scores were calculated using the heaven package^83^, with the relevant ICD-10 codes and weightings defined by the Danish Multidisciplinary Cancer Group (DMCG)^84,85^. CCI values were computed using both 5-year and 10-year look-back periods from the start of the infection. Hospitalization was defined as an admission lasting 12 hours or longer.

### Case-control design

To identify risk factors for persistent infections, we designed a case-control study in which individuals with a persistent infection served as cases, and other individuals within the Danish testing database were considered potential controls. Controls were identified by matching each case to individuals with a positive PCR result for the same major variant, within  ± 1 day of the case’s infection start date. This matching was crucial to ensure comparability between cases and controls in terms of testing behaviors as well as variants. Controls were defined as individuals with a negative PCR test within 26 days and no positive tests during the 120 days following their positive test date. The 120-day window was chosen to exceed the standard 90-day reinfection threshold, providing a conservative buffer to ensure definitive viral clearance and minimize misclassification of rebounds or new infections.

To investigate risk factors for persistent infections, we used Bayesian multivariable conditional logistic regression^30^, accounting for the matched design by including strata corresponding to each matched case-control set (comprising one case and their controls). We developed five key models: baseline, immunosuppression, autoimmune disorders, IMID, and diabetes. A weakly informative prior ${{{\mathcal{N}}}}(0,1)$ was placed on all regression coefficients; the model was fit using four Markov chains with 2000 iterations each, including a warm-up phase of 1000 iterations. Posterior summaries were generated, including point estimates and 95% credible intervals based on the 2.5th and 97.5th percentiles of the posterior distributions. Models were run using brms^86^ (v.2.20.3). The baseline model included covariates age group (0–15 [0 ≤ age < 15], 15–30 [15 ≤ age < 30], 30–45 [30 ≤ age < 45], 45–60 [45 ≤ age < 60], 60–75 [60 ≤ age < 75], 75 + [age ≥7 5]), sex (female, male), vaccination status (which combines the number of vaccinations at infection onset and the time since the last vaccination  < 18 weeks or ≥ 18 weeks) for those with two or three vaccinations), and the Charlson Comorbidity Index (CCI). The 18-week cut-off was chosen to account for immunity, which typically wanes between three and four months post-vaccination, followed by a stabilization period^87–89^. Two and three vaccinations were grouped due to policy changes during the pandemic, during which both were considered fully vaccinated at different stages.

In the models for immunosuppression, autoimmune disorders, IMID, and diabetes, the same covariates were used, but CCI was replaced by the relevant condition for each model. Major variant was excluded in the models due to matching individuals by test date and major variant. For baseline models, we used pre-pandemic diagnoses (i.e., those prior to 2020-01-01) to clarify the temporal relationship between vaccinations and relevant diagnoses, including CCI values. Sensitivity analyses examined the impact of using contemporary diagnoses (i.e., those registered up to 2021-12-31, for which diagnostic data is available). Sensitivity analyses further explored interactions between covariates to understand their combined influence on persistent infections. CCI values were also tested using both 5-year and 10-year look-back periods from the start of infection.

### Characterizing mutations and viral load trajectories

For consecutive within-host consensus sequences, we identified mutation sites using custom code in R version 4.2.3, which compared aligned sequences pairwise to detect nucleotide differences at each position. To assess changes in viral RNA load during persistent infections, we compared Ct values from the initial sequence collection to those at the final available sequencing time point. The distribution of Ct differences was approximately normal (Shapiro-Wilk test [*W* = 0.976, *p* = 0.192])^90^. We modeled the difference in Ct values using a Bayesian approach implemented in brms, specifying a weakly informative normal prior, ${{{\mathcal{N}}}}(0,1)$, on the mean Ct difference.

To assess the between-host fitness effects of individual amino acid substitutions, we used a model developed by Bloom et al.^34^, which estimates the fitness effects of specific amino acid changes based on a global phylogeny. These fitness effects are inferred by comparing observed versus expected mutation counts. Data for specific amino acid changes is available here: https://github.com/jbloomlab/SARS2-mut-fitness/blob/main/results/aa_fitness/aa_fitness.csv. We then compared (a) the fitness of recurrent mutations, defined as those observed in two or more persistently infected individuals, with the fitness of single-occurrence mutations (those observed only once) across all persistent infections, and (b) the fitness of recurrent mutations against other amino acids observed at the same sites (i.e., comparing each recurrent mutation to other amino acids not observed at the same position). We constructed a Bayesian linear model to evaluate fitness differences between recurrent mutations and their respective comparison groups, specifying a weakly informative prior (${{{\mathcal{N}}}}(0,1)$) for the regression coefficients, with model fit using brms.

To compare tip lengths between persistent and non-persistent infections, we constructed a phylogenetic tree for each set of cases and controls (from the conditional logistic regression analysis) with at least 10 controls (*N *= 293). For each case-control set, we performed phylogenetic analysis using IQ-TREE (v1.6.12) with a GTR + F substitution model. The Wuhan WIV04 (MN996528.1) reference sequence^75^ was used as the outgroup.

For Ka and Ks difference calculations, we used the kaks function from the seqinr package (v4.2.23), analyzing sequences from both persistent and non-persistent infections. For persistent infections, we selected the first and last sequence from each infection episode. For non-persistent infections, sequences from control individuals identified through conditional logistic regression analysis were used. Individuals with two or more sequences (starting from their matched infection date) were included, and we analyzed the first and last sequence within the infection episode, defined as sequences collected within 26 days of the infection start date. Alignments were split by coding region to calculate region-specific Ka and Ks values. To compare the differences in Ka and Ks values, we assumed a normal distribution and conducted a Bayesian analysis. The model was implemented using the brms package, with a weakly informative prior (${{{\mathcal{N}}}}(0,0.01)$) specified for the intercept. Model fitting was performed using four chains, each with 2000 iterations, including a warm-up phase of 1000 iterations.

### Characteristics associated with number of (non)synonymous mutations

To investigate factors associated with the number of synonymous and nonsynonymous mutations per infection, we analyzed the initial and final viral sequences from each case, calculating the number of synonymous and nonsynonymous substitutions over the infection period. We used a zero-inflated negative binomial regression model to account for excess zeros and overdispersion, regressing mutation counts on host characteristics, including age and major variant, while adjusting for the time interval between sample dates. Variable selection was performed using stepwise AIC with the *MASS*^91^ package (v7.3.58.2) in *R*.

We further examined the influence of specific diagnoses, such as immunosuppression, autoimmune disorders, IMID, and diabetes, by incorporating these conditions as covariates in separate models; in these, we adjusted for age, sampling interval, and major variant. Sensitivity analyses were conducted using pre-pandemic diagnoses (registered before 2020) for both individual diagnosis models and for CCI scoring in the baseline model. To identify episodes of viral rebound, we used the national SARS-CoV-2 testing database to find negative test results during the individual’s infection. For Bayesian logistic regression models comparing the likelihood of rebounding, a weakly informative prior ${{{\mathcal{N}}}}(0,1)$ was placed on regression coefficients. The model was fit using four Markov chains with 2000 iterations each (1000 warm-up), implemented in brms.

### Reporting summary

Further information on research design is available in the Nature Portfolio Reporting Summary linked to this article.

## Supplementary information

Supplementary Information

Peer Review File

Reporting Summary

## Data Availability

The SARS-CoV-2 consensus genome data used in this study have been deposited in the GISAID database as part of the Danish SARS-CoV-2 surveillance response (EPI-SET accession number EPI_SET_260124ub). The linked individual-level clinical, demographic, and genomic data are available under restricted access due to Danish data protection legislation, as linkage of genomic identifiers to sensitive clinical metadata could allow re-identification of individuals. Access can be obtained by submitting a formal application to the Danish Health Data Authority (https://english.sundhedsdatastyrelsen.dk/health-data-and-registers/research-services/apply-for-data) and Statens Serum Institut. Access is granted to researchers affiliated with authorized institutions for purposes consistent with the original ethical approval, subject to regulatory approval and a formal data processing agreement. The Danish Health Data Authority typically processes access requests within 6 months, and data remains available for the duration specified in the approved agreement. A list of specific GISAID accession numbers is not provided in this manuscript, as linking these identifiers to the clinical metadata analyzed here could enable re-identification, which is prohibited under Danish law; the corresponding sequence identifiers can be provided as part of an approved data access request. The raw individual-level registry data are protected and are not available due to data privacy laws. No data collection or sequencing was conducted specifically for this study. Synthetic data to demonstrate the code functionality are available at https://github.com/MLGlobalHealth/sars-cov2-persistent.

## Code availability

All relevant code is available on GitHub (https://github.com/MLGlobalHealth/sars-cov2-persistent) and Zenodo (10.5281/zenodo.16359338).
