# Supplementary material for: Large-scale genomic surveillance reveals immunosuppression drives mutation dynamics in persistent SARS-CoV-2 infections
Source: Nat Commun. 2026 Jun 19;17:7730. doi: 10.1038/s41467-026-74445-7 (PMC13433919; doi:10.1038/s41467-026-74445-7)
Supplement: Supplementary file 1 [file 41467_2026_74445_MOESM1_ESM.pdf]

Large-scale genomic surveillance reveals immunosuppression drives mutation dynamics in persistent SARS-CoV-2 infections

Supplementary File

Appendix A Supplementary Figures

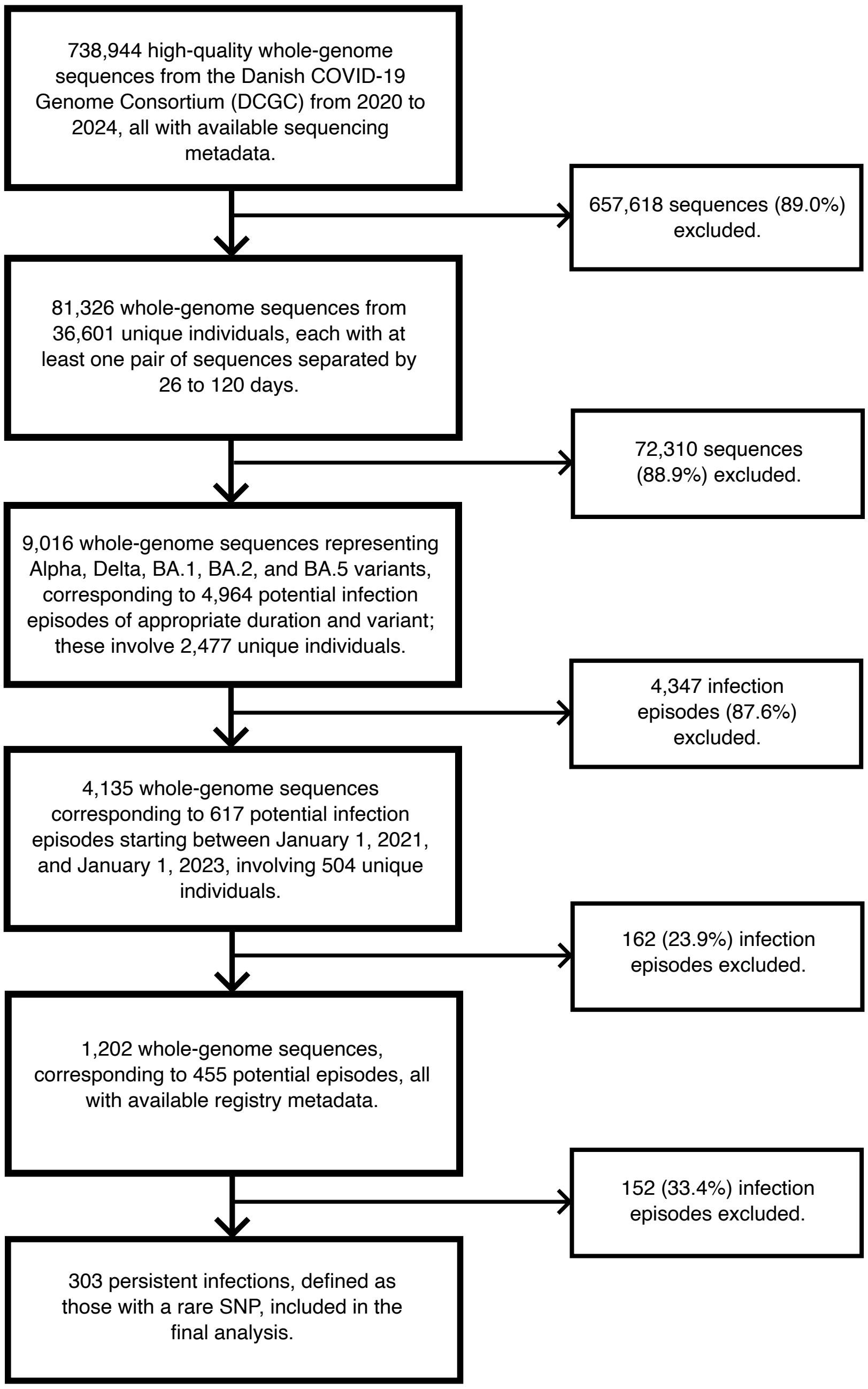

Supplementary Figure 1 Flowchart for finding relevant sequences and defining persistent infections in this study.

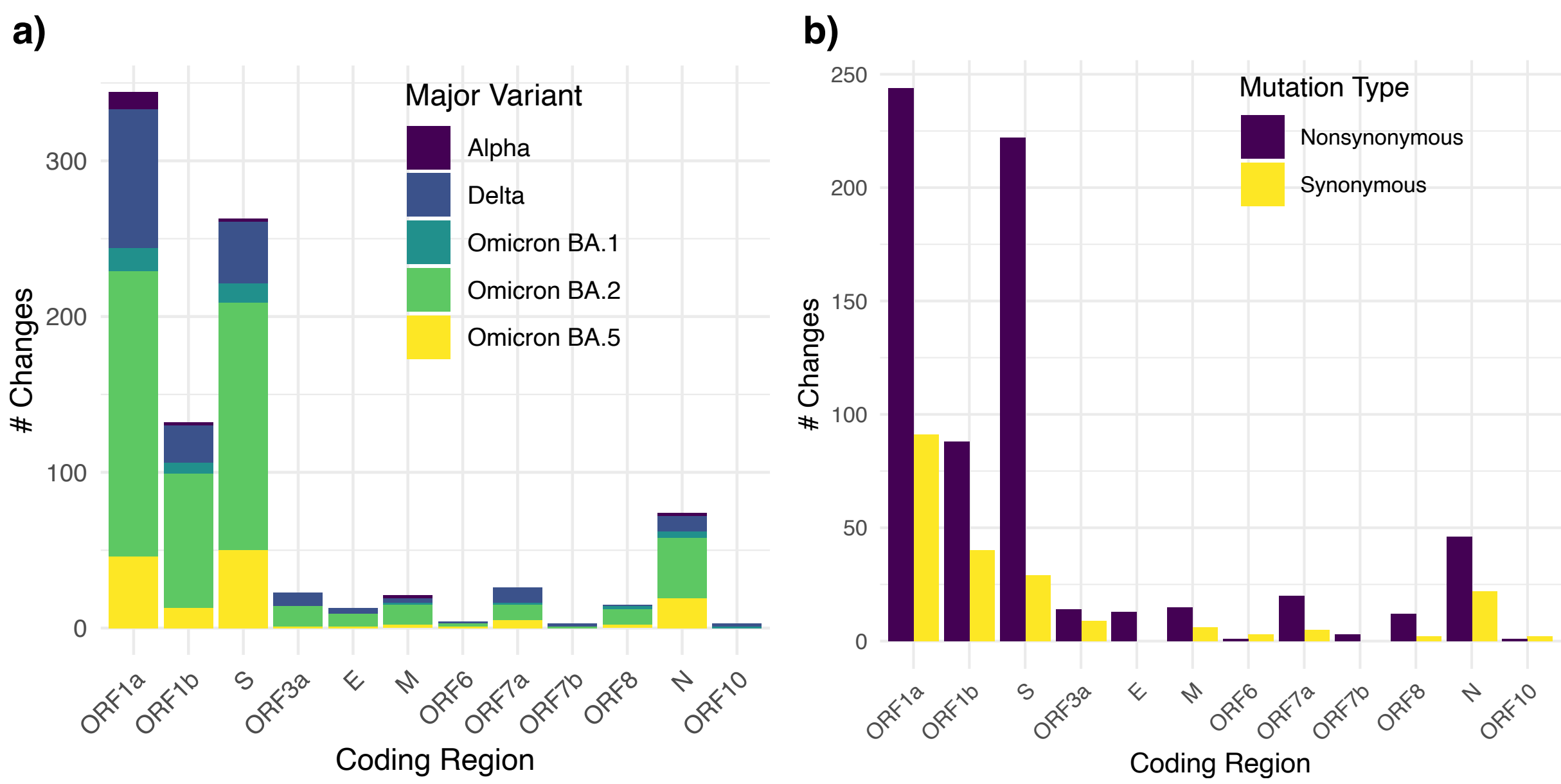

Supplementary Figure 2 a) Number of mutations and b) (non)synonymous changes by coding region across all pairs of consecutive sequences.

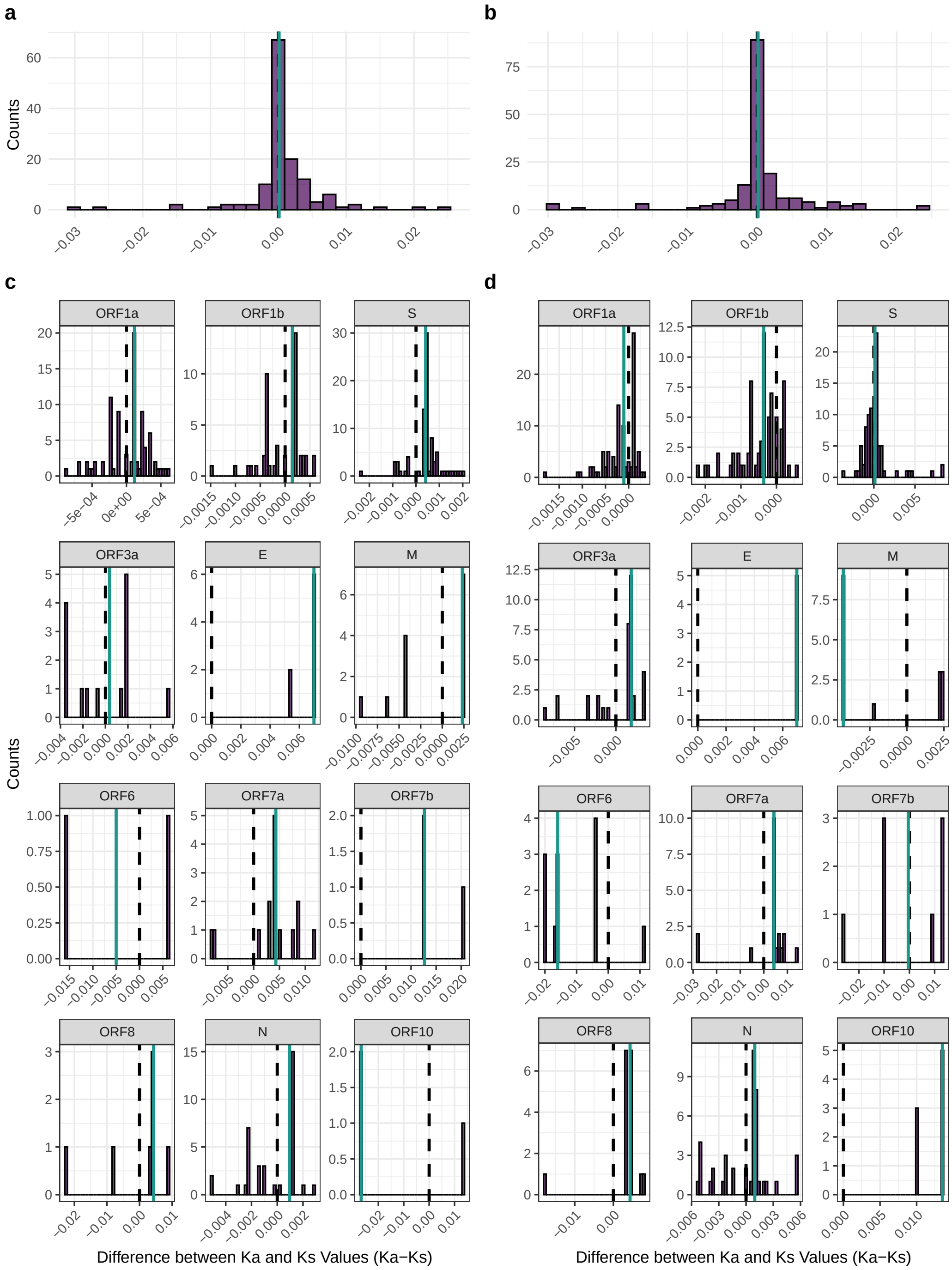

Supplementary Figure 3 Differences in Ka and Ks values (Ka - Ks) across the genome and by coding region. (a) Difference in Ka and Ks values among individuals with persistent infections ($N = 135$). Ka and Ks represent the substitution rates per non-synonymous and synonymous site, respectively. The dotted line indicates a value of 0, and the green line represents the median. (b) Difference in Ka and Ks values among individuals without persistent infections (controls, $N = 167$) with two or more available sequences. (c) Difference in Ka and Ks values in each coding region among individuals with persistent infections, specifically those with at least one mutation in the region. (d) Difference in Ka and Ks values in each coding region among individuals without persistent infections, specifically those with at least one mutation in the region.

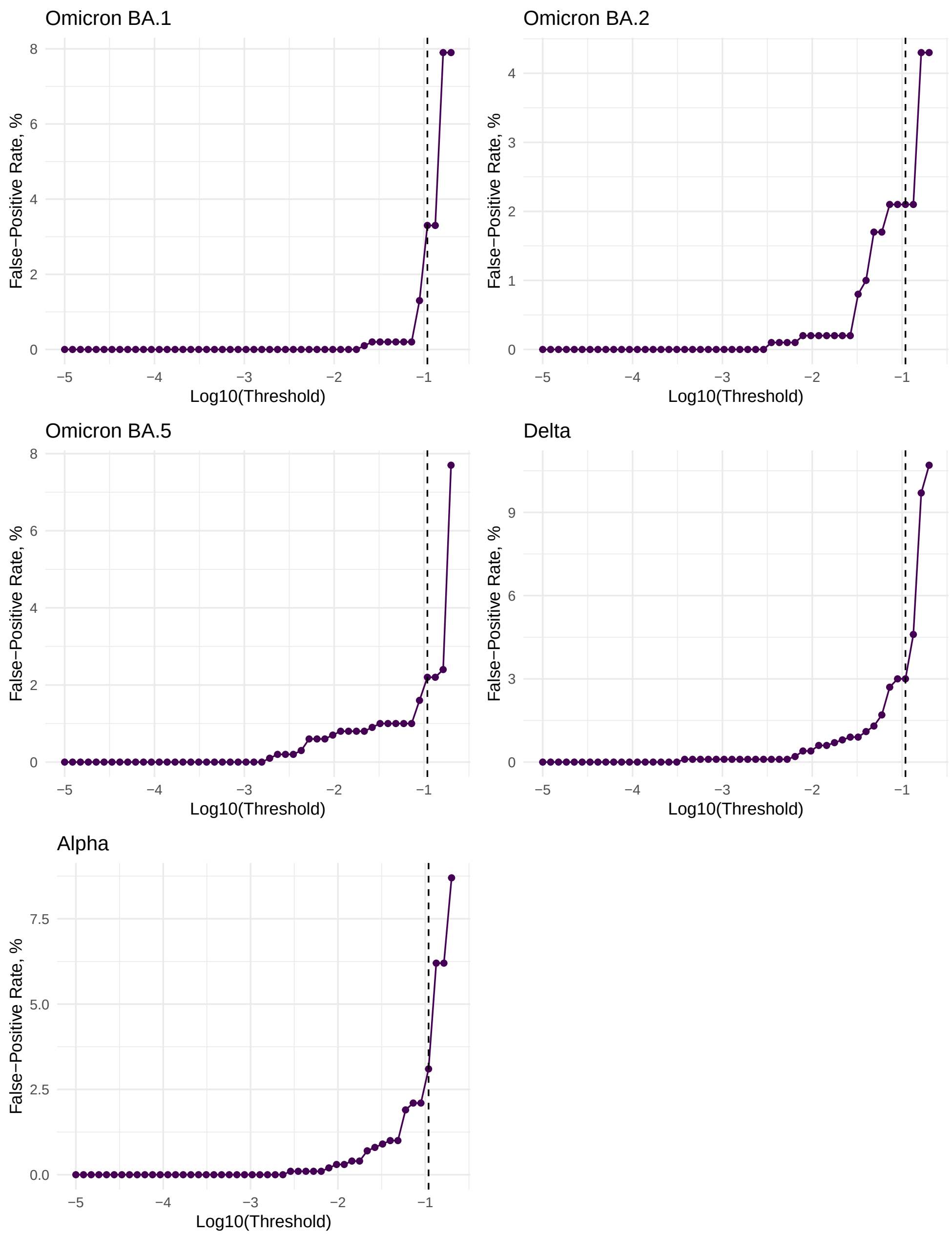

Supplementary Figure 4 Plots with false-positive rates at different thresholds for each major variant. We chose a threshold of 10.9 percent (dashed line) to identify persistent infections, since for this threshold the percentage of false positives were 2-3% for all major lineages.

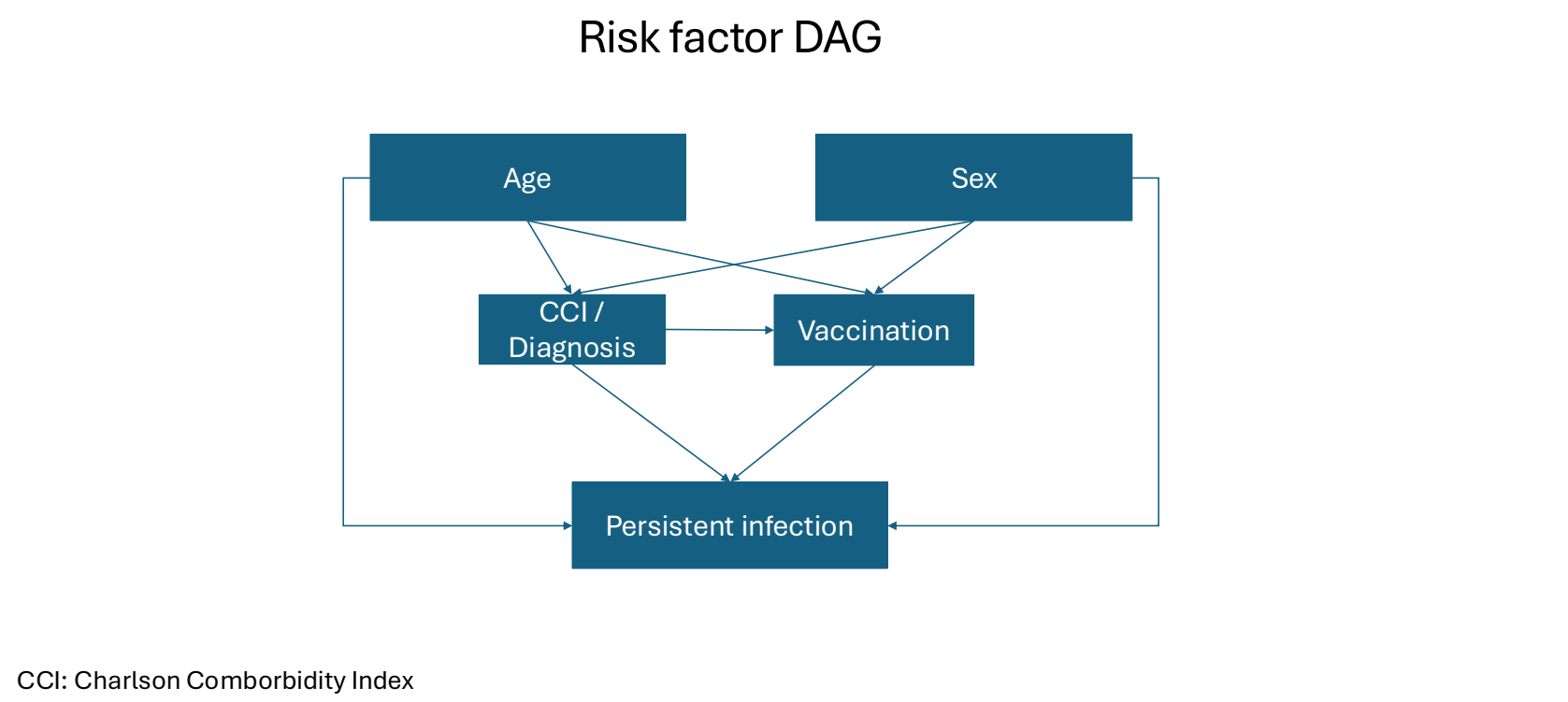

Supplementary Figure 5 DAG for relationship between covariates and persistent infection.

Appendix B Supplementary Tables

Supplementary Table 1 Distribution of Variants, Age Groups, Sex, and Number of Vaccinations (n=303)

Major Variant	Count (Percentage)
Alpha	42 (13.9%)
Delta	63 (20.8%)
Omicron BA.1	27 (8.9%)
Omicron BA.2	132 (43.6%)
Omicron BA.5	39 (12.9%)

Age Group	Count (Percentage)
0-15	10 (3.3%)
15-30	24 (7.9%)
30-45	31 (10.2%)
45-60	45 (14.9%)
60-75	98 (32.3%)
75+	95 (31.3%)

Sex	Count (Percentage)
Male	194 (64.0%)
Female	109 (36.0%)

Number of vaccinations at infection	Count (Percentage)
0	76 (25.1%)
1	8 (2.6%)
2	44 (14.5%)
3	175 (57.8%)

**Variable**		**Cases (Median [IQR])**	**Controls (Median [IQR])**	**Adjusted* OR (95% CrI)**
Vaccination status	No vaccination	76	56497	Ref.
	1 Vaccination	8	6520	0.61 (0.29-1.22)
	2 or 3 Vaccinations, ¡18 weeks	90	52046	0.54 (0.35-0.83)
	2 or 3 Vaccinations, $\geq$ 18 weeks	129	31841	0.80 (0.53-1.24)
**Charlson Comorbidity Index†**		1 [0-2]	0 [0-0]	1.29 (1.22-1.37) Per 1 Increase
*The Charlson Comorbidity Index model was adjusted for age and sex, and the vaccination model was adjusted for age, sex, and Charlson Comorbidity Index; CrI = Credible Interval				
† 10-year lookback from 2020-01-01				

Supplementary Table 2 Risk factors for persistent infection. Age and sex as in the main table, as the Charlson Comorbidity Index was not included in their model. Values are presented as median [IQR] for continuous variables and n (%) for categorical variables. Adjusted odds ratios (OR) with 95% credible intervals (CrI) are provided from the conditional logistic regression output. CrI = credible interval. The Charlson Comorbidity Index was calculated with a 10-year lookback period from January 1, 2020. Vaccination status combines the number of vaccinations at infection onset and the time since the last vaccination ($<$ 18 weeks or $\geq$ 18 weeks) for those with 2 or 3 vaccinations. The total number of controls is n = 146,904, corresponding to 62,648 unique individuals.

**Variable**		**Cases (Median [IQR])**	**Controls (Median [IQR])**	**Adjusted* OR (95% CrI)**
Vaccination status	No vaccination	76	56497	Ref.
	1 Vaccination	8	6520	0.66 (0.30-1.26)
	2 or 3 Vaccinations, ¡18 weeks	90	52046	0.53 (0.35-0.82)
	2 or 3 Vaccinations, $\geq$ 18 weeks	129	31841	0.76 (0.50-1.17)
**Charlson Comorbidity Index†**		1 [0-2]	0 [0-0]	1.43 (1.35-1.52) Per 1 Increase

*The Charlson Comorbidity Index model was adjusted for age and sex, and the vaccination model was adjusted for age, sex, and Charlson Comorbidity Index; CrI = Credible Interval.

† 5-year lookback from date of infection

Supplementary Table 3 Risk factors for persistent infection. Age and sex as in the main table, as the Charlson Comorbidity Index was not included in their model. Values are presented as median [IQR] for continuous variables and n (%) for categorical variables. Adjusted odds ratios (OR) with 95% credible intervals (CrI) are presented from the conditional logistic regression output. CrI = credible interval. The Charlson Comorbidity Index was calculated with a 5-year lookback period from the date of infection. Vaccination status combines the number of vaccinations at infection onset and the time since the last vaccination ($<$ 18 weeks or $\geq$ 18 weeks) for those with 2 or 3 vaccinations. The total number of controls is $n = 146{,}904$, corresponding to 62,648 unique individuals.

Diagnosis Group†		Cases	Controls	Adjusted* OR (95% CrI)
**Immunosuppression**	Yes	76	1929	5.77 (4.63-7.12)
	No	227	144975	Ref.
**Autoimmune**	Yes	13	1611	1.42 (0.89-2.10)
	No	290	145293	Ref.
**Diabetes**	Yes	28	4717	1.00 (0.74-1.30)
	No	275	142187	Ref.
**IMID**	Yes	18	4775	1.08 (0.74-1.49)
	No	285	142129	Ref.

*Apart from adjusting for all other diagnoses, the model is also adjusted for sex, age, and vaccination status; CrI = Credible Interval.

† Using pre-pandemic diagnoses (i.e., those prior to 2020-01-01).

Supplementary Table 4 Diagnoses associated with persistent infection. Values are presented as n for categorical variables. Adjusted odds ratios (OR) with 95% credible intervals (CrI) are presented from the conditional logistic regression output. CrI = credible interval; IMID = immune-mediated inflammatory diseases. Diagnoses are based on pre-pandemic conditions (i.e., those prior to January 1, 2020). Vaccination status combines the number of vaccinations at infection onset and the time since the last vaccination (< 18 weeks or ≥ 18 weeks) for those with 2 or 3 vaccinations. The total number of controls is $n = 146{,}904$, corresponding to $n = 62{,}648$ unique individuals.

Variable		# Cases	# Controls	Unadjusted OR (95% CI)	Adjusted* OR (95% CI)
**Model 1: Immunosuppression Diagnosis**	Yes	117	2477	10.65 (8.90-12.78)	7.85 (6.56-9.43)
	No	186	144427	Ref.	Ref.
**Model 2: Autoimmune Diagnosis**	Yes	16	1825	2.34 (1.60-3.35)	1.70 (1.14-2.39)
	No	287	145079	Ref.	Ref.
**Model 3: Diabetes Diagnosis**	Yes	30	4959	1.87 (1.42-2.45)	1.13 (0.84-1.49)
	No	273	141945	Ref.	Ref.
**Model 4: IMID Diagnosis**	Yes	19	5110	1.36 (0.95-1.88)	1.13 (0.80-1.56)
	No	284	141794	Ref.	Ref.
*All adjusted for sex and age.					

Supplementary Table 5 Diagnoses associated with persistent infection. Unadjusted and adjusted odds ratios (OR) with 95% confidence intervals (CI) are provided, with adjustments for sex, age, and number of vaccinations. IMID = Immune-mediated inflammatory diseases. The diagnoses were recorded up to December 31, 2021, for which diagnostic data is available. The total number of controls is $n = 146{,}904$, corresponding to 62,648 unique individuals.

Supplementary Table 6 Exact (0-difference) sequence matches between persistent cases and database controls, stratified by Danish region.

Region (Denmark)	Persistent cases with ≥ 1 exact match	Exact matches (0 differences)
Northern Jutland	0	0
Central Jutland	0	0
Southern Denmark	3	23
Capital Region	3	3
Zealand	4	7
**Total**	**10**	**33**

Coding Region	Estimate	Lower 95% CrI	Upper 95% CrI
ORF1a	2.78×10^{-5}	-3.68×10^{-5}	9.25×10^{-5}
ORF1b	-6.91×10^{-5}	-1.87×10^{-4}	5.14×10^{-5}
S	4.12×10^{-4}	2.66×10^{-4}	5.56×10^{-4}
ORF3a	-1.60×10^{-4}	-1.88×10^{-3}	1.59×10^{-3}
E	6.59×10^{-3}	5.87×10^{-3}	7.28×10^{-3}
M	-1.28×10^{-3}	-3.96×10^{-3}	1.26×10^{-3}
ORF6	-7.78×10^{-4}	-1.75×10^{-2}	1.74×10^{-2}
ORF7a	3.58×10^{-3}	5.27×10^{-4}	6.60×10^{-3}
ORF7b	9.98×10^{-3}	-8.25×10^{-3}	2.04×10^{-2}
ORF8	-6.72×10^{-4}	-1.01×10^{-2}	8.96×10^{-3}
N	-2.52×10^{-4}	-8.53×10^{-4}	3.62×10^{-4}
ORF10	-3.30×10^{-3}	-2.09×10^{-2}	1.51×10^{-2}

Supplementary Table 7 Summary of Ka-Ks Estimates with Credible Intervals for Persistent Infections

Coding Region	Estimate	Lower 95% CrI	Upper 95% CrI
ORF1a	-1.59×10^{-4}	-2.32×10^{-4}	-8.75×10^{-5}
ORF1b	-4.32×10^{-4}	-5.64×10^{-4}	-3.03×10^{-4}
S	2.43×10^{-4}	-1.79×10^{-4}	6.74×10^{-4}
ORF3a	4.24×10^{-4}	-6.06×10^{-4}	1.48×10^{-3}
E	6.99×10^{-3}	6.99×10^{-3}	6.99×10^{-3}
M	-1.69×10^{-3}	-3.41×10^{-3}	8.14×10^{-5}
ORF6	-9.70×10^{-3}	-1.55×10^{-2}	-3.22×10^{-3}
ORF7a	1.80×10^{-3}	-3.40×10^{-3}	7.14×10^{-3}
ORF7b	-8.21×10^{-4}	-1.13×10^{-2}	9.75×10^{-3}
ORF8	2.86×10^{-3}	-3.03×10^{-4}	5.84×10^{-3}
N	-8.28×10^{-5}	-9.64×10^{-4}	7.77×10^{-4}
ORF10	1.22×10^{-2}	1.05×10^{-2}	1.38×10^{-2}

Supplementary Table 8 Summary of Ka-Ks Estimates with Credible Intervals for Controls

Mutation	Amino acid change	Counts
G22580A	S:E340K (RBD)	15
C11750T	ORF1ab:L3829F	9
C5178T	ORF1a:T1638I	9
A22775G	S:N405D (RBD)	8
G15451A	ORF1ab:G5063S	8
G22599C	S:R346T (RBD)	7
A22893G	S:K444R (RBD)	5
C28550A	N:R93R	5
A22812T	S:N417I (RBD)	4
C26333T	E:T30I	4
G5180A	ORF1ab:D1639N	4
A12759G	ORF1ab:D1323N	3
A22629C	S:K356T (RBD)	3
A22881G	S:K440R (RBD)	3
A23854C	S:K764N	3
C13458T	ORF1ab:S4398L	3
C21588T	S:P9L (NTD)	3
C21846T	S:T95I (NTD)	3
C4878T	ORF1ab:T1538I	3
C7851T	ORF1ab:A2529V	3
G22599T	S:R346I (RBD)	3
G22775A	S:D405N (RBD)	3
G22898A	S:G446S (RBD)	3
G24570T	S:S1003I	3
G5305A	ORF1ab:L1680L	3
G569A	ORF1ab:E102K	3
G9479T	ORF1ab:G3072C	3
T22579C	S:D339D (RBD)	3

Supplementary Table 9 Most common recurrent mutations between first and last sequences in infection episodes. RBD = receptor-binding domain. NTD = N-terminal domain.

Mutation	Amino acid change	Counts
G22580A	S:E340K (RBD)	14
A22775G	S:N405D (RBD)	12
C11750T	ORF1ab:L3829F	8
G15451A	ORF1ab:G5063S	8
C5178T	ORF1a:T1638I	7
G22599C	S:R346T (RBD)	7
A22893G	S:K444R (RBD)	6
C28550A	N:R93R	5
C26333T	E:T30I	5
A22812T	S:N417I (RBD)	4
G5180A	ORF1ab:D1639N	4
C22916A	**S:L452M** (RBD)	4
G15814A	**ORF1ab:V5184I**	4
G22599T	S:R346I (RBD)	4
G22775A	S:D405N (RBD)	4
A23854C	S:K764N	4
C12789T	**ORF1ab:T4175I**	3
C21618T	**S:T19I** (NTD)	3
C25614T	**ORF3a:S74S**	3
C29250T	**N:P326L**	3
C4230T	**ORF1ab:T1322I**	3
G22580C	**S:E340Q** (RBD)	3
G22894T	**S:K444N** (RBD)	3
T21618C	**S:I19T** (NTD)	3
T23042C	**S:S494P** (RBD)	3
A22881G	S:K440R (RBD)	3
C13458T	ORF1ab:S4398L	3
C21588T	S:P9L (NTD)	3
C21846T	S:T95I (NTD)	3
C4878T	ORF1ab:T1538I	3
C7851T	ORF1ab:A2529V	3
G569A	ORF1ab:E102K	3
G9479T	ORF1ab:G3072C	3

Supplementary Table 10 Most common recurrent mutations observed across consecutive sequences, with each infection contributing only once to the counts. Bold mutations are ones that do not appear ≥ 3 times when comparing mutations between first and last sequences directly. RBD = receptor-binding domain. NTD = N-terminal domain.

Mutation	Amino acid change	Treatment	Intro. quarter (DK)	Counts	Quarters observed
G22580A	S:E340K	Sotrovimab	2021 Q3	14	2021 Q4, 2022 Q1, 2022 Q2
C11750T	ORF1ab:L3829F	Nirmatrelvir / Ritonavir	2022 Q4	9	2022 Q2, 2022 Q3, 2022 Q4
G22599C	S:R346T	mABs	NA	7	2022 Q3, 2022 Q4
A22893G	S:K444R	mABs	NA	6	2022 Q3, 2022 Q4
G15814A	ORF1ab:V5184I	Remdesivir	2021 Q1	4	2022 Q1, 2022 Q2, 2022 Q3
G22599T	S:R346I	mABs	NA	4	2022 Q3, 2022 Q4
A22881G	S:K440R	Molnupiravir	2021 Q4	3	2022 Q4, 2023 Q1
C21588T	S:P9L	Molnupiravir	2021 Q4	3	2022 Q2, 2023 Q1
G22580C	S:E340Q	Sotrovimab	2021 Q3	3	2022 Q1, 2022 Q2
G22894T	S:K444N	mABs	NA	3	2022 Q3
T23042C	S:S494P	mABs	NA	3	2021 Q2, 2022 Q2
A22629C	S:K356T	mABs	NA	2	2022 Q2

Supplementary Table 11 Recurrent mutations previously implicated in antiviral or monoclonal antibody resistance observed across consecutive SARS-CoV-2 sequences. Each infection contributes only once to the counts. Mutation timing is shown at the calendar-quarter level to preserve temporal resolution while avoiding exact dates. Introduction quarters indicate when the relevant treatment was first used in Denmark. RBD = receptor-binding domain; NTD = N-terminal domain. mAB = Monoclonal antibody.

Variable	Nonsynonymous Changes			Synonymous Changes		
	$\hat{\beta}$*	SE	p-value	$\hat{\beta}$*	SE	p-value
Time (Days)						
Per 1 Increase	0.023	0.002	$< 2 \times 10^{-16}$	0.019	0.003	1.27×10^{-13}
Age Group						
0-15	-0.619	0.437	0.157	-0.754	0.546	0.168
15-30	Ref.			Ref.		
30-45	-0.543	0.297	0.068	-0.799	0.380	0.035
45-60	-0.842	0.301	0.005	-1.063	0.388	0.006
60-75	-1.237	0.257	2.15×10^{-5}	-1.273	0.315	5.26×10^{-5}
75+	-1.127	0.273	1.59×10^{-7}	-1.451	0.342	2.18×10^{-5}
Charlson Comorbidity Index†						
Per 1 Increase	0.145	0.046	0.002	-0.029	0.061	0.638
Major Variant						
Alpha	Ref.			Ref.		
Delta	1.751	0.381	4.25×10^{-6}	1.866	0.551	7.03×10^{-4}
Omicron BA.1	1.446	0.437	9.39×10^{-4}	1.948	0.612	0.001
Omicron BA.2	1.620	0.381	2.15×10^{-5}	1.735	0.560	0.002
Omicron BA.5	2.100	0.400	1.59×10^{-7}	1.473	0.621	0.018
*Adjusted for all other variables. SE = Standard Error.						
† Using contemporary diagnoses i.e., those registered up to 2021-12-31, for which diagnostic data is available.						

Supplementary Table 12 Regression results for nonsynonymous and synonymous changes. Estimates ($\hat{\beta}$), standard errors (SE), and p-values are from a zero-inflated negative binomial regression model, adjusted for all other variables. P-values are from two-sided Wald z-tests on the regression coefficients. No adjustments were made for multiple comparisons. Results are shown separately for nonsynonymous and synonymous changes. The Charlson Comorbidity Index was calculated using contemporary diagnoses (up to December 31, 2021). Estimates denote the absolute number of (non)synonymous changes across all coding regions relative to the reference group.

Variable	Nonsynonymous Changes			Synonymous Changes		
	$\hat{\beta}$*	SE	p-value	$\hat{\beta}$*	SE	p-value
**Time (Days)**						
Per 1 Increase	0.023	0.002	$< 2 \times 10^{-16}$	0.019	0.002	3.22×10^{-14}
**Age Group**						
0-15	-0.573	0.442	0.195	-0.699	0.544	0.199
15-30	Ref.			Ref.		
30-45	-0.540	0.301	0.073	-0.725	0.385	0.060
45-60	-0.879	0.318	0.006	-1.014	0.400	0.011
60-75	-0.733	0.289	0.011	-1.215	0.349	0.00049
75+	-1.282	0.305	2.57×10^{-5}	-1.408	0.376	1.79×10^{-4}
**Charlson Comorbidity Index†**						
Per 1 Increase	0.086	0.044	0.053	0.037	0.065	0.567
**Major Variant**						
Alpha	Ref.			Ref.		
Delta	1.696	0.386	1.14×10^{-5}	1.885	0.555	6.74×10^{-4}
Omicron BA.1	1.393	0.454	0.002	1.983	0.631	0.002
Omicron BA.2	1.489	0.408	2.61×10^{-4}	1.753	0.591	0.003
Omicron BA.5	2.038	0.430	2.13×10^{-6}	1.505	0.656	0.022
**Number of vaccinations**						
Per 1 Increase	0.083	0.093	0.370	-0.045	0.121	0.707
*Adjusted for all other variables; † 5-year lookback from 2020-01-01; SE = Standard Error						

Supplementary Table 13 Regression results for nonsynonymous and synonymous changes. Coefficients ($\hat{\beta}$), standard errors (SE), and p-values are from a zero-inflated negative binomial regression model, adjusted for time between samples, major variant, age group, and number of vaccinations. The Charlson Comorbidity Index is based on a 5-year lookback from 2020-01-01. P-values are from two-sided Wald z-tests on the regression coefficients. No adjustments were made for multiple comparisons. SE = Standard Error.

Variable	Rate Ratio (RR)	95% CI	p-value
**Time (Days)**			
Per 1 Increase	1.009	(1.004, 1.013)	1.95×10^{-4}
**Sex**			
Female	0.916	(0.611, 1.373)	0.671
Male	Ref.	–	–
**Number of Vaccinations**			
Per 1 Increase	1.166	(0.955, 1.424)	0.132
**Age Group**			
0-15	0.482	(0.132, 1.756)	0.269
15-30	Ref.	–	–
30-45	0.501	(0.226, 1.109)	0.089
45-60	0.359	(0.164, 0.786)	0.011
60-75	0.451	(0.233, 0.873)	0.019
75+	0.311	(0.151, 0.639)	0.002
All estimates are from a quasi-Poisson regression model.			

Supplementary Table 14 Quasi-Poisson regression results for rate per site, using the first and last sequences for each person to calculate the rate. Among those with at least one consensus-level change, the mean rate was 1.03×10^{-3} substitutions per site per year (95% CI: 2.50×10^{-4}, 2.78×10^{-3}). P-values are from two-sided Wald t-tests on the regression coefficients. No adjustments were made for multiple comparisons.

Variable	Nonsynonymous Changes			Synonymous Changes		
	$\hat{\beta}$*	**SE**	**p-value**	$\hat{\beta}$*	**SE**	**p-value**
**Time (Days)**						
Per 1 Increase	0.023	0.002	$< 2 \times 10^{-16}$	0.019	0.002	3.22×10^{-14}
**Age Group**						
0-15	-0.611	0.442	0.167	-0.682	0.542	0.208
15-30	Ref.			Ref.		
30-45	-0.499	0.297	0.093	-0.746	0.381	0.050
45-60	-0.781	0.298	0.009	-1.052	0.388	0.007
60-75	-0.610	0.254	0.016	-1.270	0.317	6.05×10^{-5}
75+	-1.154	0.268	1.70×10^{-5}	-1.466	0.343	1.96×10^{-5}
**Charlson Comorbidity Index†**						
Per 1 Increase	0.094	0.043	0.029	-0.030	0.062	0.627
**Major Variant**						
Alpha	Ref.			Ref.		
Delta	1.753	0.381	4.08×10^{-6}	1.857	0.550	7.30×10^{-4}
Omicron BA.1	1.507	0.434	5.18×10^{-4}	1.922	0.611	0.002
Omicron BA.2	1.616	0.381	2.28×10^{-5}	1.681	0.560	0.003
Omicron BA.5	2.183	0.398	4.02×10^{-8}	1.421	0.617	0.021
*Adjusted for all other variables; † 5-year lookback from 2020-01-01; SE = Standard Error						

Supplementary Table 15 Regression Results for Nonsynonymous and Synonymous Changes. Coefficients ($\hat{\beta}$), standard errors (SE), and p-values are from a zero-inflated negative binomial regression model, adjusted for time between samples, major variant, and age group. P-values are from two-sided Wald z-tests on the regression coefficients. No adjustments were made for multiple comparisons. The Charlson Comorbidity Index is based on a 5-year lookback from 2020-01-01.

Diagnosis Group†	Nonsynonymous Changes			Synonymous Changes		
	$\hat{\beta}$*	SE	p-value	$\hat{\beta}$*	SE	p-value
**Model 1: Immunosuppression**	0.458	0.105	1.39×10^{-5}	0.082	0.167	0.625
**Model 2: Autoimmune**	0.138	0.216	0.523	-0.291	0.365	0.426
**Model 3: Diabetes**	-0.119	0.182	0.511	-0.365	0.327	0.264
**Model 4: IMID**	-0.370	0.254	0.146	-0.060	0.345	0.861
*Adjusted for time between samples, major variant and age group. IMID = Immune-mediated inflammatory diseases. SE = Standard Error.						
† Using pre-pandemic diagnoses prior to 2020-01-01.						

Supplementary Table 16 Regression Results for Nonsynonymous and Synonymous Changes by Diagnostic Group. Coefficients ($\hat{\beta}$), standard errors (SE), and p-values are from a zero-inflated negative binomial regression model, adjusted for time between samples, major variant, and age group. P-values are from two-sided Wald z-tests on the regression coefficients. No adjustments were made for multiple comparisons. Diagnoses are based on pre-pandemic data (before January 1, 2020).

Amino Acid Substitution	Immunosuppression Diagnosis	Proportion with Immunosuppression
S:E340K	14/15	93.3%
S:R346T	5/7	71.4%
S:K444R	3/5	60.0%
S:R346I	3/3	100%
S:K356T	2/3	66.7%
S:S494P	1/2	50.0%
S:K440R	3/3	100%
S:P9L	3/3	100%
ORF1ab:L3829F	4/7	57.1%

Supplementary Table 17 Amino acid substitutions in the viral genome associated with resistance to monoclonal antibodies, molnupiravir, and nirmatrelvir/ritonavir, and their correlation with immunosuppression diagnosis.

Parameter	Estimate	Est. Error	95% CrI
Intercept	-1.59	0.31	[-2.19, -0.96]
Immunosuppression Diagnosis (β)	-0.28	0.22	[-0.72, 0.15]

Supplementary Table 18 Bayesian logistic regression model results without adjusting for infection duration. The table includes the estimate, standard error, and 95% credible intervals (CrI) for each parameter.

Parameter	Estimate	Est. Error	95% CrI
Intercept	-1.59	0.31	[-2.19, -0.96]
Immunosuppression Diagnosis (β)	-0.29	0.23	[-0.75, 0.15]
Infection Duration	0.00	0.01	[-0.01, 0.01]

Supplementary Table 19 Bayesian logistic regression model results adjusting for infection duration. The table includes the estimate, standard error, and 95% credible intervals (CrI) for each parameter.

Variable	Rate Ratio (RR)	95% CI	p-value
**Total Days Between Sequences (Per Day Increase)**	1.009	(1.004, 1.013)	2.33×10^{-4}
**Age Group**			
0-15	0.441	(0.123, 1.570)	0.207
30-45	0.572	(0.261, 1.253)	0.164
45-60	0.410	(0.194, 0.862)	0.020
60-75	0.569	(0.320, 1.013)	0.056
75+	0.401	(0.213, 0.753)	0.005
**Rebound During Infection**	1.279	(0.800, 2.046)	0.305
**Charlson Index (5 years)**	1.083	(0.970, 1.208)	0.158
All estimates are from a quasi-Poisson regression model.			

Supplementary Table 20 Quasi-Poisson regression results for rate per site, adjusted for total days between sequences, age group, rebound status, and Charlson index (5 year lookback). P-values are from two-sided Wald t-tests on the regression coefficients. No adjustments were made for multiple comparisons.

ICD Code	Description	Category
B20	Human immunodeficiency virus disease resulting in infectious and parasitic diseases	HIV
B20.0	HIV disease resulting in mycobacterial infection	HIV
B20.1	HIV disease resulting in other bacterial infections	HIV
B20.2	HIV disease resulting in cytomegaloviral disease	HIV
B20.3	HIV disease resulting in other viral infections	HIV
B20.4	HIV disease resulting in candidiasis	HIV
B20.5	HIV disease resulting in other mycoses	HIV
B20.6	HIV disease resulting in Pneumocystis carinii pneumonia	HIV
B20.7	HIV disease resulting in multiple infections	HIV
B20.8	HIV disease resulting in other infectious and parasitic diseases	HIV
B20.9	HIV disease resulting in unspecified infectious or parasitic diseases	HIV
B21	Human immunodeficiency virus disease resulting in malignant neoplasms	HIV
B21.0	HIV disease resulting in Kaposi's sarcoma	HIV
B21.1	HIV disease resulting in Burkitt's lymphoma	HIV
B21.2	HIV disease resulting in other types of non-Hodgkin's lymphoma	HIV
B21.3	HIV disease resulting in other malignant neoplasms of lymphoid, haematopoietic and related tissue	HIV
B21.7	HIV disease resulting in multiple malignant neoplasms	HIV
B21.8	HIV disease resulting in other malignant neoplasms	HIV
B21.9	HIV disease resulting in unspecified malignant neoplasm	HIV
B22	Human immunodeficiency virus disease resulting in other specified diseases	HIV
B22.0	HIV disease resulting in encephalopathy	HIV
B22.1	HIV disease resulting in lymphoid interstitial pneumonitis	HIV

ICD Code	Description	Category
B22.2	HIV disease resulting in wasting syndrome	HIV
B22.7	HIV disease resulting in multiple diseases classified elsewhere	HIV
B23	Human immunodeficiency virus disease resulting in other conditions	HIV
B23.0	Acute HIV infection syndrome	HIV
B23.1	HIV disease resulting in (persistent) generalized lymphadenopathy	HIV
B23.2	HIV disease resulting in haematologic and immunologic abnormalities NEC	HIV
B23.8	HIV disease resulting in other specified conditions	HIV
B24	Unspecified human immunodeficiency virus [HIV] disease	HIV
C81	Hodgkin's disease	Lymph
C81.0	Hodgkin's disease, lymphocytic predominance	Lymph
C81.1	Hodgkin's disease, nodular sclerosis	Lymph
C81.2	Hodgkin's disease, mixed cellularity	Lymph
C81.3	Hodgkin's disease, lymphocytic depletion	Lymph
C81.7	Other Hodgkin's disease	Lymph
C81.9	Hodgkin's disease, unspecified	Lymph
C82	Follicular [nodular] non-Hodgkin's lymphoma	Lymph
C82.0	Follicular non-Hodgkin's small cleaved cell lymphoma	Lymph
C82.1	Follicular non-Hodgkin's mixed small cleaved and large cell lymphoma	Lymph
C82.2	Follicular non-Hodgkin's large cell lymphoma	Lymph
C82.7	Follicular non-Hodgkin's other types of lymphoma	Lymph
C82.9	Follicular non-Hodgkin's unspecified lymphoma	Lymph
C83	Diffuse non-Hodgkin's lymphoma	Lymph

ICD Code	Description	Category
C83.0	Diffuse non-Hodgkin's small cell (diffuse) lymphoma	Lymph
C83.1	Diffuse non-Hodgkin's small cleaved cell (diffuse) lymphoma	Lymph
C83.2	Diffuse non-Hodgkin's mixed small and large cell (diffuse) lymphoma	Lymph
C83.3	Diffuse non-Hodgkin's large cell (diffuse) lymphoma	Lymph
C83.4	Diffuse non-Hodgkin's immunoblastic (diffuse) lymphoma	Lymph
C83.5	Diffuse non-Hodgkin's lymphoblastic (diffuse) lymphoma	Lymph
C83.6	Diffuse non-Hodgkin's undifferentiated (diffuse) lymphoma	Lymph
C83.7	Diffuse non-Hodgkin's Burkitt's tumour	Lymph
C83.8	Other types of diffuse non-Hodgkin's lymphoma	Lymph
C83.9	Diffuse non-Hodgkin's lymphoma, unspecified	Lymph
C84	Peripheral and cutaneous T-cell lymphomas	Lymph
C84.0	Peripheral and cutaneous T-cell lymphomas, mycosis fungoides	Lymph
C84.1	Peripheral and cutaneous T-cell lymphomas, Sezary's disease	Lymph
C84.2	Peripheral and cutaneous T-cell lymphomas, T-zone lymphoma	Lymph
C84.3	Peripheral and cutaneous T-cell lymphomas, lymphoepithelioid lymphoma	Lymph
C84.4	Peripheral and cutaneous T-cell lymphomas, peripheral T-cell lymphoma	Lymph
C84.5	Peripheral and cutaneous T-cell lymphomas, other and unspecified T-cell lymphomas	Lymph
C85	Other and unspecified types of non-Hodgkin's lymphoma	Lymph
C85.0	Other and unspecified types of non-Hodgkin's lymphoma, lymphosarcoma	Lymph
C85.1	Other and unspecified types of non-Hodgkin's B-cell lymphoma, unspecified	Lymph

ICD Code	Description	Category
C85.7	Other specified types of non-Hodgkin's lymphoma	Lymph
C85.9	Non-Hodgkin's lymphoma, unspecified type	Lymph
C88	Malignant immunoproliferative diseases	Lymph
C88.0	Waldenstrom's macroglobulinaemia	Lymph
C88.1	Alpha heavy chain disease	Lymph
C88.2	Gamma heavy chain disease	Lymph
C88.3	Malignant immunoproliferative small intestinal disease	Lymph
C88.7	Other malignant immunoproliferative diseases	Lymph
C88.9	Malignant immunoproliferative disease, unspecified	Lymph
C90	Multiple myeloma and malignant plasma cell neoplasms	Myeloma
C90.0	Multiple myeloma	Myeloma
C90.1	Plasma cell leukaemia	Myeloma
C90.2	Malignant plasma cell neoplasm, extramedullary plasmacytoma	Myeloma
C91	Lymphoid leukaemia	Leukemia
C91.0	Acute lymphoblastic leukaemia	Leukemia
C91.1	Chronic lymphocytic leukaemia	Leukemia
C91.2	Subacute lymphocytic leukaemia	Leukemia
C91.3	Prolymphocytic leukaemia	Leukemia
C91.4	Hairy-cell leukaemia	Leukemia
C91.5	Adult T-cell leukaemia	Leukemia
C91.7	Other lymphoid leukaemia	Leukemia
C91.9	Lymphoid leukaemia, unspecified	Leukemia
C92	Myeloid leukaemia	Leukemia
C92.0	Acute myeloid leukaemia	Leukemia
C92.1	Chronic myeloid leukaemia	Leukemia
C92.2	Subacute myeloid leukaemia	Leukemia

ICD Code	Description	Category
C92.3	Myeloid sarcoma	Leukemia
C92.4	Acute promyelocytic leukaemia	Leukemia
C92.5	Acute myelomonocytic leukaemia	Leukemia
C92.7	Other myeloid leukaemia	Leukemia
C92.9	Myeloid leukaemia, unspecified	Leukemia
C93	Monocytic leukaemia	Leukemia
C93.0	Acute monocytic leukaemia	Leukemia
C93.1	Chronic monocytic leukaemia	Leukemia
C93.2	Subacute monocytic leukaemia	Leukemia
C93.7	Other monocytic leukaemia	Leukemia
C93.9	Monocytic leukaemia, unspecified	Leukemia
C94	Other leukaemias of specified cell type	Leukemia
C94.0	Acute erythroid leukaemia	Leukemia
C94.1	Chronic erythroid leukaemia	Leukemia
C94.2	Acute megakaryoblastic leukaemia	Leukemia
C94.3	Acute panmyelosis with myelofibrosis	Leukemia
C94.4	Acute myelofibrosis	Leukemia
C94.5	Acute leukaemia of ambiguous lineage	Leukemia
C94.7	Other specified leukaemias of specified cell type	Leukemia
C94.9	Unspecified leukaemia	Leukemia

Supplementary Table 21: Immunosuppression Diagnosis Codes.

From Mansfield et al. [1].

ICD Code	Description	Category
E10	Insulin-dependent diabetes mellitus	T1DM
E10.0	Insulin-dependent diabetes mellitus with coma	T1DM
E10.1	Insulin-dependent diabetes mellitus with ketoacidosis	T1DM
E10.2	Insulin-dependent diabetes mellitus with renal complications	T1DM
E10.3	Insulin-dependent diabetes mellitus with ophthalmic comps	T1DM
E10.4	Insulin-dependent diabetes mellitus with neurological comps	T1DM
E10.5	Insulin-dependent diabetes mellitus with periph circ comps	T1DM
E10.6	Insulin-dependent diabetes mellitus with other spec comps	T1DM
E10.7	Insulin-dependent diabetes mellitus with multiple comps	T1DM
E10.8	Insulin-dependent diabetes mellitus with unspec comps	T1DM
E10.9	Insulin-dependent diabetes mellitus without complications	T1DM
E11	Non-insulin-dependent diabetes mellitus	T2DM
E11.0	Non-insulin-dependent diabetes mellitus with coma	T2DM
E11.1	Non-insulin-dependent diabetes mellitus with ketoacidosis	T2DM
E11.2	Non-insulin-dependent diabetes mellitus with renal comps	T2DM
E11.3	Non-insulin-dependent diabetes mellitus with ophthalm comps	T2DM
E11.4	Non-insulin-dependent diabetes mellitus with neuro comps	T2DM
E11.5	Non-insulin-depend diabetes mellitus with periph circ comp	T2DM
E11.6	Non-insulin-depend diabetes mellitus with other spec comp	T2DM
E11.7	Non-insulin-dependent diabetes mellitus with multiple comps	T2DM
E11.8	Non-insulin-dependent diabetes mellitus with unspec comps	T2DM
E11.9	Non-insulin-depend diabetes mellitus without complication	T2DM
E13	Other specified diabetes mellitus	DM NOS
E13.0	Other specified diabetes mellitus with coma	DM NOS
E13.1	Other specified diabetes mellitus with ketoacidosis	DM NOS
E13.2	Other specified diabetes mellitus with renal complications	DM NOS

ICD Code	Description	Category
E13.3	Other specified diabetes mellitus with ophthalmic complications	DM NOS
E13.4	Other specified diabetes mellitus with neurological comps	DM NOS
E13.5	Other specified diabetes mellitus with periph circulatory comps	DM NOS
E13.6	Other specified diabetes mellitus with other specified comps	DM NOS
E13.7	Other specified diabetes mellitus with multiple complications	DM NOS
E13.8	Other specified diabetes mellitus with unspecified comps	DM NOS
E13.9	Other specified diabetes mellitus without complications	DM NOS
E14	Unspecified diabetes mellitus	DM NOS
E14.0	Unspecified diabetes mellitus with coma	DM NOS
E14.1	Unspecified diabetes mellitus with ketoacidosis	DM NOS
E14.2	Unspecified diabetes mellitus with renal complications	DM NOS
E14.3	Unspecified diabetes mellitus with ophthalmic complications	DM NOS
E14.4	Unspecified diabetes mellitus with neurological comps	DM NOS
E14.5	Unspecified diabetes mellitus with periph circulatory comps	DM NOS
E14.6	Unspecified diabetes mellitus with other specified comps	DM NOS
E14.7	Unspecified diabetes mellitus with multiple complications	DM NOS
E14.8	Unspecified diabetes mellitus with unspecified complications	DM NOS
E14.9	Unspecified diabetes mellitus without complications	DM NOS
G59.0	Diabetic mononeuropathy	DM NOS
G63.2	Diabetic polyneuropathy	DM NOS
H28.0	Diabetic cataract	DM NOS
H36.0	Diabetic retinopathy	DM NOS
M14.2	Diabetic arthropathy	DM NOS
N08.3	Glomerular disorders in diabetes mellitus	DM NOS
O24.0	Pre-existing diabetes mellitus, insulin-dependent	T1DM
O24.1	Pre-existing diabetes mellitus, non-insulin-dependent	T2DM

ICD Code	Description	Category
O24.3	Pre-existing diabetes mellitus, unspecified	DM NOS
Y42.3	Insulin and oral hypoglycaemic [antidiabetic] drugs	Possible

Supplementary Table 22: Diabetes ICD-10 Codes. From Mansfield et al. [2].

ICD Code	Description
D51.0	Pernicious anemia
D59.1	Autoimmune hemolytic anemia
D69.3	Idiopathic thrombocytopenic purpura
E05.00	Thyrotoxicosis with diffuse goiter without thyrotoxic crisis or storm
E05.01	Thyrotoxicosis with diffuse goiter with thyrotoxic crisis or storm
E05.10	Thyrotoxicosis with toxic single thyroid nodule without thyrotoxic crisis or storm
E05.11	Thyrotoxicosis with toxic single thyroid nodule with thyrotoxic crisis or storm
E05.20	Thyrotoxicosis with toxic multinodular goiter without thyrotoxic crisis or storm
E05.21	Thyrotoxicosis with toxic multinodular goiter with thyrotoxic crisis or storm
E05.30	Thyrotoxicosis from ectopic thyroid tissue without thyrotoxic crisis or storm
E05.31	Thyrotoxicosis from ectopic thyroid tissue with thyrotoxic crisis or storm
E05.80	Other thyrotoxicosis without thyrotoxic crisis or storm
E05.81	Other thyrotoxicosis with thyrotoxic crisis or storm
E05.90	Thyrotoxicosis, unspecified without thyrotoxic crisis or storm
E05.91	Thyrotoxicosis, unspecified with thyrotoxic crisis or storm
E06.3	Autoimmune thyroiditis
E27.1	Primary adrenocortical insufficiency
G35	Multiple sclerosis
G61.0	Guillain–Barre syndrome
H20.00	Unspecified acute and subacute iridocyclitis
H20.011	Primary iridocyclitis, right eye
H20.012	Primary iridocyclitis, left eye
H20.013	Primary iridocyclitis, bilateral
H20.019	Primary iridocyclitis, unspecified eye
H20.021	Recurrent acute iridocyclitis, right eye

ICD Code	Description
H20.022	Recurrent acute iridocyclitis, left eye
H20.023	Recurrent acute iridocyclitis, bilateral
H20.029	Recurrent acute iridocyclitis, unspecified eye
H20.031	Secondary infectious iridocyclitis, right eye
H20.032	Secondary infectious iridocyclitis, left eye
H20.033	Secondary infectious iridocyclitis, bilateral
H20.039	Secondary infectious iridocyclitis, unspecified eye
H20.041	Secondary noninfectious iridocyclitis, right eye
H20.042	Secondary noninfectious iridocyclitis, left eye
H20.043	Secondary noninfectious iridocyclitis, bilateral
H20.049	Secondary noninfectious iridocyclitis, unspecified eye
H20.051	Hypopyon, right eye
H20.052	Hypopyon, left eye
H20.053	Hypopyon, bilateral
H20.059	Hypopyon, unspecified eye
H20.10	Chronic iridocyclitis, unspecified eye
H20.11	Chronic iridocyclitis, right eye
H20.12	Chronic iridocyclitis, left eye
H20.13	Chronic iridocyclitis, bilateral
H20.811	Fuchs' heterochromic cyclitis, right eye
H20.812	Fuchs' heterochromic cyclitis, left eye
H20.813	Fuchs' heterochromic cyclitis, bilateral
H20.819	Fuchs' heterochromic cyclitis, unspecified eye
H20.821	Vogt-Koyanagi syndrome, right eyeVogt-Koyanagi syndrome, right eye
H20.822	Vogt-Koyanagi syndrome, left eye
H20.823	Vogt-Koyanagi syndrome, bilateral

ICD Code	Description
H20.824	Vogt-Koyanagi syndrome, unspecified eye
H20.9	Iridocyclitis unspecified
K50.00	Crohn’s disease of small intestine without complications
K50.011	Crohn’s disease of small intestine with rectal bleeding
K50.012	Crohn’s disease of small intestine with intestinal obstruction
K50.013	Crohn’s disease of small intestine with fistula
K50.014	Crohn’s disease of small intestine with abscess
K50.018	Crohn’s disease of small intestine with other complication
K50.019	Crohn’s disease of small intestine with unspecified complications
K50.10	Crohn’s disease of large intestine without complications
K50.111	Crohn’s disease of large intestine with rectal bleeding
K50.112	Crohn’s disease of large intestine with intestinal obstruction
K50.113	Crohn’s disease of large intestine with fistula
K50.114	Crohn’s disease of large intestine with abscess
K50.118	Crohn’s disease of large intestine with other complication
K50.119	Crohn’s disease of large intestine with unspecified complications
K50.80	Crohn’s disease of both small and large intestine without complications
K50.811	Crohn’s disease of both small and large intestine with rectal bleeding
K50.812	Crohn’s disease of both small and large intestine with intestinal obstruction
K50.813	Crohn’s disease of both small and large intestine with fistula
K50.814	Crohn’s disease of both small and large intestine with abscess
K50.818	Crohn’s disease of both small and large intestine with other complication
K50.819	Crohn’s disease of both small and large intestine with unspecified complications
K50.90	Crohn’s disease, unspecified, without complications
K50.911	Crohn’s disease, unspecified, with rectal bleeding
K50.912	Crohn’s disease, unspecified, with intestinal obstruction

ICD Code	Description
K50.913	Crohn's disease, unspecified, with fistula
K50.914	Crohn's disease, unspecified, with abscess
K50.918	Crohn's disease, unspecified, with other complication
K50.919	Crohn's disease, unspecified, with unspecified complications
K50.40	Arthropathy in Crohn's disease [Regional enteritis] (K50.-+) multiple sites
K50.41	Arthropathy in Crohn's disease [Regional enteritis] (K50.-+) shoulder region
K50.42	Arthropathy in Crohn's disease [Regional enteritis] (K50.-+) upper arm
K50.43	Arthropathy in Crohn's disease [Regional enteritis] (K50.-+) forearm
K50.44	Arthropathy in Crohn's disease [Regional enteritis] (K50.-+) hand
K50.45	Arthropathy in Crohn's disease [Regional enteritis] (K50.-+) pelvic region and thigh
K50.46	Arthropathy in Crohn's disease [Regional enteritis] (K50.-+) lower leg
K50.47	Arthropathy in Crohn's disease [Regional enteritis] (K50.-+) ankle and foot
K50.48	Arthropathy in Crohn's disease [Regional enteritis] (K50.-+) other site
K50.49	Arthropathy in Crohn's disease [Regional enteritis] (K50.-+) site unspecified
M09.10	Juvenile arthritis in Crohn's disease [Regional enteritis] (K50.-+) multiple sites
M09.11	Juvenile arthritis in Crohn's disease [Regional enteritis] (K50.-+) shoulder region
M09.12	Juvenile arthritis in Crohn's disease [Regional enteritis] (K50.-+) upper arm
M09.13	Juvenile arthritis in Crohn's disease [Regional enteritis] (K50.-+) forearm
M09.14	Juvenile arthritis in Crohn's disease [Regional enteritis] (K50.-+) hand
M09.15	Juvenile arthritis in Crohn's disease [Regional enteritis] (K50.-+) pelvic region and thigh
M09.16	Juvenile arthritis in Crohn's disease [Regional enteritis] (K50.-+) lower leg
M09.17	Juvenile arthritis in Crohn's disease [Regional enteritis] (K50.-+) ankle and foot

ICD Code	Description
M09.18	Juvenile arthritis in Crohn's disease [Regional enteritis] (K50.-+) other site
M09.19	Juvenile arthritis in Crohn's disease [Regional enteritis] (K50.-+) site unspecified
K51.00	Ulcerative (chronic) pancolitis without complications
K51.011	Ulcerative (chronic) pancolitis with rectal bleeding
K51.012	Ulcerative (chronic) pancolitis with intestinal obstruction
K51.013	Ulcerative (chronic) pancolitis with fistula
K51.014	Ulcerative (chronic) pancolitis with abscess
K51.018	Ulcerative (chronic) pancolitis with other complication
K51.019	Ulcerative (chronic) pancolitis with unspecifed complications
K51.20	Ulcerative (chronic) proctitis without complications
K51.211	Ulcerative (chronic) proctitis with rectal bleeding
K51.212	Ulcerative (chronic) proctitis with intestinal obstruction
K51.213	Ulcerative (chronic) proctitis with fistula
K51.214	Ulcerative (chronic) proctitis with abscess
K51.218	Ulcerative (chronic) proctitis with other complication
K51.219	Ulcerative (chronic) proctitis with unspecified complications
K51.30	Ulcerative (chronic) rectosigmoiditis without complications
K51.311	Ulcerative (chronic) rectosigmoiditis with rectal bleeding
K51.312	Ulcerative (chronic) rectosigmoiditis with intestinal obstruction
K51.313	Ulcerative (chronic) rectosigmoiditis with fistula
K51.314	Ulcerative (chronic) rectosigmoiditis with abscess
K51.318	Ulcerative (chronic) rectosigmoiditis with other complication
K51.319	Ulcerative (chronic) rectosigmoiditis with unspecified complications
K51.40	Inflammatory polyps of colon without complications
K51.411	Inflammatory polyps of colon with rectal bleeding

ICD Code	Description
K51.412	Inflammatory polyps of colon with intestinal obstruction
K51.413	Inflammatory polyps of colon with fistula
K51.414	Inflammatory polyps of colon with abscess
K51.418	Inflammatory polyps of colon with other complication
K51.419	Inflammatory polyps of colon with unspecified complications
K51.50	Left sided colitis without complications
K51.511	Left sided colitis with rectal bleeding
K51.512	Left sided colitis with intestinal obstruction
K51.513	Left sided colitis with fistula
K51.514	Left sided colitis with abscess
K51.518	Left sided colitis with other complication
K51.519	Left sided colitis with unspecified complications
K51.80	Other ulcerative colitis without complications
K51.811	Other ulcerative colitis with rectal bleeding
K51.812	Other ulcerative colitis with intestinal obstruction
K51.813	Other ulcerative colitis with fistula
K51.814	Other ulcerative colitis with abscess
K51.818	Other ulcerative colitis with other complication
K51.819	Other ulcerative colitis with unspecified complications
K51.90	Ulcerative colitis, unspecified without complications
K51.911	Ulcerative colitis, unspecified with rectal bleeding
K51.912	Ulcerative colitis, unspecified with intestinal obstruction
K51.913	Ulcerative colitis, unspecified with fistula
K51.914	Ulcerative colitis, unspecified with abscess
K51.918	Ulcerative colitis, unspecified with other complication
K51.919	Ulcerative colitis, unspecified with unspecified complications

ICD Code	Description
M07.50	Arthropathy in ulcerative colitis (K51.-+) multiple sites
M07.51	Arthropathy in ulcerative colitis (K51.-+) shoulder region
M07.52	Arthropathy in ulcerative colitis (K51.-+) upper arm
M07.53	Arthropathy in ulcerative colitis (K51.-+) forearm
M07.54	Arthropathy in ulcerative colitis (K51.-+) hand
M07.55	Arthropathy in ulcerative colitis (K51.-+) pelvic region and thigh
M07.56	Arthropathy in ulcerative colitis (K51.-+) lower leg
M07.57	Arthropathy in ulcerative colitis (K51.-+) ankle and foot
M07.58	Arthropathy in ulcerative colitis (K51.-+) other site
M07.59	Arthropathy in ulcerative colitis (K51.-+) site unspecified
M09.20	Juvenile arthritis in ulcerative colitis (K51.-+) multiple sites
M09.21	Juvenile arthritis in ulcerative colitis (K51.-+) shoulder region
M09.22	Juvenile arthritis in ulcerative colitis (K51.-+) upper arm
M09.23	Juvenile arthritis in ulcerative colitis (K51.-+) forearm
M09.24	Juvenile arthritis in ulcerative colitis (K51.-+) hand
M09.25	Juvenile arthritis in ulcerative colitis (K51.-+) pelvic region and thigh
M09.26	Juvenile arthritis in ulcerative colitis (K51.-+) lower leg
M09.27	Juvenile arthritis in ulcerative colitis (K51.-+) ankle and foot
M09.28	Juvenile arthritis in ulcerative colitis (K51.-+) other site
M09.29	Juvenile arthritis in ulcerative colitis (K51.-+) site unspecified
K74.3	Primary biliary cirrhosis
K75.4	Autoimmune hepatitis
K90.0	Celiac disease
L10.0	Pemphigus vulgaris
L10.1	Pemphigus vegetans
L10.2	Pemphigus foliaceus

ICD Code	Description
L10.3	Brazilian pemphigus
L10.4	Pemphigus erythematosus
L10.81	Paraneoplastic pemphigus
L10.89	Other pemphigus
L10.9	Pemphigus, unspecified
L12.0	Bullous pemphigoid
L12.1	Cicatricial pemphigoid
L12.2	Chronic bullous disease of childhood
L12.8	Other pemphigoid
L12.9	Pemphigoid, unspecified
H13.3	Ocular pemphigoid
L40.0	Psoriasis vulgaris
L40.1	Generalised pustular psoriasis
L40.2	Acrodermatitis continua
L40.3	Pustulosis palmaris et plantaris
L40.4	Guttate psoriasis
L40.50	Arthropathic psoriasis, unspecified
L40.51	Distal interphalangeal psoriatic arthropathy
L40.52	Psoriatic arthritis mutilans
L40.53	Psoriatic spondylitis
L40.54	Psoriatic juvenile arthropathy
L40.59	Other psoriatic arthropathy
L40.8	Other psoriasis
L40.9	Psoriasis, unspecified
M07.00	Distal interphalangeal psoriatic arthropathy (L40.5+) multiple sites
M07.04	Distal interphalangeal psoriatic arthropathy (L40.5+) hand

ICD Code	Description
M07.07	Distal interphalangeal psoriatic arthropathy (L40.5+) ankle and foot
M07.09	Distal interphalangeal psoriatic arthropathy (L40.5+) site unspecified
M07.10	Arthritis mutilans (L40.5+) multiple sites
M07.11	Arthritis mutilans (L40.5+) shoulder region
M07.12	Arthritis mutilans (L40.5+) upper arm
M07.13	Arthritis mutilans (L40.5+) forearm
M07.14	Arthritis mutilans (L40.5+) hand
M07.15	Arthritis mutilans (L40.5+) pelvic region and thigh
M07.16	Arthritis mutilans (L40.5+) lower leg
M07.17	Arthritis mutilans (L40.5+) ankle and foot
M07.18	Arthritis mutilans (L40.5+) other site
M07.19	Arthritis mutilans (L40.5+) site unspecified
M07.20	Psoriatic spondylitis (L40.5+) multiple sites
M07.21	Psoriatic spondylitis (L40.5+) shoulder region
M07.22	Psoriatic spondylitis (L40.5+) upper arm
M07.23	Psoriatic spondylitis (L40.5+) forearm
M07.24	Psoriatic spondylitis (L40.5+) hand
M07.25	Psoriatic spondylitis (L40.5+) pelvic region and thigh
M07.26	Psoriatic spondylitis (L40.5+) lower leg
M07.27	Psoriatic spondylitis (L40.5+) ankle and foot
M07.28	Psoriatic spondylitis (L40.5+) other site
M07.29	Psoriatic spondylitis (L40.5+) site unspecified
M07.30	Other psoriatic arthropathies (L40.5+) multiple sites
M07.31	Other psoriatic arthropathies (L40.5+) shoulder region
M07.32	Other psoriatic arthropathies (L40.5+) upper arm
M07.33	Other psoriatic arthropathies (L40.5+) forearm

ICD Code	Description
M07.34	Other psoriatic arthropathies (L40.5+) hand
M07.35	Other psoriatic arthropathies (L40.5+) pelvic region and thigh
M07.36	Other psoriatic arthropathies (L40.5+) lower leg
M07.37	Other psoriatic arthropathies (L40.5+) ankle and foot
M07.38	Other psoriatic arthropathies (L40.5+) other site
M07.39	Other psoriatic arthropathies (L40.5+) site unspecified
M09.00	Juvenile arthritis in psoriasis (L40.5+) multiple sites
M09.01	Juvenile arthritis in psoriasis (L40.5+) shoulder region
M09.02	Juvenile arthritis in psoriasis (L40.5+) upper arm
M09.03	Juvenile arthritis in psoriasis (L40.5+) forearm
M09.04	Juvenile arthritis in psoriasis (L40.5+) hand
M09.05	Juvenile arthritis in psoriasis (L40.5+) pelvic region and thigh
M09.06	Juvenile arthritis in psoriasis (L40.5+) lower leg
M09.07	Juvenile arthritis in psoriasis (L40.5+) ankle and foot
M09.08	Juvenile arthritis in psoriasis (L40.5+) other site
M09.09	Juvenile arthritis in psoriasis (L40.5+) site unspecified
L63.0	Alopecia (capitis) totalis
L63.1	Alopecia universalis
L63.2	Ophiasis
L63.8	Other alopecia areata
L63.9	Alopecia areata, unspecified
L80	Vitiligo
M05.00	Felty's syndrome, unspecified site
M05.011	Felty's syndrome, right shoulder
M05.012	Felty's syndrome, left shoulder
M05.019	Felty's syndrome, unspecified shoulder

ICD Code	Description
M05.021	Felty’s syndrome, right elbow
M05.022	Felty’s syndrome, left elbow
M05.029	Felty’s syndrome, unspecified elbow
M05.031	Felty’s syndrome, right wrist
M05.032	Felty’s syndrome, left wrist
M05.039	Felty’s syndrome, unspecified wrist
M05.041	Felty’s syndrome, right hand
M05.042	Felty’s syndrome, left hand
M05.049	Felty’s syndrome, unspecified hand
M05.051	Felty’s syndrome, right hip
M05.052	Felty’s syndrome, left hip
M05.059	Felty’s syndrome, unspecified hip
M05.061	Felty’s syndrome, right knee
M05.062	Felty’s syndrome, left knee
M05.069	Felty’s syndrome, unspecified knee
M05.071	Felty’s syndrome, right ankle and foot
M05.072	Felty’s syndrome, left ankle and foot
M05.079	Felty’s syndrome, unspecified ankle and foot
M05.09	Felty’s syndrome, multiple sites
M05.10	Rheumatoid lung disease with rheumatoid arthritis of unspecified site
M05.111	Rheumatoid lung disease with rheumatoid arthritis of right shoulder
M05.112	Rheumatoid lung disease with rheumatoid arthritis of left shoulder
M05.119	Rheu lung disease with rheumatoid arthritis of unspecified shoulder
M05.121	Rheumatoid lung disease with rheumatoid arthritis of right elbow
M05.122	Rheumatoid lung disease with rheumatoid arthritis of left elbow
M05.129	Rheumatoid lung disease with rheumatoid arthritis of unspecified elbow

ICD Code	Description
M05.131	Rheumatoid lung disease with rheumatoid arthritis of right wrist
M05.132	Rheumatoid lung disease with rheumatoid arthritis of left wrist
M05.139	Rheumatoid lung disease with rheumatoid arthritis of unspecified wrist
M05.141	Rheumatoid lung disease with rheumatoid arthritis of right hand
M05.142	Rheumatoid lung disease with rheumatoid arthritis of left hand
M05.149	Rheumatoid lung disease with rheumatoid arthritis of unspecified hand
M05.151	Rheumatoid lung disease with rheumatoid arthritis of right hip
M05.152	Rheumatoid lung disease with rheumatoid arthritis of left hip
M05.159	Rheumatoid lung disease with rheumatoid arthritis of unspecified hip
M05.161	Rheumatoid lung disease with rheumatoid arthritis of right knee
M05.162	Rheumatoid lung disease with rheumatoid arthritis of left knee
M05.169	Rheumatoid lung disease with rheumatoid arthritis of unspecified knee
M05.171	Rheumatoid lung disease with rheumatoid arthritis of right ankle/foot
M05.172	Rheumatoid lung disease with rheumatoid arthritis of left ankle/foot
M05.179	Rheumatoid lung disease with rheumatoid arthritis of unspecified ankle/foot
M05.19	Rheumatoid lung disease with rheumatoid arthritis multiple sites
M05.20	Rheumatoid vasculitis with rheumatoid arthritis of unspecified site
M05.211	Rheumatoid vasculitis with rheumatoid arthritis of right shoulder
M05.212	Rheumatoid vasculitis with rheumatoid arthritis of left shoulder
M05.219	Rheumatoid vasculitis with rheumatoid arthritis of unspecified shoulder
M05.221	Rheumatoid vasculitis with rheumatoid arthritis of right elbow
M05.222	Rheumatoid vasculitis with rheumatoid arthritis of left elbow
M05.229	Rheumatoid vasculitis with rheumatoid arthritis of unspecified elbow
M05.231	Rheumatoid vasculitis with rheumatoid arthritis of right wrist
M05.232	Rheumatoid vasculitis with rheumatoid arthritis of left wrist
M05.239	Rheumatoid vasculitis with rheumatoid arthritis of unspecified wrist

ICD Code	Description
M05.241	Rheumatoid vasculitis with rheumatoid arthritis of right hand
M05.242	Rheumatoid vasculitis with rheumatoid arthritis of left hand
M05.249	Rheumatoid vasculitis with rheumatoid arthritis of unspecified hand
M05.251	Rheumatoid vasculitis with rheumatoid arthritis of right hip
M05.252	Rheumatoid vasculitis with rheumatoid arthritis of left hip
M05.259	Rheumatoid vasculitis with rheumatoid arthritis of unspecified hip
M05.261	Rheumatoid vasculitis with rheumatoid arthritis of right knee
M05.262	Rheumatoid vasculitis with rheumatoid arthritis of left knee
M05.269	Rheumatoid vasculitis with rheumatoid arthritis of unspecified knee
M05.271	Rheumatoid vasculitis with rheumatoid arthritis of right ankle/foot
M05.272	Rheumatoid vasculitis with rheumatoid arthritis of left ankle/foot
M05.279	Rheumatoid vasculitis with rheumatoid arthritis of unspecified ankle/foot
M05.29	Rheumatoid vasculitis with rheumatoid arthritis multiple sites
M05.30	Rheumatoid heart disease with rheumatoid arthritis of unspecified site
M05.311	Rheumatoid heart disease with rheumatoid arthritis of right shoulder
M05.312	Rheumatoid heart disease with rheumatoid arthritis of left shoulder
M05.319	Rheumatoid heart disease with rheumatoid arthritis of unspecified shoulder
M05.321	Rheumatoid heart disease with rheumatoid arthritis of right elbow
M05.322	Rheumatoid heart disease with rheumatoid arthritis of left elbow
M05.329	Rheumatoid heart disease with rheumatoid arthritis of unspecified elbow
M05.331	Rheumatoid heart disease with rheumatoid arthritis of right wrist
M05.332	Rheumatoid heart disease with rheumatoid arthritis of left wrist
M05.339	Rheumatoid heart disease with rheumatoid arthritis of unspecified wrist
M05.341	Rheumatoid heart disease with rheumatoid arthritis of right hand
M05.342	Rheumatoid heart disease with rheumatoid arthritis of left hand
M05.349	Rheumatoid heart disease with rheumatoid arthritis of unspecified hand

ICD Code	Description
M05.351	Rheumatoid heart disease with rheumatoid arthritis of right hip
M05.352	Rheumatoid heart disease with rheumatoid arthritis of left hip
M05.359	Rheumatoid heart disease with rheumatoid arthritis of unspecified hip
M05.361	Rheumatoid heart disease with rheumatoid arthritis of right knee
M05.362	Rheumatoid heart disease with rheumatoid arthritis of left knee
M05.369	Rheumatoid heart disease with rheumatoid arthritis of unspecified knee
M05.371	Rheumatoid heart disease with rheumatoid arthritis of right ankle/foot
M05.372	Rheumatoid heart disease with rheumatoid arthritis of left ankle/foot
M05.379	Rheumatoid heart disease with rheumatoid arthritis of unspecified ankle/foot
M05.39	Rheumatoid heart disease with rheumatoid arthritis multiple sites
M05.80	Other rheumatoid arthritis with rheumatoid factor of unspecified site
M05.811	Other rheumatoid arthritis with rheumatoid factor of right shoulder
M05.812	Other rheumatoid arthritis with rheumatoid factor of left shoulder
M05.819	Other rheumatoid arthritis with rheumatoid factor of unspecified shoulder
M05.821	Other rheumatoid arthritis with rheumatoid factor of right elbow
M05.822	Other rheumatoid arthritis with rheumatoid factor of left elbow
M05.829	Other rheumatoid arthritis with rheumatoid factor of unspecified elbow
M05.831	Other rheumatoid arthritis with rheumatoid factor of right wrist
M05.832	Other rheumatoid arthritis with rheumatoid factor of left wrist
M05.839	Other rheumatoid arthritis with rheumatoid factor of unspecified wrist
M05.841	Other rheumatoid arthritis with rheumatoid factor of right hand
M05.842	Other rheumatoid arthritis with rheumatoid factor of left hand
M05.849	Other rheumatoid arthritis with rheumatoid factor of unspecified hand
M05.851	Other rheumatoid arthritis with rheumatoid factor of right hip
M05.852	Other rheumatoid arthritis with rheumatoid factor of left hip
M05.859	Other rheumatoid arthritis with rheumatoid factor of unspecified hip

ICD Code	Description
M05.861	Other rheumatoid arthritis with rheumatoid factor of right knee
M05.862	Other rheumatoid arthritis with rheumatoid factor of left knee
M05.869	Other rheumatoid arthritis with rheumatoid factor of unspecified knee
M05.871	Other rheumatoid arthritis with rheumatoid factor of right ankle/foot
M05.872	Other rheumatoid arthritis with rheumatoid factor of left ankle/foot
M05.879	Other rheumatoid arthritis with rheumatoid factor of unspecified ankle/foot
M05.89	Other rheumatoid arthritis with rheumatoid factor multiple sites
M05.8A	Other rheumatoid arthritis with rheumatoid factor of other specified site
M05.9	Seropositive rheumatoid arthritis, unspecified
M06.00	Rheumatoid arthritis without rheumatoid factor, unspecified site
M06.011	Rheumatoid arthritis with out rheumatoid factor, right shoulder
M06.012	Rheumatoid arthritis with out rheumatoid factor, left shoulder
M06.019	Rheumatoid arthritis with out rheumatoid factor, unspecified shoulder
M06.021	Rheumatoid arthritis without rheumatoid factor, right elbow
M06.022	Rheumatoid arthritis without rheumatoid factor, left elbow
M06.029	Rheumatoid arthritis without rheumatoid factor, unspecified elbow
M06.031	Rheumatoid arthritis without rheumatoid factor, right wrist
M06.032	Rheumatoid arthritis without rheumatoid factor, left wrist
M06.039	Rheumatoid arthritis without rheumatoid factor, unspecified wrist
M06.041	Rheumatoid arthritis without rheumatoid factor, right hand
M06.042	Rheumatoid arthritis without rheumatoid factor, left hand
M06.049	Rheumatoid arthritis without rheumatoid factor, unspecified hand
M06.051	Rheumatoid arthritis without rheumatoid factor, right hip
M06.052	Rheumatoid arthritis without rheumatoid factor, left hip
M06.059	Rheumatoid arthritis without rheumatoid factor, unspecified hip
M06.061	Rheumatoid arthritis without rheumatoid factor, right knee

ICD Code	Description
M06.062	Rheumatoid arthritis without rheumatoid factor, left knee
M06.069	Rheumatoid arthritis without rheumatoid factor, unspecified knee
M06.071	Rheumatoid arthritis with out rheumatoid factor, right ankle/feet
M06.072	Rheumatoid arthritis with out rheumatoid factor, left ankle/feet
M06.079	Rheumatoid arthritis with out rheumatoid factor, unspecified ankle/feet
M06.08	Rheumatoid arthritis without rheumatoid factor, vertebrae
M06.09	Rheumatoid arthritis with out rheumatoid factor, multiple sites
M06.0A	Rheumatoid arthritis without rheumatoid factor,other site
M06.1	Adult-onset Still disease
M06.20	Rheumatoid bursitis, unspecified site
M06.211	Rheumatoid bursitis, right shoulder
M06.212	Rheumatoid bursitis, left shoulder
M06.219	Rheumatoid bursitis, unspecified shoulder
M06.221	Rheumatoid bursitis, right elbow
M06.222	Rheumatoid bursitis, left elbow
M06.229	Rheumatoid bursitis, unspecified elbow
M06.231	Rheumatoid bursitis, right wrist
M06.232	Rheumatoid bursitis, left wrist
M06.239	Rheumatoid bursitis, unspecified wrist
M06.241	Rheumatoid bursitis, right hand
M06.242	Rheumatoid bursitis, left hand
M06.249	Rheumatoid bursitis, unspecified hand
M06.251	Rheumatoid bursitis, right hip
M06.252	Rheumatoid bursitis, left hip
M06.259	Rheumatoid bursitis, unspecified hip
M06.261	Rheumatoid bursitis, right knee

ICD Code	Description
M06.262	Rheumatoid bursitis, left knee
M06.269	Rheumatoid bursitis, unspecified knee
M06.271	Rheumatoid bursitis, right ankle and foot
M06.272	Rheumatoid bursitis, left ankle and foot
M06.279	Rheumatoid bursitis, unspecified ankle and foot
M06.28	Rheumatoid bursitis, vertebrae
M06.29	Rheumatoid bursitis, multiple sites
M06.30	Rheumatoid nodule, unspecified site
M06.311	Rheumatoid nodule, right shoulder
M06.312	Rheumatoid nodule, left shoulder
M06.319	Rheumatoid nodule, unspecified shoulder
M06.321	Rheumatoid nodule, right elbow
M06.322	Rheumatoid nodule, left elbow
M06.329	Rheumatoid nodule, unspecified elbow
M06.331	Rheumatoid nodule, right wrist
M06.332	Rheumatoid nodule, left wrist
M06.339	Rheumatoid nodule, unspecified wrist
M06.341	Rheumatoid nodule, right hand
M06.342	Rheumatoid nodule, left hand
M06.349	Rheumatoid nodule, unspecified hand
M06.351	Rheumatoid nodule, right hip
M06.352	Rheumatoid nodule, left hip
M06.359	Rheumatoid nodule, unspecified hip
M06.361	Rheumatoid nodule, right knee
M06.362	Rheumatoid nodule, left knee
M06.369	Rheumatoid nodule, unspecified knee

ICD Code	Description
M06.371	Rheumatoid nodule, right ankle and foot
M06.372	Rheumatoid nodule, left ankle and foot
M06.379	Rheumatoid nodule, unspecified ankle and foot
M06.38	Rheumatoid nodule, vertebrae
M06.39	Rheumatoid nodule, multiple sites
M06.4	Inflammmatory polyarthropathy
M06.80	Other specified rheumatoid arthritis, unspecified site
M06.811	Other specified rheumatoid arthritis, right shoulder
M06.812	Other specified rheumatoid arthritis, left shoulder
M06.819	Other specified rheumatoid arthritis, unspecified shoulder
M06.821	Other specified rheumatoid arthritis, right elbow
M06.822	Other specified rheumatoid arthritis, left elbow
M06.829	Other specified rheumatoid arthritis, unspecified elbow
M06.831	Other specified rheumatoid arthritis, right wrist
M06.832	Other specified rheumatoid arthritis, left wrist
M06.839	Other specified rheumatoid arthritis, unspecified wrist
M06.841	Other specified rheumatoid arthritis, right hand
M06.842	Other specified rheumatoid arthritis, left hand
M06.849	Other specified rheumatoid arthritis, unspecified hand
M06.851	Other specified rheumatoid arthritis, right hip
M06.852	Other specified rheumatoid arthritis, left hip
M06.859	Other specified rheumatoid arthritis, unspecified hip
M06.861	Other specified rheumatoid arthritis, right knee
M06.862	Other specified rheumatoid arthritis, left knee
M06.869	Other specified rheumatoid arthritis, unspecified knee
M06.9	Rheumatoid arthritis, unspecified

ICD Code	Description
M08.00	Unspecified juvenile rheumatoid arthritis of unspecified site
M08.011	Unspecified juvenile rheumatoid arthritis, right shoulder
M08.012	Unspecified juvenile rheumatoid arthritis, left shoulder
M08.019	Unspecified juvenile rheumatoid arthritis, unspecified shoulder
M08.021	Unspecified juvenile rheumatoid arthritis, right elbow
M08.022	Unspecified juvenile rheumatoid arthritis, left elbow
M08.029	Unspecified juvenile rheumatoid arthritis, unspecified elbow
M08.031	Unspecified juvenile rheumatoid arthritis, right wrist
M08.032	Unspecified juvenile rheumatoid arthritis, left wrist
M08.039	Unspecified juvenile rheumatoid arthritis, unspecified wrist
M08.041	Unspecified juvenile rheumatoid arthritis, right hand
M08.042	Unspecified juvenile rheumatoid arthritis, left hand
M08.049	Unspecified juvenile rheumatoid arthritis, unspecified hand
M08.051	Unspecified juvenile rheumatoid arthritis, right hip
M08.052	Unspecified juvenile rheumatoid arthritis, left hip
M08.059	Unspecified juvenile rheumatoid arthritis, unspecified hip
M08.061	Unspecified juvenile rheumatoid arthritis, right knee
M08.062	Unspecified juvenile rheumatoid arthritis, left knee
M08.069	Unspecified juvenile rheumatoid arthritis, unspecified knee
M08.071	Unspecified juvenile rheumatoid arthritis, right ankle and foot
M08.072	Unspecified juvenile rheumatoid arthritis, left ankle and foot
M08.079	Unspecified juvenile rheumatoid arthritis, Unspecified ankle and foot
M08.08	Unspecified juvenile rheumatoid arthritis, vertebrae
M08.09	Unspecified juvenile rheumatoid arthritis, multiple sites
M08.0A	Unspecified juvenile rheumatoid arthritis, other site
M08.1	Juvenile ankylosing spondylitis

ICD Code	Description
M08.20	Juvenile rheumatoid arthritis with systemic onset of unspecified site
M08.211	Juvenile rheumatoid arthritis with systemic onset, right shoulder
M08.212	Juvenile rheumatoid arthritis with systemic onset, left shoulder
M08.219	Juvenile rheumatoid arthritis with systemic onset, unspecified shoulder
M08.221	Juvenile rheumatoid arthritis with systemic onset, right elbow
M08.222	Juvenile rheumatoid arthritis with systemic onset, left elbow
M08.229	Juvenile rheumatoid arthritis with systemic onset, unspecified elbow
M08.231	Juvenile rheumatoid arthritis with systemic onset, right wrist
M08.232	Juvenile rheumatoid arthritis with systemic onset, left wrist
M08.239	Juvenile rheumatoid arthritis with systemic onset, unspecified wrist
M08.241	Juvenile rheumatoid arthritis with systemic onset, right hand
M08.242	Juvenile rheumatoid arthritis with systemic onset, left hand
M08.249	Juvenile rheumatoid arthritis with systemic onset, unspecified hand
M08.251	Juvenile rheumatoid arthritis with systemic onset, right hip
M08.252	Juvenile rheumatoid arthritis with systemic onset, left hip
M08.259	Juvenile rheumatoid arthritis with systemic onset, unspecified hip
M08.261	Juvenile rheumatoid arthritis with systemic onset, right knee
M08.262	Juvenile rheumatoid arthritis with systemic onset, left knee
M08.269	Juvenile rheumatoid arthritis with systemic onset, unspecified knee
M08.271	Juvenile rheumatoid arthritis with systemic onset, right ankle and foot
M08.272	Juvenile rheumatoid arthritis with systemic onset, left ankle and foot
M08.279	Juvenile rheumatoid arthritis with systemic onset, Unspecified ankle and foot
M08.28	Juvenile rheumatoid arthritis with systemic onset, vertebrae
M08.29	Juvenile rheumatoid arthritis with systemic onset, multiple sites
M08.2A	Juvenile rheumatoid arthritis with systemic onset, other site
M08.3	Juvenile polyarthritis (seronegative)

ICD Code	Description
M08.40	Pauciarticular juvenile rheumatoid arthritis of unspecified site
M08.411	Pauciarticular juvenile rheumatoid arthritis, right shoulder
M08.412	Pauciarticular juvenile rheumatoid arthritis, left shoulder
M08.419	Pauciarticular juvenile rheumatoid arthritis, unspecified shoulder
M08.421	Pauciarticular juvenile rheumatoid arthritis, right elbow
M08.422	Pauciarticular juvenile rheumatoid arthritis, left elbow
M08.429	Pauciarticular juvenile rheumatoid arthritis, unspecified elbow
M08.431	Pauciarticular juvenile rheumatoid arthritis, right wrist
M08.432	Pauciarticular juvenile rheumatoid arthritis, left wrist
M08.439	Pauciarticular juvenile rheumatoid arthritis, unspecified wrist
M08.441	Pauciarticular juvenile rheumatoid arthritis, right hand
M08.442	Pauciarticular juvenile rheumatoid arthritis, left hand
M08.449	Pauciarticular juvenile rheumatoid arthritis, unspecified hand
M08.451	Pauciarticular juvenile rheumatoid arthritis, right hip
M08.452	Pauciarticular juvenile rheumatoid arthritis, left hip
M08.459	Pauciarticular juvenile rheumatoid arthritis, unspecified hip
M08.461	Pauciarticular juvenile rheumatoid arthritis, right knee
M08.462	Pauciarticular juvenile rheumatoid arthritis, left knee
M08.469	Pauciarticular juvenile rheumatoid arthritis, unspecified knee
M08.471	Pauciarticular juvenile rheumatoid arthritis, right ankle and foot
M08.472	Pauciarticular juvenile rheumatoid arthritis, left ankle and foot
M08.479	Pauciarticular juvenile rheumatoid arthritis, Unspecified ankle and foot
M08.48	Pauciarticular juvenile rheumatoid arthritis, vertebrae
M08.4A	Pauciarticular juvenile rheumatoid arthritis, other site
M08.80	Other juvenile arthritis of unspecified site
M08.811	Other juvenile arthritis, right shoulder

ICD Code	Description
M08.812	Other juvenile arthritis, left shoulder
M08.819	Other juvenile arthritis, unspecified shoulder
M08.821	Other juvenile arthritis, right elbow
M08.822	Other juvenile arthritis, left elbow
M08.829	Other juvenile arthritis, unspecified elbow
M08.831	Other juvenile arthritis, right wrist
M08.832	Other juvenile arthritis, left wrist
M08.839	Other juvenile arthritis, unspecified wrist
M08.841	Other juvenile arthritis, right hand
M08.842	Other juvenile arthritis, left hand
M08.849	Other juvenile arthritis, unspecified hand
M08.851	Other juvenile arthritis, right hip
M08.852	Other juvenile arthritis, left hip
M08.859	Other juvenile arthritis, unspecified hip
M08.861	Other juvenile arthritis, right knee
M08.862	Other juvenile arthritis, left knee
M08.869	Other juvenile arthritis, unspecified knee
M08.871	Other juvenile arthritis, right ankle and foot
M08.872	Other juvenile arthritis, left ankle and foot
M08.879	Other juvenile arthritis, Unspecified ankle and foot
M08.88	Other juvenile arthritis, other specifed site
M08.89	Other juvenile arthritis, multiple sites
M08.90	juvenile arthritis, unspecified of unspecified site
M08.911	juvenile arthritis, unspecified, right shoulder
M08.912	juvenile arthritis, unspecified, left shoulder
M08.919	juvenile arthritis, unspecified, unspecified shoulder

ICD Code	Description
M08.921	juvenile arthritis, unspecified, right elbow
M08.922	juvenile arthritis, unspecified, left elbow
M08.929	juvenile arthritis, unspecified, unspecified elbow
M08.931	juvenile arthritis, unspecified, right wrist
M08.932	juvenile arthritis, unspecified, left wrist
M08.939	juvenile arthritis, unspecified, unspecified wrist
M08.941	juvenile arthritis, unspecified, right hand
M08.942	juvenile arthritis, unspecified, left hand
M08.949	juvenile arthritis, unspecified, unspecified hand
M08.951	juvenile arthritis, unspecified, right hip
M08.952	juvenile arthritis, unspecified, left hip
M08.959	juvenile arthritis, unspecified, unspecified hip
M08.961	juvenile arthritis, unspecified, right knee
M08.962	juvenile arthritis, unspecified, left knee
M08.969	juvenile arthritis, unspecified, unspecified knee
M08.971	juvenile arthritis, unspecified, right ankle and foot
M08.972	juvenile arthritis, unspecified, left ankle and foot
M08.979	juvenile arthritis, unspecified, Unspecified ankle and foot
M08.98	juvenile arthritis, unspecified, vertebrae
M08.99	juvenile arthritis, unspecified, multiple sites
M08.9A	juvenile arthritis, unspecified, other specified site
M09.8	Juvenile arthritis in other diseases classified elsewhere
M31.30	Wegener's granulomatosis without renal involvement
M31.31	Wegener's granulomatosis with renal involvement
M33.00	Juvenile dermatomyositis, organ involvement unspecified
M33.01	Juvenile dermatomyositis with respiratory involvement

ICD Code	Description
M33.02	Juvenile dermatomyositis with myopathy
M33.03	Juvenile dermatomyositis without myopathy
M33.09	Juvenile dermatomyositis with other organ involvement
M33.10	Other dermatomyositis, organ involvement unspecified
M33.11	Other dermatomyositis with respiratory involvement
M33.12	Other dermatomyositis with myopathy
M33.13	Other dermatomyositis without myopathy
M33.19	Other dermatomyositis with other organ involvement
M33.20	Polymyositis, organ involvement unspecified
M33.21	Polymyositis with respiratory involvement
M33.22	Polymyositis with myopathy
M33.29	Polymyositis with other organ involvement
M33.90	Dermatopolymyositis, unspecified, organ involvement unspecified
M33.91	Dermatopolymyositis, unspecified with respiratory involvement
M33.92	Dermatopolymyositis, unspecified with myopathy
M33.93	Dermatopolymyositis, unspecified without myopathy
M33.99	Dermatopolymyositis, unspecified with other organ involvement
M31.5	Giant cell arteritis with polymyalgia rheumatica
M31.6	Other giant cell arteritis
M35.3	Polymyalgia rheumatica
G70.00	Myasthenia gravis without (acute) exacerbation
G70.01	Myasthenia gravis with (acute) exacerbation
M34.0	progressive systemic sclerosis
M34.1	CREST syndrome
M34.81	Systemic sclerosis with lung involvement
M34.82	Systemic sclerosis with myopathy

ICD Code	Description
M34.83	Systemic sclerosis with polyneuropathy
M34.89	Other systemic sclerosis
M34.9	Systemic sclerosis, unspecified
M32.10	Systemic lupus erythematosus, organ or systemic involvement unspecified
M32.11	Endocarditis in systemic lupus erythematosus
M32.12	Pericarditis in systemic lupus erythematosus
M32.13	Lung involvement in systemic lupus erythematosus
M32.14	Glomerular disease in systemic lupus erythematosus
M32.15	Tubulo-interstitial neuropath in systemic lupus erythematosus
M32.19	Other organ or system involvement in systemic lupus erythematosus
M32.8	Other forms of systemic lupus erythematosus
M32.9	Systemic lupus erythematosus, unspecified
M35.00	Sjögren syndrome, unspecified
M35.01	Sjögren syndrome with keratoconjunctivitis
M35.02	Sjögren syndrome with lung involvement
M35.03	Sjögren syndrome with myopathy
M35.04	Sjögren syndrome with tubulo-interstitial nephropathy
M35.05	Sjögren syndrome with inflammatory arthritis
M35.06	Sjögren syndrome with peripheral nervous system involvement
M35.07	Sjögren syndrome with central nervous system involvement
M35.08	Sjögren syndrome with gastrointestinal involvement
M35.09	Sjögren syndrome with other organ involvement
M35.0A	Sjögren syndrome with glomerular disease
M35.0B	Sjögren syndrome with vasculitis
M35.0C	Sjögren syndrome with dental involvement
M45.0	Ankylosing spondylitis of multiple sites in spine

ICD Code	Description
M45.1	Ankylosing spondylitis of occipito-atlanto-axial region
M45.2	Ankylosing spondylitis of cervical region
M45.3	Ankylosing spondylitis of cervicothoracic region
M45.4	Ankylosing spondylitis of thoracic region
M45.5	Ankylosing spondylitis of thoracolumbar region
M45.6	Ankylosing spondylitis lumbar region
M45.7	Ankylosing spondylitis of lumbosacral region
M45.8	Ankylosing spondylitis sacral and sacrococcygeal region
M45.9	Ankylosing spondylitis of unspecified sites in spine
M45.A0	Non-radiographic axial spondyloarthritis of unspecified sites in spine
M45.A1	Non-radiographic axial spondyloarthritis of occipito-atlanto-axial region
M45.A2	Non-radiographic axial spondyloarthritis of cervical region
M45.A3	Non-radiographic axial spondyloarthritis of cervicothoracic region
M45.A4	Non-radiographic axial spondyloarthritis of thoracic region
M45.A5	Non-radiographic axial spondyloarthritis of thoracolumbar region
M45.A6	Non-radiographic axial spondyloarthritis of lumbar region
M45.A7	Non-radiographic axial spondyloarthritis of lumbosacral region
M45.A8	Non-radiographic axial spondyloarthritis of sacral and sacrococcygeal region
M45.AB	Non-radiographic axial spondyloarthritis of multiple sites in spine
M35.2	Behcet's disease
D86.0	Sarcoidosis of lung
D86.1	Sarcoidosis of lymph nodes
D86.2	Sarcoidosis of lung with sarcoidosis of lymph nodes
D86.3	Sarcoidosis of skin
D86.81	Sarcoid meningitis
D86.82	Multiple cranial nerve palsies in sarcoidosis

ICD Code	Description
D86.83	Sarcoid iridocyclitis
D86.84	Sarcoid pyelonephritis
D86.85	Sarcoid myocarditis
D86.86	Sarcoid arthropathy
D86.87	Sarcoid myositis
D86.89	Sarcoidosis of other sites
D86.9	Sarcoidosis, unspecified
M63.3	Myositis in sarcoidosis
G53.2	Multiple cranial nerve palsies in sarcoidosis

Supplementary Table 23: Autoimmune ICD-10 Codes. From Tunnicliffe et al. [3].

ICD-10 Code	Diagnosis Type
K50	Inflammatory Bowel Disease (IBD)
K50.0	Inflammatory Bowel Disease (IBD)
K50.1	Inflammatory Bowel Disease (IBD)
K50.8	Inflammatory Bowel Disease (IBD)
K50.9	Inflammatory Bowel Disease (IBD)
K51	Inflammatory Bowel Disease (IBD)
K51.0	Inflammatory Bowel Disease (IBD)
K51.2	Inflammatory Bowel Disease (IBD)
K51.3	Inflammatory Bowel Disease (IBD)
K51.4	Inflammatory Bowel Disease (IBD)
K51.5	Inflammatory Bowel Disease (IBD)
K51.8	Inflammatory Bowel Disease (IBD)
K51.9	Inflammatory Bowel Disease (IBD)

ICD-10 Code	Diagnosis Type
M45	Inflammatory Arthropathy
M46	Inflammatory Arthropathy
M46.0	Inflammatory Arthropathy
M46.2	Inflammatory Arthropathy
M46.3	Inflammatory Arthropathy
M46.4	Inflammatory Arthropathy
M46.5	Inflammatory Arthropathy
M46.8	Inflammatory Arthropathy
M46.9	Inflammatory Arthropathy
M05	Seropositive Rheumatoid Arthritis
M05.0	Seropositive Rheumatoid Arthritis
M05.1	Seropositive Rheumatoid Arthritis
M05.2	Seropositive Rheumatoid Arthritis
M05.3	Seropositive Rheumatoid Arthritis
M05.8	Seropositive Rheumatoid Arthritis
M05.9	Seropositive Rheumatoid Arthritis
M06	Other Rheumatoid Arthritis
M06.0	Other Rheumatoid Arthritis
M06.1	Other Rheumatoid Arthritis

**ICD-10 Code**	**Diagnosis Type**
M06.2	Other Rheumatoid Arthritis
M06.3	Other Rheumatoid Arthritis
M06.4	Other Rheumatoid Arthritis
M06.8	Other Rheumatoid Arthritis
M06.9	Other Rheumatoid Arthritis
M07	Psoriatic Arthritis
M07.0	Psoriatic Arthritis
M07.1	Psoriatic Arthritis
M07.2	Psoriatic Arthritis
M07.3	Psoriatic Arthritis
M07.4	Psoriatic Arthritis
M07.5	Psoriatic Arthritis
M07.6	Psoriatic Arthritis
L40	Psoriasis
L40.0	Psoriasis
L40.1	Psoriasis
L40.2	Psoriasis
L40.3	Psoriasis
L40.4	Psoriasis
L40.5	Psoriasis
L40.8	Psoriasis
L40.9	Psoriasis

Supplementary Table 24: IMID Diagnosis Codes. From Attauabi et al. [4]

References

[1] Mansfield, K. E. *et al.* Clinical Codelist - Immunosuppression - ICD-10 Codes (2019). URL http://datacompass.lshtm.ac.uk/id/eprint/1276.

[2] Mansfield, K. E. *et al.* Clinical Codelist - Diabetes Mellitus Diagnostic - ICD-10 Codes (2019). URL http://datacompass.lshtm.ac.uk/id/eprint/1273.

[3] Tunnicliffe, L. & Warren-Gash, C. Clinical codelist- autoimmune disease ICD10 codes (2022). URL https://datacompass.lshtm.ac.uk/id/eprint/2853.

[4] Attauabi, M. *et al.* Coronavirus disease 2019, immune-mediated inflammatory diseases and immunosuppressive therapies – A Danish population-based cohort study. *Journal of Autoimmunity* **118**, 102613 (2021). URL https://linkinghub.elsevier.com/retrieve/pii/S0896841121000214.
