# Supplementary material for: Large-scale genomic surveillance reveals immunosuppression drives mutation dynamics in persistent SARS-CoV-2 infections
Source: Nat Commun. 2026 Jun 19;17:7730. doi: 10.1038/s41467-026-74445-7 (PMC13433919; doi:10.1038/s41467-026-74445-7)
Supplement: Supplementary file 2 [file 41467_2026_74445_MOESM2_ESM.pdf]

nature portfolio

Peer Review File

Large-scale genomic surveillance reveals immunosuppression drives mutation dynamics in persistent SARS-CoV-2 infections

Corresponding Author: Dr Mark Khurana

This file contains all reviewer reports in order by version, followed by all author rebuttals in order by version.

Attachments originally included by the reviewers as part of their assessment can be found at the end of this file.

Version 0:

Reviewer comments:

Reviewer #1

(Remarks to the Author)
Comments are attached

(Remarks on code availability)
The code looks to be complete and reproducible.

Reviewer #2

(Remarks to the Author)
In the manuscript by Khurana et al. the authors use Denmark's COVID Genome Consortium that links viral genomic data with patient metadata to identify persistent infections and identify potential risk factors. Based on their data selection the authors find a total of 303 persistent infections and identify immunosuppression as the major risk factor of developing a persistent SARS-CoV-2 infection. The study is largely modelled after a previously published study in Nature by Ghafari et al. with a similar study cohort but with the advantage that much more patient metadata was included in the present study. I would like to acknowledge that the study is quite extensive, the statistical approaches are sound and the study overall extends our current knowledge of persistent SARS-CoV-2 infections. However, some points have to be addressed before I can recommend publication in Nature Communications:
- Some figures are not referenced in the text and are in non-consecutive order making it hard to read the manuscript. I highly encourage that the authors re-check whether all figures are referenced correctly. Some inconsistencies that I found include:
o Figure 1 A-E: Not specifically mentioned in the text.
o Suppl. Table 3: Mentioned after Suppl. Table 4.
o Figure 2 and corresponding text: Figure 2C is mentioned before Figure 2B. Figure 2D and F are mentioned after Figure 4 and Figure 2 E is never mentioned at all.
o Suppl. Table 10: mentioned after Suppl. Table 11

- Some assumptions are not well explained why they were set:
o Line 116: The rare SNP threshold of 10.9% is chosen using a simulation of random sequence pairs and appears to balance the number of persistent infections against a 2–3% false positive rate. However, the exact rationale for selecting 10.9% is not fully explained.
o Line 109: Ct 38 as a threshold for sequencing quality is unclear. Ct threshold are highly qPCR dependent (as they stem from different laboratories) and not a clear indicator for sequence quality. Genome coverage (such as number of Ns/non-covered regions) is a more accepted standard for consensus sequence quality. The authors should clarify and officially reference DCGC guidelines if publicly available. In the methods the authors mentioned QC for generating sequencing data. Were all sequences generated the same way? If yes, the authors should state this in the main text rather than the Ct threshold to avoid confusion.
o Line 131: What was the reason for the time cutoff for no additional tests in the control group within 120 days? Should a control not also be included if a test has been performed and the sequences are divergent enough?

o Line 212: Why restrict the analyses to sequences with at least 1 non-syn change. Why not just use all 'final' sequences?

- Line 140 and following: While the higher risk of prolonged infections in immunocompromised individuals is a quite well-established fact, the reasoning to only analyse pre-2020 diagnoses is unclear to me. To identify it as a risk factor, the immunosuppression should be present at the time of infection. The added value of the contemporary analysis is not clear to me, since it ends (31st of December 2021) before many of the later persistent episodes; please clarify what additional information it provides.
- Line 168 following: The authors show cumulative number of mutational changes over all identified samples. While this is sufficient to give an overall overview over the number of syn to non-syn mutations, Figure 2A and the corresponding text could be misleading. The number of changes per variant is misleading as the number of mutations per site depends on the time difference between consecutive samples. The authors indeed show differences in the infection time (Figure 1 B). More changes for BA.2 could simply be attributed to the sampling timeframe. Notably, this is not stated in the text, but the figure implies it.
- Line 170 following: Here the sampling timeframe is also not considered. If I am not mistaken, the raw cumulative mutation counts were dived by the length of the individual genes. The authors should consider the time difference between consecutive samples and evaluate this per sequence pair to get a real estimate of a mutation rate per gene. Then the authors can test whether the presented differences are statistically significant.
- Supplementary Figure 2: The authors distinguish Supplementary Fig. 2 ('all pairs of consecutive sequences') from Fig. 2B ('first vs last sequence only'), but visually the patterns look very similar, and it's not obvious what additional biological insight is gained by showing both. It might be worth clarifying the motivation for keeping both panels.
- Line 183 onward: Currently the authors only test whether there are differences between both groups in their Ka/Ks differences per gene. However, the approach cannot detect site specific adaptations. Positive selection of individuals with immunosuppression should correlate with some sort of selection pressure. Therefore, the manuscript would benefit from site-specific models. It would be highly interesting if the authors can correlate positive selected sites with the observed mutations that likely confer resistance to antiviral treatment.
- Line 313 and following: (I) Which COVID-19 database are the authors referring to? (II) The matching analysis compares final sequences of persistent infections to other sequences within an 11 day window. While the authors show that many of these sequences have zero, one, or two differences this is difficult to interpret without a proper baseline: with dense sequencing, exact or near exact matches are expected. Without explicit reconstruction of transmission clusters, the evidence for onward transmission remains suggestive.
- Line 247 and following: Are higher number of non-syn mutations special to long-term infections or something that is variant specific? Can the authors clarify with their model if the increase in the number of non-syn changes between the alpha and remaining variants is something that is also mimicked when analysing the global pyhlogeny?
- Line 265 and following: That the number of mutations in older age groups is lower than in younger age groups is intriguing. The authors give immune senescence as a potential reason. However, their identified risk factor is immunosuppression. Should the viral evolution in all cases then not be independent of immune pressure? This data is not easy to explain biologically and might need a more detailed discussion.
- Line 296: Antibodies and antivirals were only in use during specific timeframes. In the absence of data for antiviral treatment it would be interesting if the timeframes match, e.g. Molnupiravir resistances in an Alpha variant would not make sense. Can the authors match resistance mutations and sampling times to drug availability?

(Remarks on code availability)

Reviewer #3

(Remarks to the Author)
Thank you for the opportunity to review this manuscript. Persistent SARS-CoV-2 infections are thought to play an important role in the emergence of novel viral variants, and the study by Khurana et al. provides valuable large-scale data to further investigate these processes. While several findings are consistent with prior reports, such as the strong association between persistent infection and immunocompromising conditions, the size and scope of the cohort are unique and benefits from patient registry information. My main comments focus on methodological choices related to the definition of persistent infection, sequencing platforms, and control selection, as detailed below.

Major comments

1) Sequence pairs within the same variant sampled at least 26 days apart are considered indicative of potentially persistent infections. This approach may exclude true persistent infections that undergo substantial sequence evolution resulting in clade or variant misclassification over time.

2) There is potential for sequencing errors associated with the MinION platform, particularly for R9.4.1 flow cells. Given the use of multiple sequencing platforms, it would be helpful to clarify whether any persistent infection cases had samples sequenced on different platforms, and if so, how platform-specific error profiles were accounted for.

3) Separating persistent infection from reinfection is challenging. Is information on intervening negative tests available, or are persistent infections identified solely as uninterrupted runs of positive tests? The likelihood of reinfection increases with longer time intervals, and this should be addressed more explicitly.

4) Persistent infections appear to be defined, in part, by the presence of shared rare SNPs across consecutive time points relative to the background population for a given major variant. This approach could be problematic because persistent infections can undergo rapid intra-host evolution, potentially reducing the number of shared SNPs over time, particularly in cases with sparse sampling. While the approach is stringent and helps ensure that sequences represent the same infection, it may bias the analysis toward a specific subset of cases, especially when characterizing the mutational landscape and onward transmission potential of persistent infections.

5) Controls are defined as individuals with a negative test within 26 days of a positive result and no additional positive tests within the subsequent 120 days. However, the ability to detect persistent infection depends strongly on testing frequency. The authors should assess whether testing rates (positive and negative) are comparable between cases and controls, and consider further stratifying controls to include individuals with similar testing intensity but no evidence of persistent infection.

6) A high proportion of elderly patients is observed among the identified persistent infection cases. This may reflect slower viral clearance and prolonged PCR positivity in older individuals rather than true persistence. Variable recovery times by age should be considered and discussed.

7) The onward transmission analysis is difficult to interpret. Because the selection criteria for persistent infection cases enrich for sequences containing rare SNPs, it is unclear how likely it is to observe the reported number of similar sequences by chance alone in the dataset. Is it possible to formally assess this likelihood? An orthogonal approach, such as assessing whether related sequences are also geographically clustered, would also strengthen the conclusions if such data are available.

Minor comments

1. While analyzing spike mutations as a whole is reasonable, it would be helpful to provide more detailed information on mutations within key antigenic regions, particularly the N-terminal domain (NTD) and receptor-binding domain (RBD).

2. No minor intra-host variant data are presented. Many mutations may exist at sub-consensus frequencies and therefore would not be detected in consensus sequences. This limitation should be acknowledged explicitly.

3. While I understand the complexities of dealing with individual-level data in large cohorts, I wonder if at least the sequence data for persistent infection cases could be made available with limited metadata.

(Remarks on code availability)
While the authors have done a commendable job of making the analysis code available, the lack of underlying data complicates reproducibility. I understand the complications in making individual-level data available but there are analyses presented in the manuscript that do not rely on individualized data. For other analyses, pre-processed aggregate data could be provided instead.

Reviewer #4

(Remarks to the Author)
The authors describe their work analyzing the extensive SARS-CoV-2 genomic surveillance data from Denmark (>700,000 genomes) to identify and characterize persistent infections (n=303). The authors were comprehensive in their extensive analyses and convincing in their efforts to connect a correlative association of immunosuppression as a driver of mutation dynamics in those individuals with persistent SARS-CoV-2 infection.
The authors show that older individuals had higher odds of persistence than younger groups and show that those who are immunocompromised were at highest risk of developing persistent infections relative to other co-morbidity states (over 5 times the risk, line 322). A strong point in their analysis is that they conducted matched case-control study for each persistent infection to examine possible risk factors for persistent infection.

The authors provide data to support the idea that immunosuppression coupled with SARS-CoV-2 monoclonal (mAB) or antiviral treatment was associated with significantly higher numbers of nonsynonymous mutations (line 282), particularly mutations associated with mAB resistance. Although specific link to actual treatment therapies was not provided but rather inferred from the data (immunosuppression and/or immunocompromised diagnosis), the authors were correct to point out that while nonsynonymous mutations increased in immunosuppressed synonymous mutations did not, indicative of selective pressure (presumably from mAB/antiviral therapies). Notably, the authors point out that while older aged-groups had higher odds of persistent infection, they showed fewer within-host substitutions, both nonsynonymous and synonymous (lines 337-338). This paradox was attributed to immune senescence that would impair immune responses leading to prolonged infections with less selective pressure and therefore less viral diversity, as observed.

This study demonstrates the power of SARS-CoV-2 genomic surveillance data when integrated with clinical and epidemiologic data to understand population-level factors influencing viral evolution. The authors present a convincing set of analyses from Denmark’s comprehensive and unique datasets that links immunosuppression and presumed antiviral treatment as a driver of selective pressure to effect viral mutation dynamics.

Comments/questions:

• I wonder about the inclusion criterion of Ct values between 30 and 38 for whole genome sequencing. In my experience, it has been difficult to obtain high quality sequence data from samples with higher Ct values, particularly Ct >32. Can the authors please comment on this and explain how these higher Ct value samples yielded adequate sequence data for this study?

• I'm curious about the authors seeming to equate fitness of recurrent within-host nonsynonymous mutations with enhanced transmission between hosts as stated here:
Lines 355-358 paraphrased: Many "…recurrent nonsynonymous mutations within a host have a higher between-host fitness on average compared to single-occurring and unrealized mutations at the same sites."
Is this statement related to Figure 4? If so, can you help me understand this in the context of Figure 4? Furthermore, is this Please also note, that for me, Figure 4A colors in the graph do not correspond to colors in the legend – please enhance and reconcile this figure accordingly.
• Line 353: "Notably, genomes of individuals with immunosuppression harbored significantly more nonsynonymous changes upon infection"
Was this shown or discussed in the text prior to this line? Please elaborate – this statement seems to say that immunosuppressed individuals were infected by virus with more nonsynonymous mutations than non-immunosuppressed persons. Is this what you mean, that immunosuppressed persons start an infection with more mutated virus? If not, please update the wording to reflect what you mean.
• This study focused on consensus sequence data, but I'm curious if you were able to identify any subconsensus substitutions in earlier sequence that became fixed at later sampling time?
• Line 223 refers to Figure 3D – but the manuscript has no such figure.
• Lines 219 – 240 – describes the substitutions detected. As a favor to the reader, it would be quite helpful to include these data in a table.
• I found the following sentence (lines 300 – 304) a bit awkward – suggest rewording it for clarity:
"58.3% (7/12) of individuals with a monoclonal antibody resistance-linked substitution—specifically among those with infections ending prior to mid-2022, out of a total of 34 individuals (including those with infections ending beyond this point), which is the cut-off for available hospitalization data—were hospitalized at some point during their infection."
• Line 353: "Notably, genomes of individuals…" update this passage to include "viral" as follows:
"Notably, viral genomes of individuals…"

• Line 360: Suggest inserting "immunosuppressed" into the sentence as follows –
"…repeatedly within immunosuppressed hosts…"

• Line 426 – you mention not examining subconsensus sequence; suggest you look at a recent study by Bell, et al., who did a similar examination of persistent infections from a large data set but did examine subconsensus sequence data.
Bell JM, Elder J, Ryder R, Smith EA, Scribner M, Gilliam S, et al. (2025). Genomic and epidemiologic characteristics of SARS-CoV-2 persistent infections in California, January 2021 – July 2023. PLOS Pathogens, 21(11): e1013365. https://doi.org/10.1371/journal.ppat.1013365
• Line 496: curious how you decided on the <120 days apart metric?
• Lines 121-122 and 590-591: Ct values are correlated to viral load, but that is an incorrect assumption; suggest using another term instead of viral load…perhaps viral RNA load?
• Lines 653-654: data availability of consensus genomes is restricted due to what the authors say pertains to Danish data protection laws. This is counter to the SARS-CoV-2 global initiative to share at least sequence data for public access without compromising individual privacy. Does this mean that there are no Danish SARS-CoV-2 sequence data available publicly via GISAID or other public data repository?

(Remarks on code availability)
My computer systems do not allow me access to github sites so I could not review the code.

Version 1:

Reviewer comments:

Reviewer #1

(Remarks to the Author)
The authors have fully addressed all of my concerns. This is a great study!

(Remarks on code availability)
The code is well organized and is a usable resource for the community.

Reviewer #2

(Remarks to the Author)
I thank the authors for the updates and additions to the manuscript. My comments and questions have sufficiently been addressed.

I have also assessed the responses to Reviewer 3's comments. In my opinion, all comments were adequately addressed

with the exception of point 3:

The reviewer asked how negative tests influence the detection of long-term infection. It stands to reason that negative tests in between positive tests should be considered in the detection of long-term infection as this could also indicate reinfection. The authors state that they model rebounding frequency (positive test – negative test – positive test) to analyse if this is influenced by immunosuppression. However, this is not what the critique was targeting at. I actually agree that if information about negative tests is available, they should be considered in the initial definition of a long-term infection.

(Remarks on code availability)

Reviewer #4

(Remarks to the Author)
The authors have satisfactorily addressed my major concerns. However, the coloring of the histograms in Figures 2f, 3, and 4 remains an issue. The bars contain yellow or green gradients that are not reflected in the legend. To ensure clarity for the reader, I suggest the authors use consistent coloring that corresponds with the provided legend.

(Remarks on code availability)
My computer systems do not allow me access to github sites, so I am not able to review the code.

Version 2:

Reviewer comments:

Reviewer #2

(Remarks to the Author)
I think this clarifies Reviewer 3's concern regarding negative tests and have no further comments.

(Remarks on code availability)

Open Access This Peer Review File is licensed under a Creative Commons Attribution 4.0 International License, which permits use, sharing, adaptation, distribution and reproduction in any medium or format, as long as you give appropriate credit to the original author(s) and the source, provide a link to the Creative Commons license, and indicate if changes were made.
In cases where reviewers are anonymous, credit should be given to 'Anonymous Referee' and the source.
The images or other third party material in this Peer Review File are included in the article’s Creative Commons license,

unless indicated otherwise in a credit line to the material. If material is not included in the article's Creative Commons license and your intended use is not permitted by statutory regulation or exceeds the permitted use, you will need to obtain permission directly from the copyright holder.
To view a copy of this license, visit https://creativecommons.org/licenses/by/4.0/

Our team is grateful for the feedback from the reviewers on our manuscript entitled "Large-scale genomic surveillance reveals immunosuppression drives mutation dynamics in persistent SARS-CoV-2 infections". We believe that the manuscript has benefited substantially from these revisions, as described below, and we hope it is now suitable for publication in *Nature Communications*.

Reviewer 1

General comment: Khurana et al. leveraged Denmark's extensive genomic surveillance to identify persistent infections and used associated health and demographic factors as part of a case-control study to determine risk factors associated with persistent infections. They identified 303 persistent infections. Older adults, unvaccinated individuals, and immunosuppressed individuals were all at greater risk of persistent infections. They also detected positive selection in Spike, and recurrent mutations were in monoclonal antibody/antiviral resistance sites and sites detected in other immunocompromised individuals. It is critical to understand persistent SARS-CoV-2 infections and the risk factors for persistent infections because new variants and immune escape mutations appear to originate from persistent infections. Large genomic surveillance efforts with sufficient metadata are a great resource for understanding persistent infection, especially when combined with matching control cases. It is clear that a lot of work has gone into this study.

Comments	Author response	Location of change in new version
My biggest concern with this study is the definition of persistent infections used in this study. They used a very similar method to Ghafari et al. However, Ghafari et al. only used samples with a Ct value of ≤30, whereas this study used a cutoff of ≤38. High Ct samples are less likely to reflect an active infection. The higher Ct threshold is likely to result in the identification of more false persistent infections. How does the data compare if the Ct threshold was lowered? Are many of the persistent infections no longer identified? From Figure 1F, it looks like most of the last samples have Ct values greater than 30.	We thank the reviewer for this comment. We first would like to note that as previously reported (Michaelsen et al., Lyngse et al., and Khurana et al.), cycle threshold (Ct) cut-offs specified by the Danish Statens Serum Institut (SSI) and the Danish COVID-19 Genome Consortium (DCGC) were between 30 and 38. These were chosen to pass the SSI's stringent internal quality control for WGS; therefore, our definition is aligned with those used during the pandemic in Denmark. A bound of ≤ 38 represents a lower threshold than Ghafari et al.; the cutoff in Ghafari et al. was chosen because at higher Ct values sequencing success was less likely and was therefore a practical resource consideration in the UK. Therefore neither 30 or 38 is linked to, or guarantees an infection, but is a pragmatic choice more closely linked to sequencing. Nevertheless, the cutoff of 38 in Denmark is well-motivated:<br><br>Indeed, as noted by Lyngse et al.: "However, even for primary cases with relatively high	Lines 436-445

	sample Ct values, the transmissibility was not negligible, e.g., for primary cases with a sample Ct value of 38, we found that 13% of the primary cases had at least one secondary household case."<br><br>This methodology is also consistent with the consensus framework for persistent infection. Machkovech et al. in *The Lancet Infectious Diseases* propose to define persistent infection via several markers, including the presence of a 'accumulation of mutations over time in a single infecting viral lineage' over an extended period. Our approach of identifying this continuity via shared rare SNPs satisfies the core requirement of this definition. By demonstrating that the virus at the second time point is genetically linked to the first through rare SNPs, rather than a new infection from the population, we confirm persistence and the fact that it is a single lineage. Furthermore, the ability to generate a high-quality consensus genome at a second time point (even with a Ct up to 38) is itself evidence of a significant presence of intact viral RNA, which is biologically distinct from the degraded, low-level 'residual RNA shedding' that typically fails WGS.<br><br>Unfortunately, exact Ct values were also not available for the entire dataset provided by the SSI (i.e., they passed the quality control thresholds, but the specific Ct value was not always recorded in the final dataset). Implementing a post-hoc exclusion based on this would therefore introduce "missing not at random" biases, since it would be unknown whether those with available Ct values greater than the threshold systematically differ from those without. We have added a sentence in the Limitations section to make this more explicit.	
Matching last the sequence of persistent infections to genetically similar acute sequences isn't strong evidence of the plausibility of onward transmission. Within the 11 day time frame many epidemiologically unlinked but genetically similar acute infections are	We agree that genetic similarity within an 11-day window in a high-prevalence setting does not constitute proof of direct transmission. Our intention was not to claim definitive transmission events, but rather to observe whether persistent sequences remain within the genetic space of circulating variants. We	Line 192-200 (Results)<br><br>Line 409-411 (Discussi

present, so it isn't surprising that some persistent infections are very similar to what is circulating. Additionally, for the persistent samples that have 1 or 2 mismatches with acute samples, the mismatches may just be the initial sequence of the persistent infection. I am not arguing that onward transmission from persistent infections is not plausible, just that this analysis doesn't add to what we know.	have revised the text (both in the Results and in the Discussion) to tone down this claim, clarifying that these findings simply show that persistent infections remain genetically consistent with the broader viral population, which is a necessary, albeit not sufficient, condition for onward transmission. We have also added an extra Supplementary Table (including corresponding text in the main manuscript) to highlight that these samples were from similar regions, further strengthening the argument regarding plausible local transmission.	on)<br><br>Supplementary Table 6
Lines 122-124; Ct values aren't a direct indicator of sequence quality. It is hard to get high quality sequences from low viral load samples, but there are cases when you do, and many low Ct value samples don't result in high quality sequencing.	We completely agree that this is misleading and have removed the specific reference to sequence quality.	Line 124
You should cite Raglow et al. 2024 (https://www.thelancet.com/journals/lanmic/article/PIIS2666-5247(23)00336-1/fulltext?uuid=uuid%3A5679b141-e4e6-47fe-8378-759a73a7e84d). It is a cohort study of hospitalized immunocompromised individuals and the conditions to promote persistent infections.	We thank the reviewer for referring us to the article; we have now cited it in the manuscript.	Reference 7
Figure 1 AB and lines 251-256. The Ct range that was sequenced by DCGC varied over the course of the pandemic (maximum Ct 30-38). Could this influence the patterns when comparing the different variants?	We thank the reviewer for this point. The maximum Ct threshold for sequencing at the DCGC was indeed adjusted based on national sequencing capacity. However, we do not expect this to significantly confound the patterns observed across variants. Because the majority of the variants analyzed in this study co-circulated during this high-capacity window (https://www.nature.com/articles/s41467-024-51371-0/figures/2 & https://www.covid19genomics.dk/statistics), they were subject to very similar sequencing protocols. This overlap suggests that the differences observed between variants are driven by biological factors rather than shifting technical cutoffs or capacity constraints. We have noted this in the Limitations section to ensure transparency	Line 439-445 (Limitations)

	regarding the varying Ct thresholds.	

Reviewer 2
General comment: In the manuscript by Khurana et al. the authors use Denmark's COVID Genome Consortium that links viral genomic data with patient metadata to identify persistent infections and identify potential risk factors. Based on their data selection the authors find a total of 303 persistent infections and identify immunosuppression as the major risk factor of developing a persistent SARS-CoV-2 infection. The study is largely modelled after a previously published study in Nature by Ghafari et al. with a similar study cohort but with the advantage that much more patient metadata was included in the present study. I would like to acknowledge that the study is quite extensive, the statistical approaches are sound and the study overall extends our current knowledge of persistent SARS-CoV-2 infections.

Comments	Author response	Location of change
Some figures are not referenced in the text and are in non-consecutive order making it hard to read the manuscript. I highly encourage that the authors re-check whether all figures are referenced correctly. Some inconsistencies that I found include:<br>- Figure 1 A-E: Not specifically mentioned in the text.<br>- Suppl. Table 3: Mentioned after Suppl. Table 4.<br>- Figure 2 and corresponding text: Figure 2C is mentioned before Figure 2B. Figure 2D and F are mentioned after Figure 4 and Figure 2 E is never mentioned at all.<br>- Suppl. Table 10: mentioned after Suppl. Table 11	We thank the reviewer for pointing this out. We have now gone through the manuscript again to ensure that all figures are mentioned and that the order is correct. We also moved a portion of the text such that the discussion of the contents of Figure 2 are all discussed before Figures 3 and 4.	Figure 1, 2, and 4<br><br>All supplement ary figures
Line 116: The rare SNP threshold of 10.9% is chosen using a simulation of random sequence pairs and appears to balance the number of persistent infections against a 2–3% false positive rate. However, the exact rationale for selecting 10.9% is not fully explained.	We thank the reviewer for highlighting the need for further clarification on our threshold selection. The threshold of 10.9% (a number randomly generated using logarithmic spacing) was selected by optimizing the trade-off between sensitivity (identifying true persistent infections) and specificity (minimizing false positives arising from coincidental shared rare SNPs in the general population). The 10.9% threshold was chosen because it was the point at which the false-positive rate was below 2-3% (after which it rapidly increases; see Supplementary Figure 4). We have	Line 539-544

	updated the Methods to more explicitly describe the process.	
Line 109: Ct 38 as a threshold for sequencing quality is unclear. Ct threshold are highly qPCR dependent (as they stem from different laboratories) and not a clear indicator for sequence quality. Genome coverage (such as number of Ns/non-covered regions) is a more accepted standard for consensus sequence quality. The authors should clarify and officially reference DCGC guidelines if publicly available. In the methods the authors mentioned QC for generating sequencing data. Were all sequences generated the same way? If yes, the authors should state this in the main text rather than the Ct threshold to avoid confusion.	We completely agree that this is misleading and have removed the Ct reference to sequence quality.<br><br>As discussed in our response to Reviewer 1, the Ct 38 threshold aligns with Danish national standards (SSI/DCGC) and is biologically justified by evidence of transmissibility even at high Ct values (Lyngse et al., 2021). Within this framework, all included sequences passed stringent quality control (QC) criteria: <3000 ambiguous bases (Ns), ≤ 5 ambiguous base calls, and passing contamination checks (http://dx.doi.org/10.17504/protocols.io.bfc3jiyn).<br><br>This adherence to standardized consensus sequence standards, combined with the presence of shared rare SNPs (consistent with the Machkovech et al. framework), ensures that our results reflect true viral continuity rather than residual RNA shedding or artifacts.	Line 436-445
Line 131: What was the reason for the time cutoff for no additional tests in the control group within 120 days? Should a control not also be included if a test has been performed and the sequences are divergent enough?	The 120-day window was established to ensure the clinical resolution of the acute episode and to strictly exclude the possibility of late-stage viral rebounding or including reinfections. This duration exceeds the standard 90-day threshold typically used by public health authorities (e.g., the ECDC and Statens Serum Institut in Denmark) to define a new infection episode.<br><br>By extending this to 120 days, we maximized the specificity of our control group, ensuring that individuals labeled as controls had both a documented negative test and a prolonged period of "silence" before any subsequent reinfection could occur. We have clarified this in the	Line 576-579 (Methods)

	Methods.	
Line 212: Why restrict the analyses to sequences with at least 1 non-syn change. Why not just use all 'final' sequences?	We restricted this analysis to sequences with at least one non-synonymous change to focus specifically on the transmission of viruses that evolved during the persistent infection. Including sequences with zero changes would primarily reflect the transmission of the original founder virus, which precludes the study of within-host mutations and their role in downstream population-level diversification.	
Line 140 and following: While the higher risk of prolonged infections in immunocompromised individuals is a quite well-established fact, the reasoning to only analyse pre-2020 diagnoses is unclear to me. To identify it as a risk factor, the immunosuppression should be present at the time of infection. The added value of the contemporary analysis is not clear to me, since it ends (31st of December 2021) before many of the later persistent episodes; please clarify what additional information it provides.	We used pre-pandemic diagnoses to establish a clear temporal precedence between the risk factors and the onset of infection (Supplementary Figure 5). This approach prevents reverse causality, where the infection itself could potentially lead to the diagnosis of conditions such as diabetes or secondary immunosuppression.<br><br>From a causal inference perspective, adjusting for conditions diagnosed after the infection would risk blocking the causal pathway or introducing bias if those conditions act as mediators rather than baseline confounders. We included the contemporary analysis up to December 2021 as a sensitivity check to ensure that our findings remained robust when accounting for more recent medical histories, even though this introduces a more complex temporal relationship. Diagnostic information beyond December 2021 was not available.	
Line 168 following: The authors show cumulative number of mutational changes over all identified samples. While this is sufficient to give an overall overview over the number of syn to non-syn mutations, Figure 2A and the corresponding text could be misleading. The number of changes per variant is misleading as the number of mutations per site depends on the time difference between consecutive samples. The authors indeed show differences in the infection time (Figure 1 B). More changes	While we agree that sampling intervals influence the total number of observed changes, Figure 2a is intended as a descriptive overview of the genomic distribution of mutations observed across persistent infections, rather than a comparison of variant-specific evolutionary rates. To do the latter one would have to perform extensive phylogenetic analyses. Consequently, the accumulation of changes in specific variants, such as BA.2, reflects the overall	Line 169-172

for BA.2 could simply be attributed to the sampling timeframe. Notably, this is not stated in the text, but the figure implies it.	mutational diversity captured within our dataset for those variants, and should not be interpreted as evidence of faster mutation accumulation.<br><br>We have clarified this interpretation in the text. To explicitly account for differences in sampling interval and infection duration, we use zero-inflated negative binomial regression models later in the manuscript to evaluate mutational burden while adjusting for time between samples, age, and variant.	
Line 170 following: Here the sampling timeframe is also not considered. If I am not mistaken, the raw cumulative mutation counts were divided by the length of the individual genes. The authors should consider the time difference between consecutive samples and evaluate this per sequence pair to get a real estimate of a mutation rate per gene. Then the authors can test whether the presented differences are statistically significant.	We agree that accounting for the sampling timeframe is essential for estimating true mutation rates. While Figure 2c provides a descriptive overview of the cumulative mutational burden across specific genes, we perform the formal, time-adjusted analysis later in the manuscript. This approach allows us to determine the rate of synonymous and nonsynonymous substitutions while accounting for both the duration of infection and the high proportion of cases with zero consensus-level changes. It also allows us to compare values directly with Ghafari et al. (Figure 2b: https://www.nature.com/articles/s41586-024-07029-4/figures/2)<br><br>We acknowledge, however, that the text could be misinterpreted. We have therefore amended the main text to avoid any confusion and thank the reviewer for pointing this out.	Line 172 (Results)
Supplementary Figure 2: The authors distinguish Supplementary Fig. 2 ('all pairs of consecutive sequences') from Fig. 2B ('first vs last sequence only'), but visually the patterns look very similar, and it's not obvious what additional biological insight is gained by showing both. It might be worth clarifying the motivation for keeping both panels.	We agree that the patterns look very similar, but we kept Supplementary Figure 2 for the sake of completeness and to have a direct reference for the numbers noted in the text. Because they are visually similar, we moved the figure into the supplementary rather than keeping both versions in the main text; but we would also be happy to convert it to a table to avoid the visual similarities. We defer to the Editor for guidance on this.	

Line 183 onward: Currently the authors only test whether there are differences between both groups in their Ka/Ks differences per gene. However, the approach cannot detect site specific adaptations. Positive selection of individuals with immunosuppression should correlate with some sort of selection pressure. Therefore, the manuscript would benefit from site-specific models. It would be highly interesting if the authors can correlate positive selected sites with the observed mutations that likely confer resistance to antiviral treatment.	We appreciate the suggestion to employ site-specific selection models. However, such analyses require a high density of substitutions at individual codons to achieve statistical significance. Given the sparsity of mutations across our cohort, a site-specific approach would lack the necessary power and remain prone to stochastic noise. We instead utilized a gene-level framework to aggregate the evolutionary signal across entire coding regions, which allowed for a more robust identification of selective pressures while maintaining statistical integrity.	
Line 313 and following: (I) Which COVID-19 database are the authors referring to? (II) The matching analysis compares final sequences of persistent infections to other sequences within an 11 day window. While the authors show that many of these sequences have zero, one, or two differences this is difficult to interpret without a proper baseline: with dense sequencing, exact or near exact matches are expected. Without explicit reconstruction of transmission clusters, the evidence for onward transmission remains suggestive.	Regarding (I) We have clarified the text in the first line of the discussion to avoid confusion, noting that it is simply referring to data collected in Denmark as a whole.<br><br>Regarding (II): We agree that the text was misleading. As mentioned in a response to Reviewer 1, we have revised the text (both in the Results and in the Discussion) to tone down this claim, clarifying that these findings simply show that persistent infections remain genetically consistent with the broader viral population, which is a necessary, albeit not sufficient, condition for onward transmission.	Line 325<br><br>Line 192-200 (Results)<br><br>Line 409-411 (Discussion)
Line 247 and following: Are higher number of non-syn mutations special to long-term infections or something that is variant specific? Can the authors clarify with their model if the increase in the number of non-syn changes between the alpha and remaining variants is something that is also mimicked when analysing the global pyhlogeny?	We thank the reviewer for this insightful question. Our analysis indicates that the higher number of nonsynonymous mutations in later variants is indeed a biological signal rather than an artifact of sampling time. This finding aligns with other studies looking at persistent infections, where treatment is directly linked with nonsynonymous substitutions (https://doi.org/10.1371/journal.ppat.1012624, https://academic.oup.com/ve/article/11/1/veaf054/8209747?login=false, https://www.nature.com/articles/s41579-022-00841-7). Admittedly, however, few studies have looked specifically at within-host differences by variant.	

Line 265 and following: That the number of mutations in older age groups is lower than in younger age groups is intriguing. The authors give immune senescence as a potential reason. However, their identified risk factor is immunosuppression. Should the viral evolution in all cases then not be independent of immune pressure? This data is not easy to explain biologically and might need a more detailed discussion.	We appreciate the opportunity to clarify this distinction. While both advanced age and clinical immunosuppression are risk factors for viral persistence, they exert different effects on viral adaptation. In older age groups, immunosenescence creates a permissive environment where the virus can persist with minimal selective pressure, leading to fewer nonsynonymous changes.<br><br>In contrast, individuals with clinical immunosuppression (such as those with cancer or HIV) often receive intensive, targeted treatments like monoclonal antibodies or antivirals. These interventions, combined with higher residual immune activity, provide the specific selective pressures that drive rapid nonsynonymous evolution. Our data suggests that while age leads to "passive" persistence with low mutational turnover, clinical immunosuppression often results in "active" persistence characterized by treatment-driven adaptation.	
Line 296: Antibodies and antivirals were only in use during specific timeframes. In the absence of data for antiviral treatment it would be interesting if the timeframes match, e.g. Molnupiravir resistances in an Alpha variant would not make sense. Can the authors match resistance mutations and sampling times to drug availability?	This is an excellent suggestion. We have added a Supplementary table and some corresponding text; fortunately, the dates do in fact line up with treatment periods, further highlighting the plausibility of the link.	Line 245<br><br>Supplementary Table 11

Reviewer 3
General comment: Thank you for the opportunity to review this manuscript. Persistent SARS-CoV-2 infections are thought to play an important role in the emergence of novel viral variants, and the study by Khurana et al. provides valuable large-scale data to further investigate these processes. While several findings are consistent with prior reports, such as the strong association between persistent infection and immunocompromising conditions, the size and scope of the cohort are unique and benefits from patient registry information. My main comments focus on methodological choices related to the definition of persistent infection, sequencing platforms, and control selection, as detailed below.

Comments	Author response	Location of change

Sequence pairs within the same variant sampled at least 26 days apart are considered indicative of potentially persistent infections. This approach may exclude true persistent infections that undergo substantial sequence evolution resulting in clade or variant misclassification over time.	We completely agree, and this is the inherent tradeoff between capturing large-scale viral evolution but also ensuring that these are not in fact reinfections, which would bias results.	
There is potential for sequencing errors associated with the MinION platform, particularly for R9.4.1 flow cells. Given the use of multiple sequencing platforms, it would be helpful to clarify whether any persistent infection cases had samples sequenced on different platforms, and if so, how platform-specific error profiles were accounted for.	We completely agree that this would be interesting; unfortunately, however, the specific platform used for each consensus sequence generation is not available, precluding us from comparing samples in this way.	
Separating persistent infection from reinfection is challenging. Is information on intervening negative tests available, or are persistent infections identified solely as uninterrupted runs of positive tests? The likelihood of reinfection increases with longer time intervals, and this should be addressed more explicitly.	Thanks to the reviewer for the comment. Indeed, negative tests were available; in the manuscript, we explicitly model this dynamic as viral 'rebounding' (see paragraph immediately preceding the Discussion).	Line 312-322
Persistent infections appear to be defined, in part, by the presence of shared rare SNPs across consecutive time points relative to the background population for a given major variant. This approach could be problematic because persistent infections can undergo rapid intra-host evolution, potentially reducing the number of shared SNPs over time, particularly in cases with sparse sampling. While the approach is stringent and helps ensure that sequences represent the same infection, it may bias the analysis toward a specific subset of cases, especially when characterizing the mutational landscape and onward transmission potential of persistent infections.	We acknowledge that our stringent requirement for shared rare substitutions may exclude cases characterized by extreme intra-host diversification or those with very sparse sampling. However, this criteria was intentionally selected to ensure the highest possible specificity in a large-scale national dataset where the risk of misclassifying rapid reinfections as persistence is significant. While this may prioritize a subset of persistent infections with higher genomic stability, it prevents the inclusion of false positives that would otherwise inflate our estimates of mutational accumulation and distort our risk factor analysis.	
Controls are defined as individuals with a negative test within 26 days of a	We believe that matching cases and controls by test date is the most robust approach to	

positive result and no additional positive tests within the subsequent 120 days. However, the ability to detect persistent infection depends strongly on testing frequency. The authors should assess whether testing rates (positive and negative) are comparable between cases and controls, and consider further stratifying controls to include individuals with similar testing intensity but no evidence of persistent infection.	address this, as it ensures both groups were subject to the same background testing availability and public health guidelines at any given time during the pandemic.<br><br>We do not believe that controlling for testing frequency after the index date would be appropriate, as testing behavior in Denmark was heavily dictated by clinical status and 'Coronapas' requirements (a COVID passport necessary to enter restaurants, etc. https://www.bbc.com/news/world-europe-56812293).<br><br>Individuals who test positive naturally face increased monitoring and specific incentives to re-test to re-enter society, whereas those who test negative have no such incentive. Because post-infection testing is a direct consequence of the infection status itself, stratifying by this intensity would introduce significant confounding. Furthermore, using previous testing history as a proxy is often confounded by unmeasured sociodemographic factors (e.g., occupation) rather than the biological risk of persistence.	
A high proportion of elderly patients is observed among the identified persistent infection cases. This may reflect slower viral clearance and prolonged PCR positivity in older individuals rather than true persistence. Variable recovery times by age should be considered and discussed.	We agree that slower viral clearance in the elderly is a valid concern. While our genomic criteria confirm that samples belong to the same infection event, we acknowledge that prolonged PCR positivity may not always reflect active viral replication. We have added this to the Limitations section.	Line 456-458
The onward transmission analysis is difficult to interpret. Because the selection criteria for persistent infection cases enrich for sequences containing rare SNPs, it is unclear how likely it is to observe the reported number of similar sequences by chance alone in the dataset. Is it possible to formally assess this likelihood? An orthogonal approach, such as assessing whether related sequences are also geographically clustered, would also strengthen the conclusions if such data are available.	We thank the reviewer for the comment. We agree that the selection criteria for persistent infection cases could enrich for rare SNPs, and that this makes the interpretation of similarity counts challenging without an additional reference frame. To address this concern, we implemented the suggested orthogonal, geography-based assessment of the matches.<br><br>Specifically, we examined the regional distribution of exact sequence matches (0 differences) between persistent cases and database sequences. As reported in the newly	Lines 192-196<br><br>Supplementary Table 6

	added Supplementary Table, 10 persistent cases matched exactly to 33 database sequences, with the majority of these matches concentrated within the same Danish regions as the persistent infections. This supports the plausibility of onward transmission from persistent infections. We have added this to the Results section, and included the new Supplementary Table.	
Minor comments<br><br>1. While analyzing spike mutations as a whole is reasonable, it would be helpful to provide more detailed information on mutations within key antigenic regions, particularly the N-terminal domain (NTD) and receptor-binding domain (RBD).<br><br>2. No minor intra-host variant data are presented. Many mutations may exist at sub-consensus frequencies and therefore would not be detected in consensus sequences. This limitation should be acknowledged explicitly.<br><br>3. While I understand the complexities of dealing with individual-level data in large cohorts, I wonder if at least the sequence data for persistent infection cases could be made available with limited metadata.<br><br>While the authors have done a commendable job of making the analysis code available, the lack of underlying data complicates reproducibility. I understand the complications in making individual-level data available but there are analyses presented in the manuscript that do not rely on individualized data. For other analyses, pre-processed aggregate data could be provided instead.	1. We agree that this would be useful. We have now added labels to Supplementary Tables 9 and 10, which maps the recurrent mutations to highlight whether each spike mutation belongs to the RBD or NTD domains.<br><br>2. We agree and have added this to the Limitations section.<br><br>3. We agree that this is a tricky tradeoff. We would like to clarify that all consensus genomes from this study are indeed available to the public. Denmark has one of the highest per-capita sequencing and sequence sharing rates in the world (https://www.nature.com/articles/s41467-022-33713-y), and all sequences have been deposited in GISAID. We have added an accession number for all sequences in Denmark in this time period in the Data Availability statement.<br><br>However, while the sequences are public, Danish data protection laws prevent us from publishing a file that links specific GISAID accessions to sensitive individual data (even including specific sampling dates for individual people), because individuals in the cohort have various health diagnosis including HIV and diabetes, which could potentially be used to re-identify individuals, which is against local regulations.<br><br>We have also now added synthetic	Lines 434-436<br><br>Supplementary Table 9 & 10<br><br>Data availability statement

	data to the GitHub repository such that the functionality of the code for the figures can be verified.	

Reviewer 4

General comment: The authors describe their work analyzing the extensive SARS-CoV-2 genomic surveillance data from Denmark (>700,000 genomes) to identify and characterize persistent infections (n=303). The authors were comprehensive in their extensive analyses and convincing in their efforts to connect a correlative association of immunosuppression as a driver of mutation dynamics in those individuals with persistent SARS-CoV-2 infection.

The authors show that older individuals had higher odds of persistence than younger groups and show that those who are immunocompromised were at highest risk of developing persistent infections relative to other co-morbidity states (over 5 times the risk, line 322). A strong point in their analysis is that they conducted matched case-control study for each persistent infection to examine possible risk factors for persistent infection.

The authors provide data to support the idea that immunosuppression coupled with SARS-CoV-2 monoclonal (mAB) or antiviral treatment was associated with significantly higher numbers of nonsynonymous mutations (line 282), particularly mutations associated with mAB resistance. Although specific link to actual treatment therapies was not provided but rather inferred from the data (immunosuppression and/or immunocompromised diagnosis), the authors were correct to point out that while nonsynonymous mutations increased in immunosuppressed synonymous mutations did not, indicative of selective pressure (presumably from mAB/antiviral therapies). Notably, the authors point out that while older aged-groups had higher odds of persistent infection, they showed fewer within-host substitutions, both nonsynonymous and synonymous (lines 337-338). This paradox was attributed to immune senescence that would impair immune responses leading to prolonged infections with less selective pressure and therefore less viral diversity, as observed.

This study demonstrates the power of SARS-CoV-2 genomic surveillance data when integrated with clinical and epidemiologic data to understand population-level factors influencing viral evolution. The authors present a convincing set of analyses from Denmark's comprehensive and unique datasets that links immunosuppression and presumed antiviral treatment as a driver of selective pressure to effect viral mutation dynamics.

Comments	Author response	Location of change
I wonder about the inclusion criterion of Ct values between 30 and 38 for whole genome sequencing. In my experience, it has been difficult to obtain high quality sequence data from samples with higher Ct values, particularly Ct >32. Can the authors please comment on this and explain how these higher Ct value samples yielded adequate sequence data for this	We acknowledge that high Ct values (low viral loads) can pose challenges for WGS; however, as discussed in our response to Reviewer 1, all samples in this study were processed through the standardized Statens Serum Institut (SSI) pipeline, which was optimized to generate high-quality genomic data for samples with Ct values up to 38. We did not include samples based on Ct alone, but rather on their ability to pass stringent DCGC quality control (QC)	Line 436-445

study?	requirements, including fewer than 3,000 Ns and $\leq$ 5 ambiguous base calls. The successful generation of a high-quality consensus genome at these higher thresholds provides empirical evidence of intact viral RNA—distinguishing these cases from the degraded "residual shedding" that typically fails sequencing. Furthermore, as noted in our previous response, our identification of persistence relies on shared rare SNPs, a method that confirms genetic continuity regardless of the initial viral load. We have also addressed the potential implications of including these higher Ct samples in the revised Limitations section.	
I'm curious about the authors seeming to equate fitness of recurrent within-host nonsynonymous mutations with enhanced transmission between hosts as stated here:<br>Lines 355-358 paraphrased: Many "…recurrent nonsynonymous mutations within a host have a higher between-host fitness on average compared to single-occurring and unrealized mutations at the same sites."<br>Is this statement related to Figure 4? If so, can you help me understand this in the context of Figure 4? Furthermore, is this<br>Please also note, that for me, Figure 4A colors in the graph do not correspond to colors in the legend – please enhance and reconcile this figure accordingly.	We thank the reviewer for the opportunity to clarify this distinction. We do not assume that within-host and between-host fitness are inherently the same; rather, our analysis uses independent, globally-inferred fitness scores from the Bloom et al. model to test if an overlap exists. As shown in Figure 4, we observe that mutations arising recurrently across multiple persistent hosts have significantly higher between-host fitness scores than those occurring only once. This suggests that the selective environment within a persistent infection frequently favors the same mutations that are successful in global transmission, rather than solely producing host-specific or deleterious variants. We have now clarified in the text that the fitness values were independently derived to make this clearer.<br><br>We have also double checked to ensure that the colours in Figure 4A are the same as those in the legend.	Lines 365-368<br><br>Figure 4
Line 353: "Notably, genomes of individuals with immunosuppression harbored significantly more nonsynonymous changes upon infection"<br>Was this shown or discussed in the text prior to this line? Please elaborate – this statement seems to say that immunosuppressed individuals were infected by virus with more nonsynonymous mutations than non-immunosuppressed persons. Is this	We thank the reviewer for the comment. To clarify, this finding refers to the accumulation of nonsynonymous mutations over the duration of a persistent infection, rather than the state of the virus at the moment of initial transmission. As detailed in the Results section, our model adjusts for the time between samples to specifically measure the rate of within-host change. We have updated the wording in the Discussion to specify that these mutations were acquired during the course of infection to avoid any ambiguity.	Lines 360-362

what you mean, that immunosuppressed persons start an infection with more mutated virus? If not, please update the wording to reflect what you mean.		
This study focused on consensus sequence data, but I'm curious if you were able to identify any subconsensus substitutions in earlier sequence that became fixed at later sampling time?	Unfortunately the subconsensus level data was not available to us for analysis, although we acknowledge that this would've strengthened the article. We now mention this in the Discussion.	Lines 435-437
Line 223 refers to Figure 3D – but the manuscript has no such figure.	We thank the reviewer for spotting this. We have now changed the text to refer to Figure 2 instead of Figure 3.	
Lines 219 – 240 – describes the substitutions detected. As a favor to the reader, it would be quite helpful to include these data in a table.	We have included these as tables in the Supplementary (Supplementary Tables 9 and 10), but would be happy to move this into the main text; we invite editorial guidance on whether we should go ahead with this.	Supplementary Table 9 and 10
I found the following sentence (lines 300 – 304) a bit awkward – suggest rewording it for clarity:<br>"58.3% (7/12) of individuals with a monoclonal antibody resistance-linked substitution—specifically among those with infections ending prior to mid-2022, out<br>of a total of 34 individuals (including those with infections ending beyond this point),<br>which is the cut-off for available hospitalization data—were hospitalized at some point during their infection."	We completely agree that this could be written in a much clearer manner. We have adjusted the wording in the text.	Lines 309-311
Line 353: "Notably, genomes of individuals…" update this passage to include "viral" as follows:<br>"Notably, viral genomes of individuals…"	We thank the reviewer for spotting this and have amended it in the text.	Line 360
Line 360: Suggest inserting "immunosuppressed" into the sentence as follows –<br>"…repeatedly within immunosuppressed hosts…"	We have now added this to the text.	Line 369
Line 426 – you mention not examining	Thanks to the reviewer for pointing us in the	Reference

subconsensus sequence; suggest you look at a recent study by Bell, et al., who did a similar examination of persistent infections from a large data set but did examine subconsensus sequence data.<br>Bell JM, Elder J, Ryder R, Smith EA, Scribner M, Gilliam S, et al. (2025). Genomic and epidemiologic characteristics of SARS-CoV-2 persistent infections in California, January 2021 – July 2023. PLOS Pathogens, 21(11): e1013365.https://doi.org/10.1371/journal.ppat.1013365	direction of this paper! We have also now cited it in the manuscript.	67
Line 496: curious how you decided on the <120 days apart metric?	The 120-day window was established to ensure the clinical resolution of the acute episode and to strictly exclude the possibility of late-stage viral rebounding. This duration exceeds the standard 90-day threshold typically used by public health authorities (e.g., the ECDC and Statens Serum Institut in Denmark) to define a new infection episode. By extending this to 120 days, we maximized the specificity of our control group, ensuring that individuals labeled as controls had both a documented negative test and a prolonged period of "silence" before any subsequent reinfection could occur. We have clarified this in the Methods.	Lines 576-580
Lines 121-122 and 590-591: Ct values are correlated to viral load, but that is an incorrect assumption; suggest using another term instead of viral load…perhaps viral RNA load?	We completely agree, and have therefore clarified that it refers to viral RNA load.	Line 124 (Main text)<br><br>Line 614 (Methods)
Lines 653-654: data availability of consensus genomes is restricted due to what the authors say pertains to Danish data protection laws. This is counter to the SARS-CoV-2 global initiative to share at least sequence data for public access without compromising individual privacy. Does this mean that there are no Danish SARS-CoV-2 sequence data available publicly via GISAID or other public data repository?	We would like to clarify that all consensus genomes from this study are indeed available to the public. Denmark has one of the highest per-capita sequencing and sequence sharing rates in the world (https://www.nature.com/articles/s41467-022-33713-y), and all sequences have been deposited in GISAID. We have added an accession number for all sequences in Denmark in this time period in the Data Availability statement.<br><br>However, while the sequences are public,	Data availability statement

	Danish data protection laws prevent us from publishing a file that links specific accessions for the 303 individuals in this study, which includes potentially sensitive individual data (even including specific sampling dates among study participants here), because individuals in the cohort have various health diagnoses including HIV and diabetes. In turn, this could potentially be used to re-identify individuals, which is against local regulations. We have also amended the Data Availability statement to clarify this.	

Reviewer 1

General comment: The authors have fully addressed all of my concerns. This is a great study! The code is well organized and is a usable resource for the community.

- **RESPONSE:** We thank the reviewer for these kind comments and for taking time reviewing our manuscript.

Reviewer 2

General comment: I thank the authors for the updates and additions to the manuscript. My comments and questions have sufficiently been addressed.

- **RESPONSE:** We thank the reviewer for their feedback and their time!

R.2.1

I have also assessed the responses to Reviewer 3's comments. In my opinion, all comments were adequately addressed with the exception of point 3:

The reviewer asked how negative tests influence the detection of long-term infection. It stands to reason that negative tests in between positive tests should be considered in the detection of long-term infection as this could also indicate reinfection. The authors state that they model rebounding frequency (positive test – negative test – positive test) to analyse if this is influenced by immunosuppression. However, this is not what the critique was targeting at. I actually agree that if information about negative tests is available, they should be considered in the initial definition of a long-term infection.

- **RESPONSE:** We thank the reviewer for pointing this out, and apologize for not clarifying it in the previous response. We completely agree that negative tests, in general, could indicate a possible reinfection. However, there is a tradeoff between sensitivity in detecting reinfections and maintaining a consistent definition of the 'same' infection. In this case, we rely on the rare SNP definition to try and balance the two: even if there is a single negative test during the course of an infection, the rare SNP requirement should significantly reduce the likelihood of this being a reinfection (since we would not expect the same rare SNP to be present across two unique infection episodes). In a hypothetical case where there were no identical rare SNPs across samples, then the individual would not be included as a persistent infection by our definition and would have been excluded - therefore indicating a reinfection. As such, the rare SNP definition mitigates the issue of reinfection, although we concede that no detection method is perfect in distinguishing between the two. We have added text to the Limitations section to make this more explicit.
- **CHANGES:**
  - *Limitations: "Some persistent infections included intervening negative RT-PCR tests between positive results, which could in principle reflect reinfection rather than true persistence. However, our requirement that consecutive sequences share rare SNPs substantially reduces this risk, as independently acquired infections would not be expected to carry the same rare polymorphisms. Cases lacking shared rare SNPs across samples were excluded, thereby filtering out likely reinfections."*

Reviewer 4

R.4.1

The authors have satisfactorily addressed my major concerns. However, the coloring of the histograms in Figures 2f, 3, and 4 remains an issue. The bars contain yellow or green gradients that are not reflected in the legend. To ensure clarity for the reader, I suggest the authors use consistent coloring that corresponds

with the provided legend. My computer systems do not allow me access to github sites, so I am not able to review the code.

- **RESPONSE:** We agree that the colouring might not be consistent between plots, so we opted to use the ubiquitous “viridis” colouring scheme which is perpetually uniform (https://www.nature.com/articles/d41586-021-02696-z). For figure 2f, 3 and 4 the colouring changes are not due to a visualisation choice but because of an overlap of histograms. We believe it is important to show this overlap, and it is not possible to do this without a visual change in colour for the overlapping region. We believe this is standard practice. We would value editorial guidance on how to align with current *Nature Communications* graphics practice.

ROUND 1 REVIEWER 1 ATTACHMENT:

Khurana et al. leveraged Denmark's extensive genomic surveillance to identify persistent infections and used associated health and demographic factors as part of a case-control study to determine risk factors associated with persistent infections. They identified 303 persistent infections. Older adults, unvaccinated individuals, and immunosuppressed individuals were all at greater risk of persistent infections. They also detected positive selection in Spike, and recurrent mutations were in monoclonal antibody/antiviral resistance sites and sites detected in other immunocompromised individuals. It is critical to understand persistent SARS-CoV-2 infections and the risk factors for persistent infections because new variants and immune escape mutations appear to originate from persistent infections. Large genomic surveillance efforts with sufficient metadata are a great resource for understanding persistent infection, especially when combined with matching control cases. It is clear that a lot of work has gone into this study.

Major Comments

1. My biggest concern with this study is the definition of persistent infections used in this study. They used a very similar method to Ghafari *et al*. However, Ghafari *et al.* only used samples with a Ct value of ≤30, whereas this study used a cutoff of ≤38. High Ct samples are less likely to reflect an active infection. The higher Ct threshold is likely to result in the identification of more false persistent infections. How does the data compare if the Ct threshold was lowered? Are many of the persistent infections no longer identified? From Figure1F, it looks like most of the last samples have Ct values greater than 30.

2. Matching last the sequence of persistent infections to genetically similar acute sequences isn't strong evidence of the plausibility of onward transmission. Within the 11 day time frame many epidemiologically unlinked but genetically similar acute infections are present, so it isn't surprising that some persistent infections are very similar to what is circulating. Additionally, for the persistent samples that have 1 or 2 mismatches with acute samples, the mismatches may just be the initial sequence of the persistent infection. I am not arguing that onward transmission from persistent infections is not plausible, just that this analysis doesn't add to what we know.

Minor comments

1. Lines 122-124; Ct values aren't a direct indicator of sequence quality. It is hard to get high quality sequences from low viral load samples, but there are cases when you do, and many low Ct value samples don't result in high quality sequencing.

2. You should cite Raglow *et al*. 2024 (https://www.thelancet.com/journals/lanmic/article/PIIS2666-5247(23)00336-1/fulltext?uuid=uuid%3A5679b141-e4e6-47fe-8378-759a73a7e84d). It is a cohort study of hospitalized immunocompromised individuals and the conditions to promote persistent infections.

3. Figure 1 AB and lines 251-256. The Ct range that was sequenced by DCGC varied over the course of the pandemic (maximum Ct 30-38). Could this influence the patterns when comparing the different variants?
