# Supplementary material for: Large-scale genomic surveillance reveals immunosuppression drives mutation dynamics in persistent SARS-CoV-2 infections
Source: Nat Commun. 2026 Jun 19;17:7730. doi: 10.1038/s41467-026-74445-7 (PMC13433919; doi:10.1038/s41467-026-74445-7)
Supplement: Supplementary file 3 [file 41467_2026_74445_MOESM3_ESM.pdf]

nature portfolio

Corresponding author(s): Mark P. Khurana

Last updated by author(s): Nov 14, 2025

Reporting Summary

Nature Portfolio wishes to improve the reproducibility of the work that we publish. This form provides structure for consistency and transparency in reporting. For further information on Nature Portfolio policies, see our Editorial Policies and the Editorial Policy Checklist.

Statistics

For all statistical analyses, confirm that the following items are present in the figure legend, table legend, main text, or Methods section.

n/a	Confirmed	
☐	☒	The exact sample size (*n*) for each experimental group/condition, given as a discrete number and unit of measurement
☐	☒	A statement on whether measurements were taken from distinct samples or whether the same sample was measured repeatedly
☐	☒	The statistical test(s) used AND whether they are one- or two-sided<br>*Only common tests should be described solely by name; describe more complex techniques in the Methods section.*
☐	☒	A description of all covariates tested
☐	☒	A description of any assumptions or corrections, such as tests of normality and adjustment for multiple comparisons
☐	☒	A full description of the statistical parameters including central tendency (e.g. means) or other basic estimates (e.g. regression coefficient) AND variation (e.g. standard deviation) or associated estimates of uncertainty (e.g. confidence intervals)
☐	☒	For null hypothesis testing, the test statistic (e.g. *F*, *t*, *r*) with confidence intervals, effect sizes, degrees of freedom and *P* value noted<br>*Give P values as exact values whenever suitable.*
☐	☒	For Bayesian analysis, information on the choice of priors and Markov chain Monte Carlo settings
☐	☒	For hierarchical and complex designs, identification of the appropriate level for tests and full reporting of outcomes
☐	☒	Estimates of effect sizes (e.g. Cohen's *d*, Pearson's *r*), indicating how they were calculated

Our web collection on statistics for biologists contains articles on many of the points above.

Software and code

Policy information about availability of computer code

Data collection	Sequencing software and functions used during the collection and analysis of PCR tests: Guppy v.3.6.1, ARTIC v.3, MinION device, R.9.4.1 flow cells, SQK-LSK109 ligation kit, Oxford Nanopore, ARTIC network protocol v.1.1.0, Medaka v.1.0.3, NextSeq platform, NovaSeq platform, trim-galore, iVar, BCFtools.
Data analysis	R (4.2.3), Python (3.10.10) was used for coding. IQTREE ((v1.6.12)), MAFFT (v.7.520) were used for phylogenetic analysis.

For manuscripts utilizing custom algorithms or software that are central to the research but not yet described in published literature, software must be made available to editors and reviewers. We strongly encourage code deposition in a community repository (e.g. GitHub). See the Nature Portfolio guidelines for submitting code & software for further information.

Data

Policy information about availability of data

All manuscripts must include a data availability statement. This statement should provide the following information, where applicable:

- Accession codes, unique identifiers, or web links for publicly available datasets
- A description of any restrictions on data availability
- For clinical datasets or third party data, please ensure that the statement adheres to our policy

All code relevant to reproduce the experiments is available online: https://github.com/MLGlobalHealth/sars-cov2-persistent.

Research involving human participants, their data, or biological material

Policy information about studies with human participants or human data. See also policy information about sex, gender (identity/presentation), and sexual orientation and race, ethnicity and racism.

Reporting on sex and gender	We describe the sex breakdown in Figure 1 and in the supplementary.
Reporting on race, ethnicity, or other socially relevant groupings	We do not report on race, ethnicity or other social groupings. This was not relevant for our analysis.
Population characteristics	To link sequences with Danish registry data, pseudo-anonymized Danish civil registration numbers (CPR-numbers) were used to link sequences to their corresponding registry information stored at Statistics Denmark. Registry information included sex, age, test date, vaccination status, medical diagnoses, and dates.
Recruitment	Sequencing SARS-CoV-2 was systematically done as part of Denmark's COVID-19 response. Denmark implemented a dual testing strategy for SARS-CoV-2, dividing testing into healthcare and community tracks with the goal of curbing transmission, safeguarding vulnerable populations, and preventing the overload of healthcare infrastructure. The healthcare track initially analyzed all samples and later focused on clinical testing, including both in- and out-patients. In addition to the healthcare track, Denmark established the community track to offer free, on-demand testing without referrals. During the pandemic, trained professionals collected oropharyngeal swabs for PCR testing at various testing stations established across the country, while self-administered home tests only became routine by December.
Ethics oversight	This study was conducted using data from national registers only. According to Danish law, ethics approval is not needed for this type of research. All data management and analyses were carried out on the Statistics Denmark's secure research servers. The study only contains aggregated results and no personal data.

Note that full information on the approval of the study protocol must also be provided in the manuscript.

Field-specific reporting

Please select the one below that is the best fit for your research. If you are not sure, read the appropriate sections before making your selection.

☒ Life sciences ☐ Behavioural & social sciences ☐ Ecological, evolutionary & environmental sciences

For a reference copy of the document with all sections, see nature.com/documents/nr-reporting-summary-flat.pdf

Life sciences study design

All studies must disclose on these points even when the disclosure is negative.

Sample size	738944
Data exclusions	We excluded cases without high-quality consensus sequences (Ct > 38), infections from variants other than Alpha, Delta, and Omicron (BA.1, BA.2, BA.5), and potential persistent infections that did not share rare SNPs between sequence pairs. In total, 152 possible persistent infection cases were removed for not meeting the rare SNP-based filtering criteria.
Replication	Not relevant.
Randomization	Not relevant.
Blinding	Not relevant.

Reporting for specific materials, systems and methods

We require information from authors about some types of materials, experimental systems and methods used in many studies. Here, indicate whether each material, system or method listed is relevant to your study. If you are not sure if a list item applies to your research, read the appropriate section before selecting a response.

Materials & experimental systems

n/a	Involved in the study
☒	☐ Antibodies
☒	☐ Eukaryotic cell lines
☒	☐ Palaeontology and archaeology
☒	☐ Animals and other organisms
☒	☐ Clinical data
☒	☐ Dual use research of concern
☒	☐ Plants

Methods

n/a	Involved in the study
☒	☐ ChIP-seq
☒	☐ Flow cytometry
☒	☐ MRI-based neuroimaging

Plants

Seed stocks

Report on the source of all seed stocks or other plant material used. If applicable, state the seed stock centre and catalogue number. If plant specimens were collected from the field, describe the collection location, date and sampling procedures.

Novel plant genotypes

Describe the methods by which all novel plant genotypes were produced. This includes those generated by transgenic approaches, gene editing, chemical/radiation-based mutagenesis and hybridization. For transgenic lines, describe the transformation method, the number of independent lines analyzed and the generation upon which experiments were performed. For gene-edited lines, describe the editor used, the endogenous sequence targeted for editing, the targeting guide RNA sequence (if applicable) and how the editor was applied.

Authentication

Describe any authentication procedures for each seed stock used or novel genotype generated. Describe any experiments used to assess the effect of a mutation and, where applicable, how potential secondary effects (e.g. second site T-DNA insertions, mosiacism, off-target gene editing) were examined.
